# Valproic acid upregulates the expression of the p75NTR/sortilin receptor complex to induce neuronal apoptosis

**DOI:** 10.1007/s10495-020-01626-0

**Published:** 2020-07-25

**Authors:** Simona Dedoni, Luisa Marras, Maria C. Olianas, Angela Ingianni, Pierluigi Onali

**Affiliations:** 1 Laboratory of Cellular and Molecular Pharmacology, Section of Neurosciences and Clinical Pharmacology, Department of Biomedical Sciences, University of Cagliari, 09042 Monserrato, CA Italy; 2 Section of Microbiology, Department of Biomedical Sciences, University of Cagliari, Cagliari, Italy

**Keywords:** Histone deacetylase inhibitors, p75NTR, Sortilin, Human neuroblastoma cells, Mouse cerebellar granule cells, Apoptosis

## Abstract

The antiepileptic and mood stabilizer agent valproic acid (VPA) has been shown to exert anti-tumour effects and to cause neuronal damage in the developing brain through mechanisms not completely understood. In the present study we show that prolonged exposure of SH-SY5Y and LAN-1 human neuroblastoma cells to clinically relevant concentrations of VPA caused a marked induction of the protein and transcript levels of the common neurotrophin receptor p75NTR and its co-receptor sortilin, two promoters of apoptotic cell death in response to proneurotrophins. VPA induction of p75NTR and sortilin was associated with an increase in plasma membrane expression of the receptor proteins and was mimicked by cell treatment with several histone deacetylase (HDAC) inhibitors. VPA and HDAC1 knockdown decreased the level of EZH2, a core component of the polycomb repressive complex 2, and upregulated the transcription factor CASZ1, a positive regulator of p75NTR. CASZ1 knockdown attenuated VPA-induced p75NTR overexpression. Cell treatment with VPA favoured proNGF-induced p75NTR/sortilin interaction and the exposure to proNGF enhanced JNK activation and apoptotic cell death elicited by VPA. Depletion of p75NTR or addition of the sortilin agonist neurotensin to block proNGF/sortilin interaction reduced the apoptotic response to VPA and proNGF. Exposure of mouse cerebellar granule cells to VPA upregulated p75NTR and sortilin and induced apoptosis which was enhanced by proNGF. These results indicate that VPA upregulates p75NTR apoptotic cell signalling through an epigenetic mechanism involving HDAC inhibition and suggest that this effect may contribute to the anti-neuroblastoma and neurotoxic effects of VPA.

**Electronic supplementary material:**

The online version of this article (10.1007/s10495-020-01626-0) contains supplementary material, which is available to authorized users.

## Introduction

Valproic acid (VPA) is a short-chain branched fatty acid widely used for its anticonvulsant, mood-stabilizing and analgesic properties [1, 2], More recently, clinical trials have shown that VPA displays anti-cancer activity in different types of tumours [3, 4]. The anticonvulsant and mood stabilizing effects have been classically attributed to VPA blockade of sodium and calcium channels, potentiation of inhibitory neurotransmission and modulation of intracellular kinase signalling [5, 6]. On the other hand, the anti-cancer activity of VPA has been mostly related to its ability to act as an inhibitor of histone deacetylases (HDACs), particularly those belonging to class I and II, thereby inducing histone hyperacetylation and removing HDAC-dependent transcriptional repression [7–9].

Exposure to VPA has been reported to exert either neuroprotective or neurotoxic effects. In a number of preclinical studies VPA was capable to protect the brain from multiple insults, such as β-amyloid toxicity, oxidative stress, stroke and traumatic brain injury, suggesting its potential for the treatment of different neurodegenerative diseases [6, 10, 11].

Conversely, there is also strong evidence that VPA can adversely affect neural growth and survival, and induce neurodegeneration, rather than neuroprotection, under both in vitro and in vivo experimental conditions. Thus, VPA was found to reduce hippocampal neurogenesis and to induce cognition deficits in rats [12]. Therapeutic concentrations of VPA were reported to cause cell death in a variety of neural cells, including neuronally differentiated PC12 cells, primary rat cortical neurons [13], cerebellar granule cells [14], progenitors of embryonic stem cell-derived glutamatergic neurons [15], and microglial cells [16]. In humans, prenatal exposure to VPA has been associated with neurodevelopmental defects and increased risk of autism spectrum disorder and childhood autism in the offspring [17, 18] Furthermore, a number of studies have demonstrated that VPA inhibits the growth and induces cell death of human neuroblastoma cells [19–22]. However, other studies have shown that the exposure to VPA promotes the proliferation and survival of neuroblastoma cells [23, 24].

While much is known on the molecular mechanisms mediating the neuroprotective actions of VPA [6, 10, 11], the cellular events involved in VPA-induced anti-tumour responses and neurodegeneration are not completely understood.

The common neurotrophin receptor p75NTR, a member of the tumour necrosis factor receptor superfamily, is a key regulator of neuronal cell fate in the developing and adult brain [25, 26]. In cells expressing the neurotrophin Trk tyrosine kinase receptors, which include the TrkA receptor of NGF, the TrkB receptor of BDNF and NT4, and the TrkC receptor for NT3, p75NTR can act as a co-receptor for each Trk and potentiates Trk signalling by enhancing the affinity and specificity for the cognate neurotrophin [27, 28]. In this mode of action, p75NTR supports the pro-survival activity of each neurotrophin. On the other hand, it has been observed that in cells lacking Trk or overexpressing p75NTR over Trk the activation of p75NTR can induce cell death through apoptosis [29, 30]. In this context, p75NTR forms a receptor complex with sortilin, a member of the Vps 10p-domain receptor family originally identified as the neurotensin receptor NTR3 [31]. It has been demonstrated that the p75NTR/sortilin receptor complex mediates the pro-apoptotic effects of the proneurotrophins proNGF and proBDNF [32, 33].

In the present study, we show that in human neuroblastoma cell lines and mouse primary neurons prolonged exposure to VPA upregulates the expression of a functional p75NTR/sortilin receptor complex and promotes proNGF-induced cell death through apoptosis.

## Materials and methods

### Materials

Cleavage-resistant mutated form of proNGF (K103A and R104A) was obtained from Alomone Labs (Jerusalem, Israel). Entinostat was obtained from Santa Cruz Biotechnology (Dallas, TX, USA). MC1568 was from SelleckChem (Houston, TX, USA). Romidepsin, PCI-34051, tubacin, 3-deazaneplanocin A hydrochloride (DZNep), and tazemetostat (TZM) were from MedChem Express Europe (Sollentuna, Sweden). VPA, trichostatin A (TSA), sodium butyrate, neurotensin, and 4’,6-diamidino-2phenylindole dihydrochloride (DAPI) were from Sigma-Aldrich (St. Louis, MO, USA).

### Cell culture

Human neuroblastoma cell lines SH-SY5Y and LAN-1 were obtained from the European Cell Culture Collection (Salisbury, UK) and grown in Ham’s F12/MEM medium (1:1) supplemented with 2 mM L-glutamine, 1% non-essential amino acids, 10% fetal calf serum (FCS) and 100 U/ml penicillin-100 µg/ml streptomycin (Sigma-Aldrich). Cells were maintained at 37 °C in a humidified atmosphere of 5% CO_2_ in air.

### Primary cultures of mouse cerebellar granule cells

CD-1 mice were obtained from Envigo RMS S.r.l. (S. Pietro al Natisone, Udine, Italy). Primary cultures of cerebellar granule cells were prepared from 7-day old mice of mixed sexes, using trypsin digestion (0.25% w/v), as previously described [34]. Dissociated cells were mixed with horse serum (final concentration 20%) (Euroclone, Milan, Italy) and collected by centrifugation. Cells were resuspended in Neurobasal-A medium containing N-2 supplement (Gibco-Thermo Fisher Scientific, Waltham, MA, USA), 0.5 mM L-glutamine, 50 µM β-mercaptoethanol and penicillin/streptomycin. Cells were plated on either 6-well plates or glass coverslips (Electron Microscopy Sciences, Fort Washington, PA, USA) pre-coated with 0.01% poly-L-lysine (Sigma-Aldrich) at the density of ~ 1 × 10^6^ and 1.0 × 10^4^ cells/well, respectively. After 5–6 h, the medium was replaced with fresh medium supplemented with 25 mM KCl. Cultures were used 5–7 days after plating and contained ~ 90% neurons as assessed by immunofluorescence staining with anti-neurofilament 160/200 (NF160/200) and anti-glial fibrillary acidic protein antibodies (Sigma-Aldrich). Experiments were performed according to the recommendations of the European Commission (EU Directive 2010/63/EU for animal experimentation) and were approved by the Institutional Ethical Committee.

### Cell treatment

Unless otherwise specified, neuroblastoma cells were washed with phosphate buffered saline (PBS) and incubated in medium containing 1% FCS, whereas cerebellar granule cells were washed and kept in Neurobasal-A medium without N-2 and the other supplements. Cells were treated with the test agents as indicated in the text, and maintained at 37 °C in a humidified atmosphere of 5% CO_2_ in air. Control samples received an equal amount of vehicle. To prepare cell lysates, cells were washed and scraped into ice-cold lysis buffer containing PBS, 0.1% sodium dodecyl sulphate (SDS), 1% Nonidet P-40, 0.5% sodium deoxycholate, 2 mM EDTA, 2 mM EGTA, 4 mM sodium pyrophosphate, 2 mM sodium orthovanadate, 10 mM sodium fluoride, 20 nM okadaic acid, 1 mM phenylmethylsulphonyl fluoride (PMSF), 0.5% phosphatase inhibitor cocktail 3 and 1% protease inhibitor cocktail (Sigma-Aldrich) (RIPA buffer). The samples were sonicated for 5 s in ice-bath and aliquots of cell extracts were taken for protein determination by the Bio-Rad protein assay (Bio-Rad Lab, Hercules, CA, USA).

### Transfection of small interfering RNA (siRNA)

SH-SY5Y cells were transfected with either 50 pmol/ml of either control siRNA-A (sc-37,007), human HDAC1 siRNA (sc-29,343), human CASZ1 siRNA (sc-78,956) or human p75NTR siRNA (sc-36,051) (Santa Cruz Biotechnology) duplexes using Lipofectamine RNAiMAX (Invitrogen-Thermo Fisher Scientific) as transfection reagent. Control siRNA consisted of a non-targeting sequence. Cells grown in 6-well plates were incubated in antibiotic-free medium for 24 h. The medium was renewed and the cells were incubated with siRNA duplexes for 4–5 h at 37 °C. Thereafter, the medium was replaced by the growth medium and the cells were analysed 48 h post-transfection. To determine transfection efficiency, parallel samples were transfected with fluorescein-conjugated control siRNA-A (sc-36,869, Santa Cruz Biotechnology). An efficiency of 55–65% was obtained in nine separate experiments.

### RNA extraction and quantitative polymerase chain reaction (qPCR)

SH-SY5Y and LAN-1 cells were incubated in a medium containing 1% FCS and treated for 24 h with either vehicle or VPA (1 mM). Thereafter, the cells were washed and total RNA was isolated by using TRIzol reagent (Invitrogen-Thermo Fisher Scientific) with the PureLink RNA mini kit (Ambion-Thermo Fisher Scientific). After RNA isolation, a Turbo DNase (Ambion-Thermo Fisher Scientific) digestion was performed. The purity and quantity of the RNA isolated were determined by UV absorbance at 260 and 280 nm. First-strand cDNA synthesis was performed using 2 µg of total RNA using SuperScript VILO cDNA synthesis kit (Invitrogen-Thermo Fisher Scientific). Two-hundred ng of cDNA for reaction was used for quantitative real time PCR amplification with SYBR Green PCR Master Mix (Applied Biosystems-Thermo Fisher Scientific). The PCRs were carried out on a Real-Time PCR System (StepOne, Applied Biosystems-Thermo Fisher Scientific) under the following conditions: an initial holding stage at 95 °C for 10 min was followed by 45 cycles: denaturation at 95 °C for 15 s, primer annealing and extension at 60 °C for 1 min and a dissociation curve to the end of a real time run (melt curve 95 °C for 15 s, 60 °C for 1 min and 95 °C for 15 s). PCR primers used were: human p75NTR forward CCTCATCCCTGTCTATTGCTCC, reverse GTTGGCTCCTTGCTTGTTCTGC; human sortilin forward CTGGGTTTGGCACAATCTTT, reverse CACCTTCCTCCTTGGTCAAA; human β-actin forward AGCCTCGCCTTTGCCGATCCG, reverse CATGCCGGAGCCGTTGTCGAC. The comparative Ct values method was used to calculate the relative quantity of p75NTR and sortilin expression.

### Biotinylation of surface proteins

Surface biotinylation of cell proteins was performed as previously described [35]. Briefly, SH-SY5Y and LAN-1 cells treated with either vehicle or VPA for 24 h were incubated for 45 min at 4 °C with the cell impermeable biotinylating agent sulfosuccinimidyl-6-(biotin-amido)hexanoate (sulpho-NHS-LC-biotin) (0.50 mg/ml) (Pierce, Rockford, IL, USA). Thereafter, the cells were washed with PBS containing 20 mM glycine and solubilized by incubation in RIPA buffer supplemented with 1% Triton X 100. Cell extracts were centrifuged at 10,000 x g for 5 min at 4 °C and the supernatants incubated overnight at 4 °C with streptavidin-conjugated agarose beads. Following washing the beads were mixed with sample buffer and incubated 2 min at 100 °C. The proteins were then analysed by Western blot.

### Immunoprecipitation

SH-SY5Y cells incubated for 24 h with either vehicle or VPA were treated with either vehicle or proNGF for 2 h. The cells were lysed with ice-cold RIPA buffer supplemented with 1% Triton X 100. Following centrifugation at 10,000 x g for 10 min at 4 °C, the supernatant (~ 500 µg of protein) was incubated overnight at 4 °C with either anti-p75NTR rabbit antibody (1:75) (Cell Signaling Technology, Danvers, MA, USA) or preimmune rabbit IgG (Santa Cruz Biotechnology). Thereafter, 50 µl of Pure Proteome Protein G magnetic beads (Millipore, Burlington, MA, USA) were added and samples incubated at 4 °C for 3 h with continuous rotation at 4 °C. The beads were washed 5 times with ice-cold PBS/0.1% Tween 20 buffer. After the last wash the pellet was resuspended in 2 x sample buffer and boiled for 5 min.

### Western blot analysis

Cell proteins were separated by SDS-polyacrylamide gel and electrophoretically transferred to polyvinylidene difluoride membranes (Millipore). Membranes were blocked, washed and incubated overnight at 4 °C with one of the following primary antibodies: p75NTR (cat. no. 8238) (1:1000), phospho-SAPK/JNK (Thr183/Tyr185) (cat. no. 9912) (1:1000), phospho-c-Jun (Ser73) (cat. no. 3270) (1:1000), c-Jun (cat. no. 9165) (1:1000), cleaved caspase 9 (Asp330) (cat. no. 7237) (1: 1000), caspase 9 (cat no. 9508) (1: 2000), cleaved caspase 3 (Asp175) (cat. no. 9664) (1:1000), caspase 3 (cat no. 9665) (1:1000), cleaved-poly(ADP-ribose) polymerase (PARP) (Asp214) (cat. no. 5625) (1:1000), PARP (cat. no. 9542) (1:1000), enhancer of zeste homolog 2 (EZH2) (cat. no. 5246) (1:1000), pan-cadherin (cat no. 4073) (1:2000) Cell Signaling Technology; sortilin (sc-376,561) (1:2000), CASZ1 (sc-398,303) (1:1000), HDAC1 (cat. no sc-81,598) (1:2000), JNK (sc-571) (1:2000) Santa Cruz Biotechnology; actin (cat no. A2066) (1:3000) and actin (cat. no. A5441) (1:20,000) Sigma-Aldrich. Thereafter, the membranes were washed and incubated with an appropriate horseradish peroxidase-conjugated secondary antibody (Santa Cruz Biotechnology). Immunoreactive bands were detected by using Clarity Western ECL substrate (Bio-Rad Laboratory, Hercules, CA, USA) and ECL Hyperfilm (Amersham, Piscataway, NJ, USA). The size of immunoreactive bands was determined by using molecular weight standards detected with an ECL suitable antibody (1:1000) (sc-2035, Santa Cruz Biotechnology). Band densities were determined using Image Scanner III (GE Healthcare, Milan, Italy) and NIH ImageJ software (US National Institutes of Health, Bethesda, MA, USA). The optical density of the phosphorylated protein bands was normalised to the density of the corresponding total protein in the same sample. For analysis of caspases and PARP, the formation of the cleaved protein was normalised to the level of the corresponding procaspase or uncleaved PARP measured in the same sample. For the remaining proteins, the densitometric values were normalised to the levels of either actin or subcellular fraction marker, as indicated.

### Cell viability and immunofluorescence analysis

For analysis of cell viability, cells grown onto poly-L-lysine-coated coverslips were exposed to the test agents as indicated in the text, incubated with 1 µg/ml propidium iodide (PI) (Sigma-Aldrich) for 1 h, washed and fixed in 4% paraformaldehyde. For analysis of phospho-c-Jun and cleaved-caspase 3 immunoreactivities, cells were fixed and permeabilised with 0.2% Triton X-100. Following blockade with 3% BSA and 1% normal goat serum, cells were incubated overnight at 4 °C with either rabbit anti-phospho-c-Jun (Ser73) (cat. no. 3270) (1:200) or rabbit anti-cleaved caspase-3 antibody (cat. no. 9661) (1:200) (Cell Signaling Technology). Mouse cerebellar granule cells were also stained with anti-neurofilament 160/200 (1:500). For analysis of p75NTR, non-permeabilised cells were blocked and incubated overnight with a rabbit antibody directed against an extracellular domain of p75NTR (cat. no. 8238, Cell Signaling Technology) (1:50). Control samples were incubated in the presence of rabbit pre-immune IgG. Cells were then incubated with an appropriate Alexa-Fluor488- or Alexa-Fluor594-conjugated secondary antibody (Invitrogen-Molecular Probes) and cell nuclei were stained with 0.1 µg/ml DAPI. Cells were analysed with an Olympus BX61 microscope equipped with a F-View II CCD-camera by using either a 40X or a 60X objective lens. Digital images were acquired using constant camera settings within each experiment and were analysed using the program Cell P (Olympus Soft Imaging Solutions, Homburg, Germany). At least 15 fields were randomly selected for each sample and only cells showing an unobstructed nucleus or soma were considered.

For quantitation of cell viability, PI positive cell nuclei were counted and the values expressed as percent of total nuclei. For quantitation of p75NTR, phospho-c-Jun and cleaved caspase 3 expression, the average pixel intensity was measured within the region of the cell soma or the nucleus and in an adjacent area, which was used as background value. Cells were deemed to be positive if the average pixel intensity was equal or above a threshold value corresponding to one standard deviation above the average pixel intensity of the respective control samples. No labelling was detected in samples treated with pre-immune IgG. For each experiment, four separate preparations of cells were analysed by an investigator unaware of the treatment.

### Statistical analysis

Results are reported as the mean ± SD of n independent experiments. Statistical analysis was performed by using the program Graph Pad Prism (San Diego, CA, USA). Unless otherwise indicated, data are expressed as percentage or fold stimulation of control, which was included in each independent experiment. The control group was set as 100 or 1 with a variance obtained by expressing each control value as a percentage of the mean of the raw values of the control group. In the experiments where control values were equal to zero, values of experimental groups were expressed as a percentage of the maximal effect set as 100. The variance of this value was determined in the same manner as for the control group. Statistical analysis was performed by using the nonparametric Mann-Whitney *U*-test. Statistical significance was set at *p* < 0.05.

## Results

### VPA upregulates p75NTR and sortilin expression in human neuroblastoma cells

**Fig. 1:**
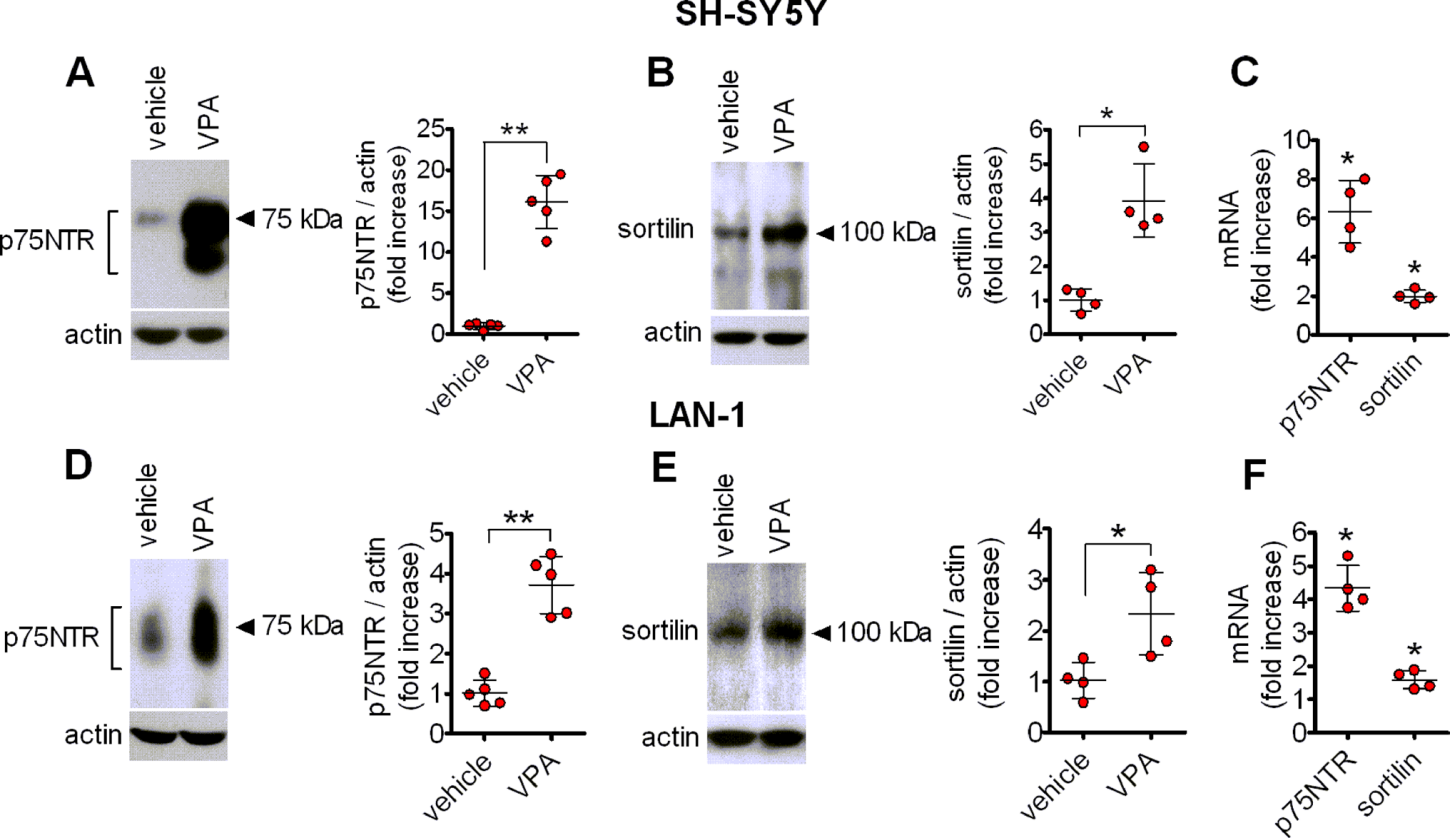
VPA upregulates p75NTR and sortilin expression in human neuroblastoma cells. SH-SY5Y and LAN-1 cells were treated for 24 h with either vehicle or 1 mM VPA and the expression of p75NTR (**a** and **d**) and sortilin (**b** and **e**) was analysed by Western blot and normalised to actin. Values are the mean ± SD of five (**a** and **d**) and four (**b** and **e**) independent experiments. **c** and **f** quantitative real-time RT-PCR analysis of p75NTR and sortilin mRNA levels in SH-SY5Y and LAN-1 cells, treated for 24 h with either vehicle or 1 mM VPA. Values are the mean ± SD of four independent determinations. *p < 0.05, **p < 0.01 vs. control (vehicle-treated cells)

Western blot analysis showed that prolonged exposure (24 h) of either SH-SY5Y or LAN-1 cells to VPA (1 mM) induced a marked increase in the expression of p75NTR immunoreactivity, which comprised a major band of 75 kDa and a faster migrating band likely corresponding to a lower glycosylation state of the receptor [36] (Fig. 1a and d). The upregulation of p75NTR induced by VPA was associated with an increase in the protein levels of the co-receptor sortilin (Fig. 1b and e). Moreover, real-time qRT-PCR analysis indicated that VPA (1 mM) significantly enhanced the steady state levels of p75NTR and sortilin mRNAs in both SH-SY5Y and LAN-1 cells (Fig. 1c and f).

### VPA increases the cell surface expression of p75NTR and sortilin

**Fig. 2:**
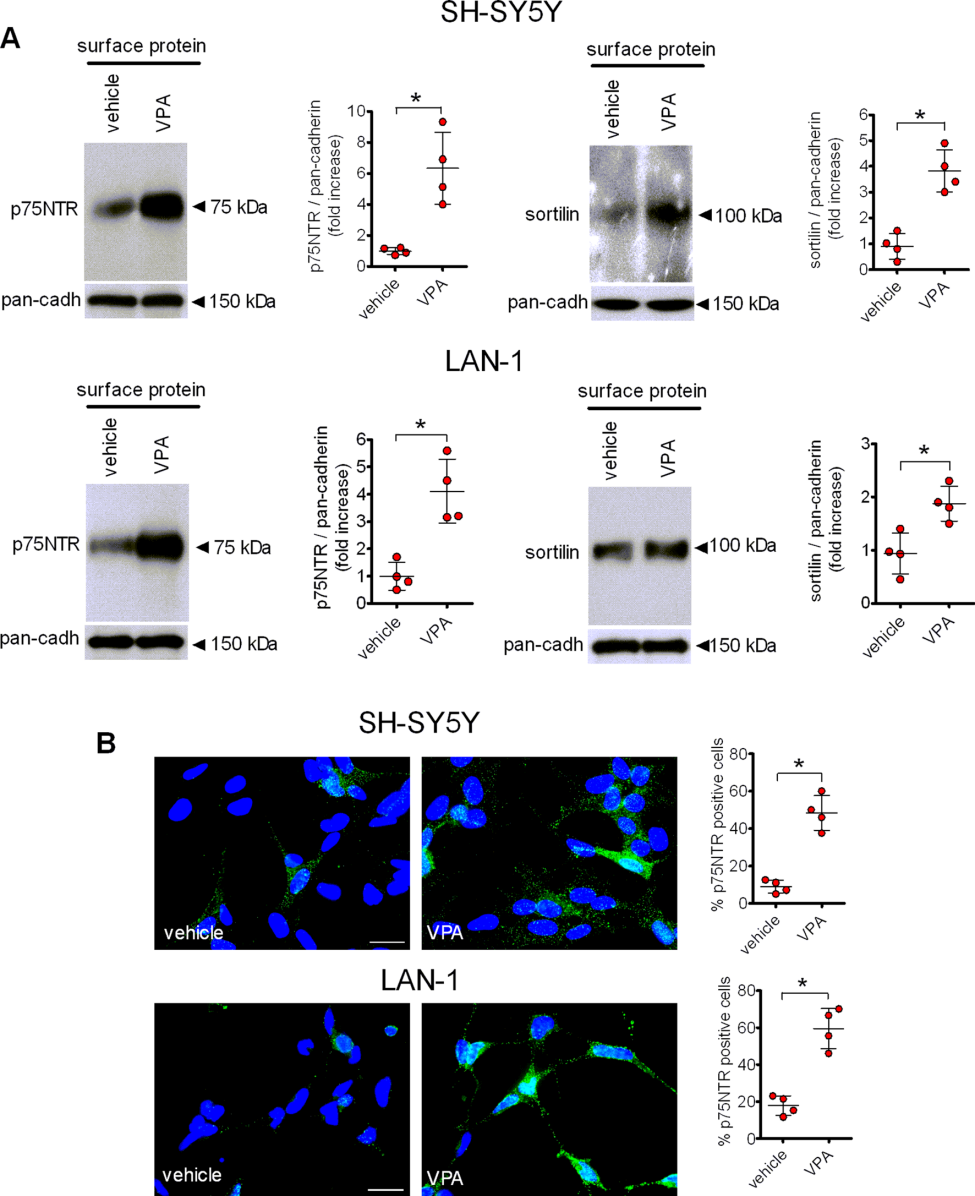
VPA enhances the plasma membrane expression of p75NTR and sortilin. **a** SH-SH5Y and LAN-1 cells were treated with either vehicle or VPA (1 mM) for 24 h and then exposed to the cell impermeant biotinylating agent sulpho-NHS-LC-biotin. The isolated surface proteins were analysed for p75NTR and sortilin by Western blot. The levels of p75NTR and sortilin in the cell surface protein preparation were normalised to the corresponding levels of pan-cadherin, a plasma membrane marker. Densitometric values are expressed as fold increase with respect to vehicle and are the mean ± SD of four independent experiments. **b** The expression of p75NTR was analysed by immunofluorescence (green color) in non-permeabilised SH-SY5Y and LAN-1 cells with an antibody directed against an extracellular domain of the receptor. Nuclei were stained in blue with DAPI. Values are the mean ± SD of four separate experiments. Bar = 25 µm. *p < 0.05 vs. control (vehicle) (Color figure online)

To examine whether the enhanced expression of p75NTR and sortilin elicited by VPA was accompanied by an increase in the plasma membrane levels of the two receptor proteins we performed cell surface protein biotinylation experiments and immunofluorescence analysis. As shown in Fig. 2a, treatment of plasma membrane proteins with the cell impermeable biotinylating agent sulpho-NHS-LC-biotin followed by precipitation with streptavidin-conjugated agarose beads revealed that exposure to VPA (1 mM) for 24 h significantly increased the cell surface levels of either p75NTR or sortilin in both SH-SY5Y and LAN-1 cells. Similarly, immunofluorescence analysis in non-permeabilised cells using a primary antibody directed against an extracellular epitope of p75NTR to preferentially label the receptor population present at the cell membrane showed that VPA (1 mM) increased the percent of cells displaying a positive immunoreactivity from the control value of 8.53 ± 3.4 to 45.6 ± 9.2% (*p* < 0.05) and from 18.3 ± 5.6 to 58.5 ± 11.6 (*p* < 0.05) in SH-SY5Y and LAN-1 cells, respectively (Fig. 2b).

### Time- and concentration-dependent induction of p75NTR and sortilin by VPA

**Fig. 3:**
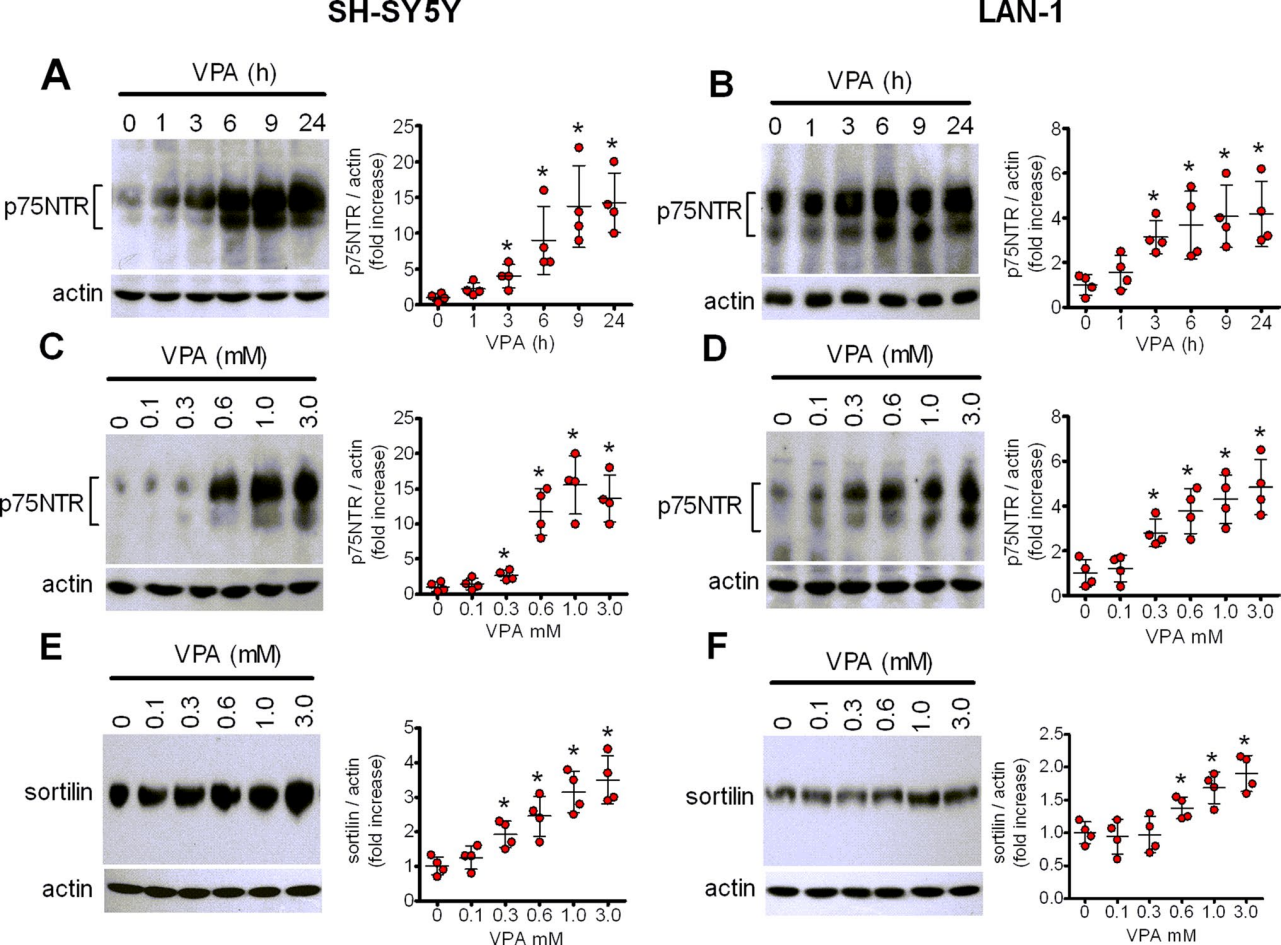
Time- and concentration-dependent upregulation of p75NTR and sortilin by VPA. **a**, **b**: SH-SY5Y and LAN-1 cells were incubated in the presence of 1 mM VPA for the indicated periods of time. Zero time samples were incubated with vehicle and used as control. **c**-**f**: SH-SY5Y (**c**, **e**) and LAN-1 (**d**, **f**) were incubated for 24 h in the presence of either vehicle or VPA at the indicated concentrations. Cell lysates were analysed for p75NTR and sortilin expression by Western blot. Values are the mean ± SD of four independent experiments. *p < 0.05 vs. control

Time-course experiments indicated that in SH-SY5Y cells p75NTR levels were significantly elevated (approximately 2-fold of basal value; *p* < 0.05) following 1 h treatment with VPA (1 mM), and then continued to increase reaching a maximum at 9–24 h (Fig. 3a). Similarly, in LAN-1 cells a significant increase of p75NTR was detected at 1 h of drug exposure and a maximal response, which lasted for at least 24 h, was reached at 6 h (Fig. 3b).

Analysis of concentration response curves, performed by incubating the cells for 24 h with VPA ranging from 0.1 to 3.0 mM, showed that a significant increase of p75NTR (twofold to threefold of basal value, *p* < 0.05) was detected at 0.3 mM VPA in either SH-SY5Y or LAN-1 cells, whereas the maximal increase was induced by approximately 1 and 3 mM VPA, respectively (Fig. 3c and d). Analysis of SH-SY5Y cell extracts for sortilin expression indicated a concentration-response curve qualitatively similar to that observed for p75NTR induction, with a significant enhancement observed following treatment with 0.3 mM VPA (Fig. 3e). In LAN-1 cells the lowest VPA concentration required to produce a significant increase of sortilin levels appeared to be approximately 0.6 mM, whereas the maximal response was observed at 1 mM VPA (Fig. 3f).

### HDAC inhibitors induce p75NTR and sortilin expression

**Fig. 4:**
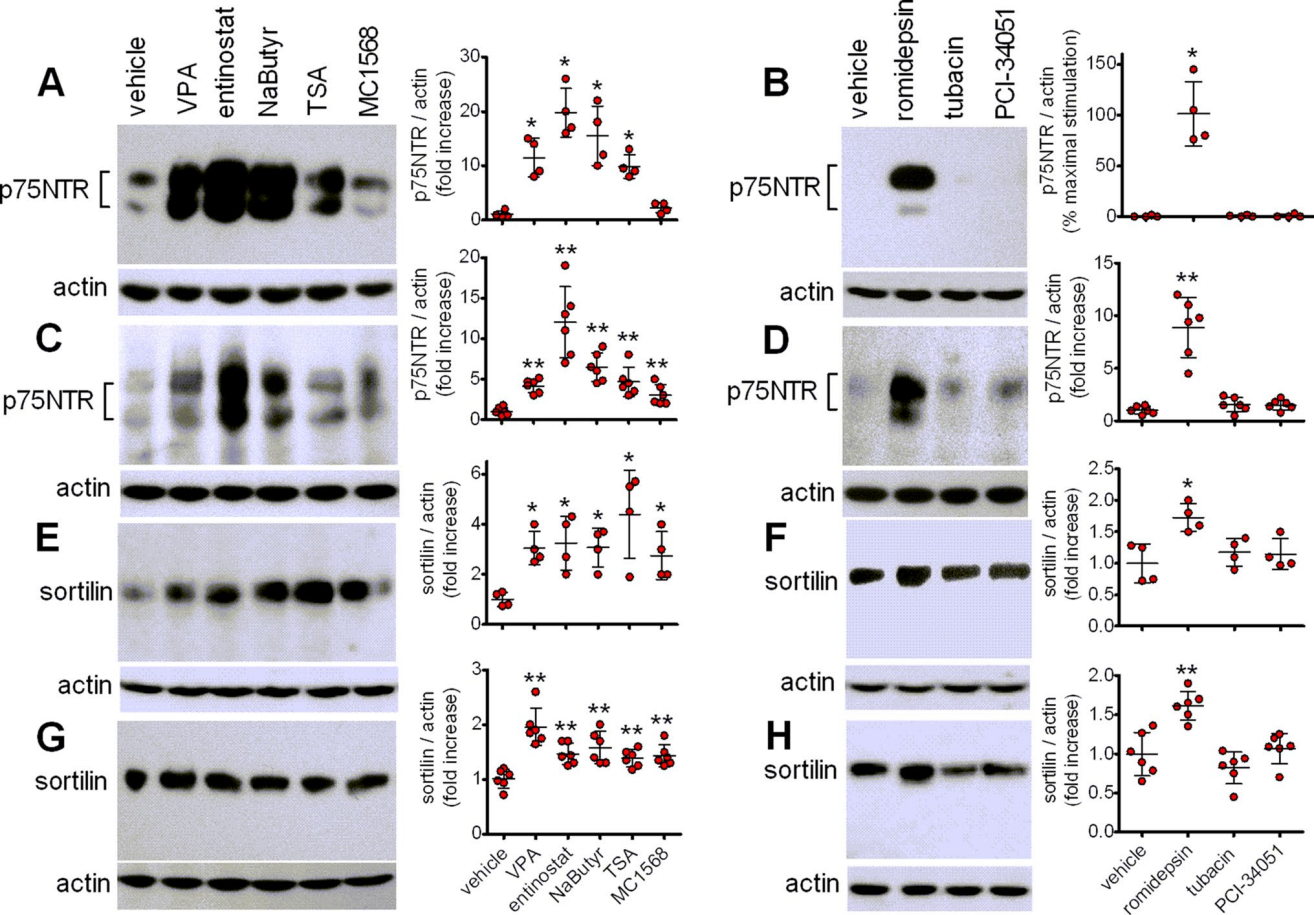
Effects of HDAC inhibitors on p75NTR and sortilin expression. SH-SY5Y (**a**, **b**, **e** and **f** ) and LAN-1 (**c**, **d**, **g** and **h**) cells were incubated for 24 h with either vehicle, 1 mM VPA, 1 µM entinostat, 1 mM sodium butyrate (NaButyr), 300 nM trichostatin A (TSA), 10 µM MC1568, 30 nM romidepsin, 5 µM tubacin, or 5 µM PCI-34,051. Cell lysates were analysed for p75NTR and sortilin expression. Values are the mean ± SD of four (**a**, **b**, **e** and **f**) and six (**c**, **d**, **g** and **h**) independent experiments. *p < 0.05, **p < 0.01 vs. control (vehicle-treated cells)

In addition to VPA, other HDAC inhibitors were able to induce p75NTR and sortilin expression. In both SH-SY5Y and LAN-1 cells prolonged exposure (24 h) to entinostat (1 µM ), a class I HDAC inhibitor, romidepsin (30 nM), a selective inhibitor of the class I HDAC1 and 2, the short-chain fatty acid sodium butyrate (1 mM), which, like VPA, preferentially inhibits class I and IIa HDAC, and the broad spectrum HDAC inhibitor trichostatin A (300 nM) [9], caused a marked induction of p75NTR expression (Fig. 4a-d). Conversely, the class II HDAC inhibitor MC1568 (10 µM) [37] was without significant effects in SH-SY5Y cells and elicited a modest stimulatory response in LAN-1 cells (Fig. 4a and c). The HDAC6 inhibitor tubacin (5 µM) [38] and the HDAC8 inhibitor PCI-34,051 (5 µM) [39] had no effect in both cell lines (Fig. 4b and d).

We next examined the effect of the distinct HDAC inhibitors on sortilin expression. Cell treatment with entinostat, sodium butyrate, trichostatin A, MC1568 and romidepsin significantly increased the cellular levels of sortilin, whereas tubacin and PCI-34,051 were without effect (Fig. 4e-h).

### ***VPA*** - induced p75NTR overexpression is mimicked by HDAC1 knockdown and involves opposite changes in the transcriptional regulators EZH2 and CASZ1

**Fig. 5:**
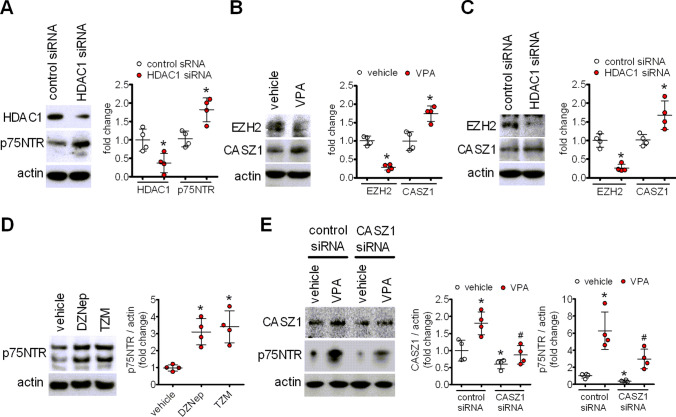
VPA-induced p75NTR upregulation is mimicked by HDAC1 knockdown and involves CASZ1 derepression. **a** SH-SY5Y cells were transfected with either control siRNA or HDAC1 siRNA duplexes and cell lysates were analysed for HDAC1 and p75NTR expression 48 h post-transfection. Values are the mean ± SD of four separate experiments. *p < 0.05 vs. the corresponding sample treated with control siRNA. **b**, **c** Cells treated for 24 h with either vehicle or 1 mM VPA (**b**) or transfected with either control or HDAC1 siRNAs (**c**) were analysed for the levels of EZH2 and CASZ1 by Western blot. Values are the mean ± SD of four separate experiments. *p < 0.05 vs. control (vehicle) or the corresponding sample treated with control siRNA. **d** Cells were treated for 72 h with either vehicle, deazaneplanocin (**a**) (DZNep) (1 µM) or tazemetostat (TZM) (1 µM). The levels of p75NTR were measured by Western blot and normalised to actin. Values are the mean ± SD of four independent experiments. *p < 0.05 vs. vehicle. **e** Cells were transfected with either control siRNA or CASZ1 siRNA and 45 h post-transfection treated with either vehicle or 1 mM VPA for 24 h. Cell lysates were analysed for the expression of CASZ1 and p75NTR by Western blot. Values are the mean ± SD of four experiments. *p < 0.05 vs. vehicle in control siRNA-treated cells; ^#^*p* < 0.05 vs. VPA in control siRNA-treated cells

The results obtained with the different HDAC inhibitors indicated that blockade of class I HDACs, rather than class II HDACs, was likely involved in the upregulation of p75NTR. We further investigated this possibility by knocking down HDAC1, a widely expressed member of class I HDACs that is inhibited by therapeutically relevant concentrations of VPA [7]. Treatment of SH-SY5Y cells with siRNA duplexes targeting HDAC1 reduced the protein levels of HDAC1 by 68 ± 26% (*p* < 0.05) and increased p75NTR expression by 75 ± 20% (*p* < 0.05), as compared to control siRNA-treated cells (Fig. 5a).

Previous studies by Wang et al. [40] have shown that in human neuroblastoma cells romidepsin upregulated p75NTR mRNA by inducing the depletion of the histone methyltransferase EZH2, the catalytic subunit of the polycomb repressive complex 2 (PRC2), with the consequent derepression of the transcription factor CASZ1, which positively regulates p75NTR transcription by binding to the NGFR promoter [41]. We therefore examined whether a similar epigenetic mechanism was involved in p75NTR induction by VPA. As shown in Fig. 5b and c, in SH-SY5Y cells either exposure to VPA (1 mM) or HDAC1 knockdown significantly decreased EZH2 protein levels and upregulated CASZ1 expression. Moreover, both 3-deazaneplanocin A (DZNep) (1 µM), an inhibitor of S-adenosylhomocysteine hydrolase which causes EZH2 depletion [42], and tazemetostat (TZM) (1 µM), a direct EZH2 inhibitor [43], induced p75NTR expression (Fig. 5d). To gain additional evidence for a role of CASZ1 in VPA-induced p75NTR upregulation, SH-SY5Y cells were treated with CASZ1 siRNA duplexes. As shown in Fig. 5e, this treatment blocked the VPA-induced increase of CASZ1 and significantly attenuated the induction of p75NTR elicited by the drug. Collectively, these results indicate that EZH2 depletion and the consequent CASZ1 derepression participate in the upregulation of p75NTR induced by VPA.

### Exposure to VPA enhances proNGF-induced formation of p75NTR/sortilin complex and activation of JNK

**Fig. 6:**
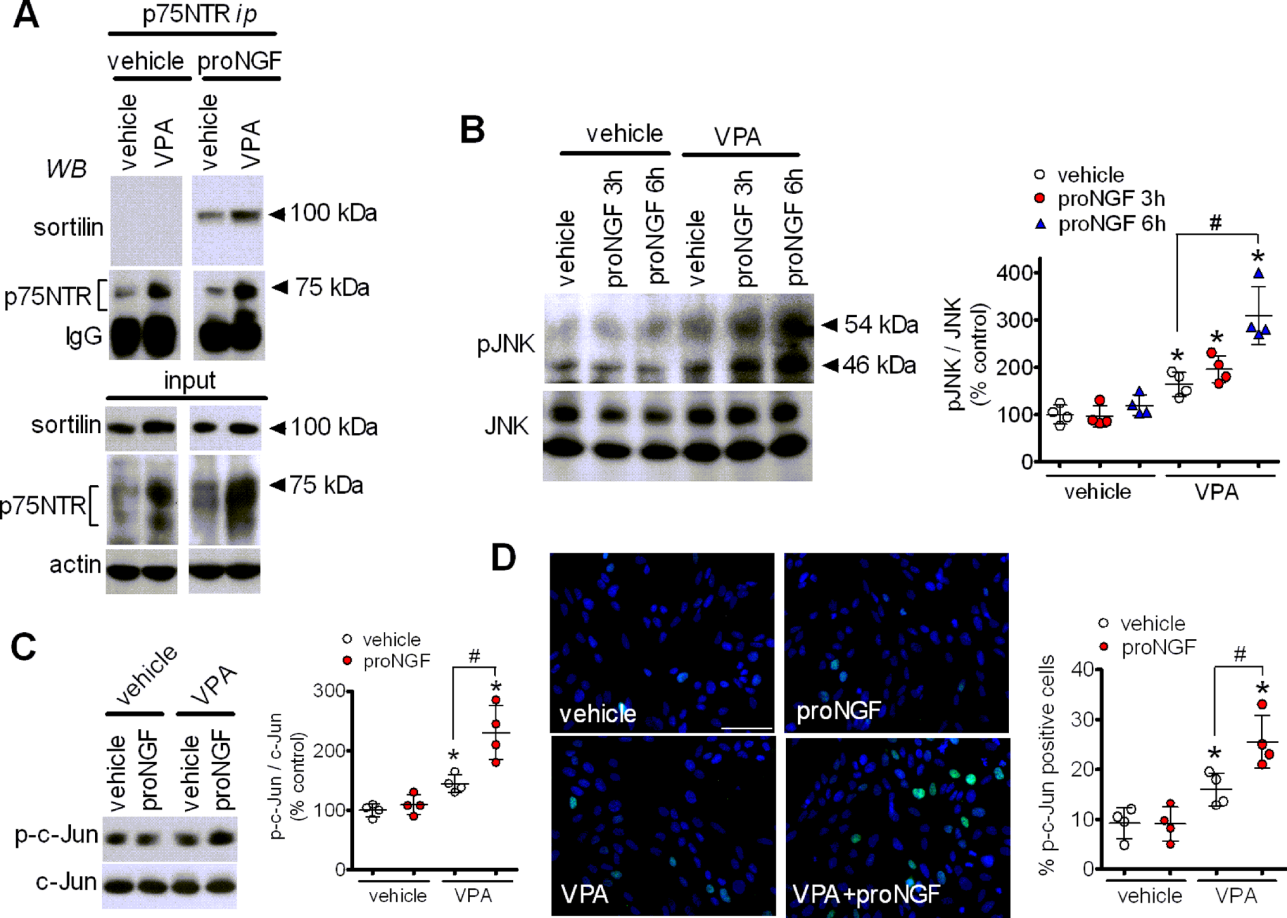
Exposure to VPA promotes proNGF-induced p75NTR/sortilin interaction and JNK activation. **a** SH-SY5Y cells were incubated for 24 h with either vehicle or 1 mM VPA and then treated for 2 h with either vehicle or proNGF (1 µg/ml). Thereafter, cell lysates were subjected to immunoprecipitation with an anti-p75NTR antibody. Immunoprecipitates and cell lysates (input) were analysed for sortilin and p75NTR immunoreactivities. The immunoblot is representative of three independent experiments. **b** SH-SY5Y cells were incubated for 24 h with either vehicle or 1 mM VPA and then exposed to either vehicle or proNGF (5 ng/ml) for 3 and 6 h. Cell lysates were analysed for phospho-JNK (pJNK) and JNK levels. Values are the mean ± SD of four independent experiments. *p < 0.05 vs. control (vehicle + vehicle); ^#^*p* < 0.05. **c** SH-SY5Y cells were incubated for 24 h with either vehicle or 1 mM VPA and then treated for 6 h with either vehicle or 5 ng/ml proNGF. Cell lysates were analysed for the expression of phospho-c-Jun (p-c-Jun) and c-Jun. **d** SH-SY5Y cells were treated as indicated in C and analysed for phospho-c-Jun expression (green color) by immunofluorescence microscopy. Cell nuclei were stained in blue with DAPI. Values indicate the percent of phospho-c-Jun positive cells and are the mean ± SD of four independent experiments. Bar = 50 µm. *p < 0.05 vs. control (vehicle + vehicle); ^#^*p* < 0.05 (Color figure online)

We next examined the functional outcome of the p75NTR and sortilin induction by VPA. To this goal, we first investigated whether VPA treatment enhanced the interaction between p75NTR and sortilin. As this interaction has been shown to be strengthened by the formation of a trimeric complex with proneurotrophins [32, 44], SH-SY5Y cells were preincubated for 24 h with or without 1 mM VPA and then treated for 2 h with either vehicle or proNGF (1 µg/ml). To avoid the conversion to NGF, a cleavage-resistant mutated form of proNGF was used. Following treatment, cell lysates were subjected to immunoprecipitation with either an anti-p75NTR or a preimmune antibody and the immunoprecipitates were analysed for the presence of p75NTR and sortilin by Western blot. Incubation of cell lysates with preimmune antibody failed to immunoprecipitate either sortilin or p75NTR under each experimental condition (results not shown), whereas, as expected, the anti-p75NTR antibody immunoprecipitated a greater amount of p75NTR from lysates of cells preincubated with VPA. In agreement with previous studies [32], co-immunoprecipitation of sortilin was only observed following treatment with proNGF, indicating the formation of a heterotrimeric complex including proNGF, sortilin and p75NTR (Fig. 6a). In cells preexposed to VPA, the amount of sortilin co-immunoprecipitated with p75NTR increased markedly, indicating that the upregulation of p75NTR and sortilin induced by the drug was accompanied by an increased formation of the receptor complex in response to proNGF.

Activation of JNK through the NRAGE adaptor protein is considered as a main cell death signalling mechanism triggered by p75NTR [45]. As shown in Fig. 6b, treatment of SH-SY5Y cells with proNGF (5 ng/ml) for either 3 or 6 h failed to induce a significant increase in the phosphorylation/activation state of JNK. However, a significant stimulation of JNK phosphorylation by proNGF was observed at both time points in cells pretreated for 24 h with VPA, which per se caused a modest stimulatory effect. In line with these results, in cells pretreated with vehicle proNGF (5 ng/ml) had no effect on the levels of the transcription factor c-Jun phosphorylated at Ser73, a JNK phosphorylation site [46], but significantly increased the levels of phosphoSer73-c-Jun in cells preincubated with VPA (Fig. 6c). Similar results were obtained by immunofluorescence analysis which showed that pretreatment with VPA increased the percent of SH-SY5Y cells positive for phosphoSer73-c-Jun immunoreactivity and that this response was potentiated by a subsequent exposure to proNGF (Fig. 6d).

### Cell treatment with VPA induces neuroblastoma cells apoptosis which is potentiated by proNGF

**Fig. 7:**
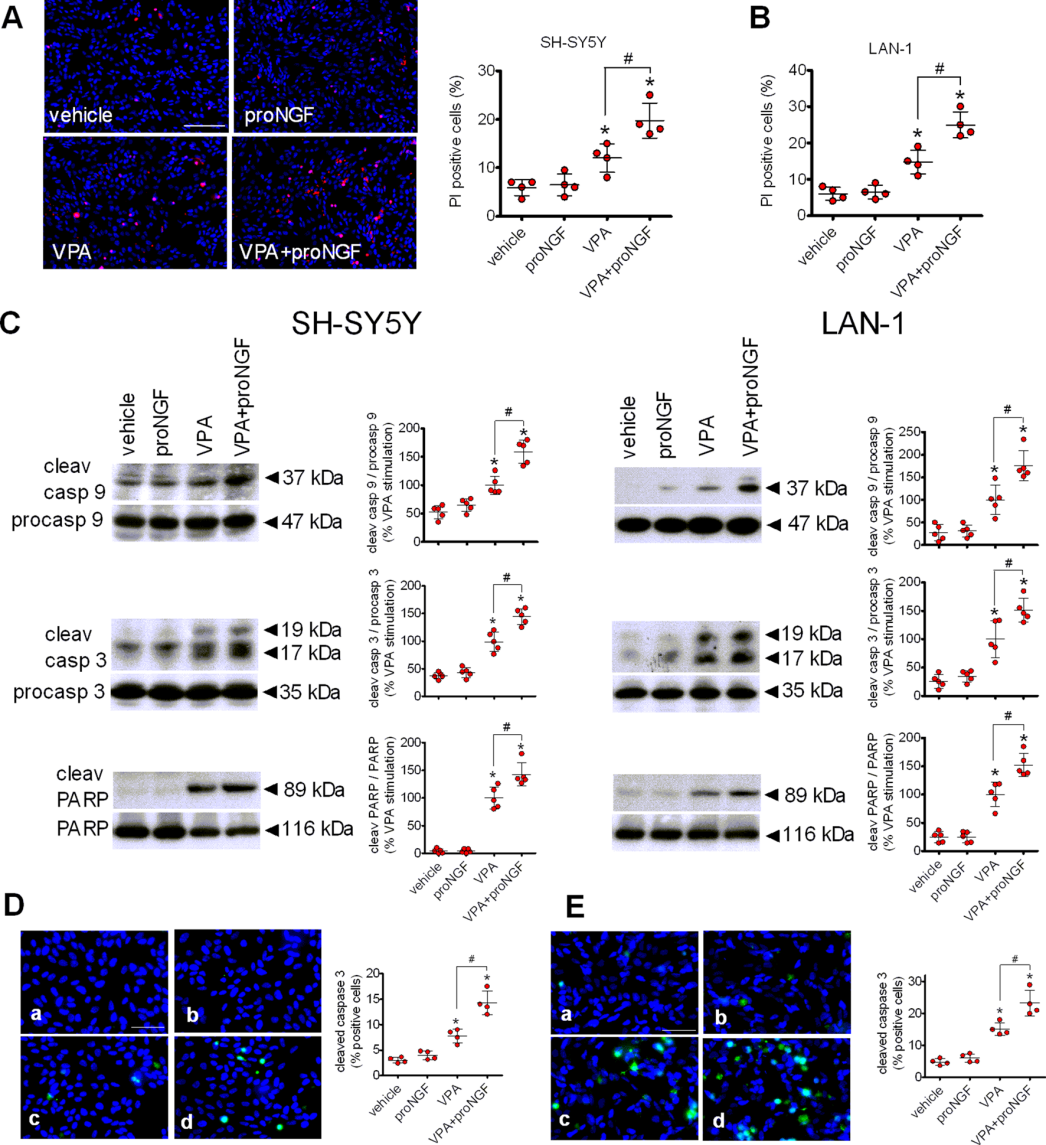
Cell treatment with proNGF potentiates VPA-induced apoptosis. **A** SH-SY5Y cells grown onto glass coverslips were incubated for 24 h with either vehicle or 1 mM VPA and then exposed for additional 24 h to either vehicle or 5 ng/ml proNGF. Dead cells were identified by propidium iodide fluorescence (red color), whereas cell nuclei were stained with DAPI (blue color). Values are the mean ± SD of four experiments. Bar = 100 µm. **B** LAN-1 cells were grown, treated and analysed as indicated in A. **C** SH-SY5Y and LAN-1 cells were incubated for 24 h with either vehicle or 1 mM VPA and then exposed for 24 h to either vehicle or 5 ng/ml proNGF. Cell lysates were analysed for cleaved caspase (cleav casp) 9, procaspase (procasp) 9, cleaved caspase 3, procaspase 3, cleaved PARP and total PARP. Values are the mean ± SD of five independent experiments. **D**, **E** SH-SY5Y (D) and LAN-1 (E) cells grown onto glass coverslips were treated as indicated in C and then processed for cleaved caspase 3 immunofluorescence (green color) analysis. a = vehicle; b = proNGF; c = VPA; d = VPA + proNGF. Positive cells are expressed as percent of total cells. Values are the mean ± SD of four independent experiments. Bar = 50 µm. *p < 0.05 vs. vehicle. ^#^*p* < 0.05 (Color figure online)

We next examined whether the upregulation of p75NTR and sortilin by VPA was associated with a change in neuroblastoma cell viability and whether this response was affected by proNGF. As shown in Fig. 7A staining with propidium iodide to mark dead cells indicated that VPA treatment for 24 h caused a significant decrease in the viability of SH-SY5Y cells. Exposure to proNGF (5 ng/ml) for 24 h had no effect on cell viability of vehicle-pretreated cells, but significantly enhanced the cell death in VPA-pretreated cells. Similar results were obtained in LAN-1 cells (Fig. 7B).

In both SH-SY5Y and LAN-1 cells VPA-induced neuroblastoma cell death was associated with an increased formation of active cleaved caspases 9 and 3 and stimulation of PARP cleavage at a caspase-sensitive site (Fig. 7C). Exposure to proNGF had no effect on caspase activation and PARP cleavage in vehicle-pretreated cells but significantly enhanced these apoptotic events in cells preexposed to VPA. Similar results were obtained when the expression of cleaved caspase 3 was examined by immunofluorescence. VPA significantly increased the percent of cleaved caspase 3 positive cells in both neuroblastoma cell lines (Fig. 7D and E). The subsequent treatment with proNGF augmented the percent of apoptotic cells in VPA- but not vehicle-pretreated cultures.

**Fig. 8:**
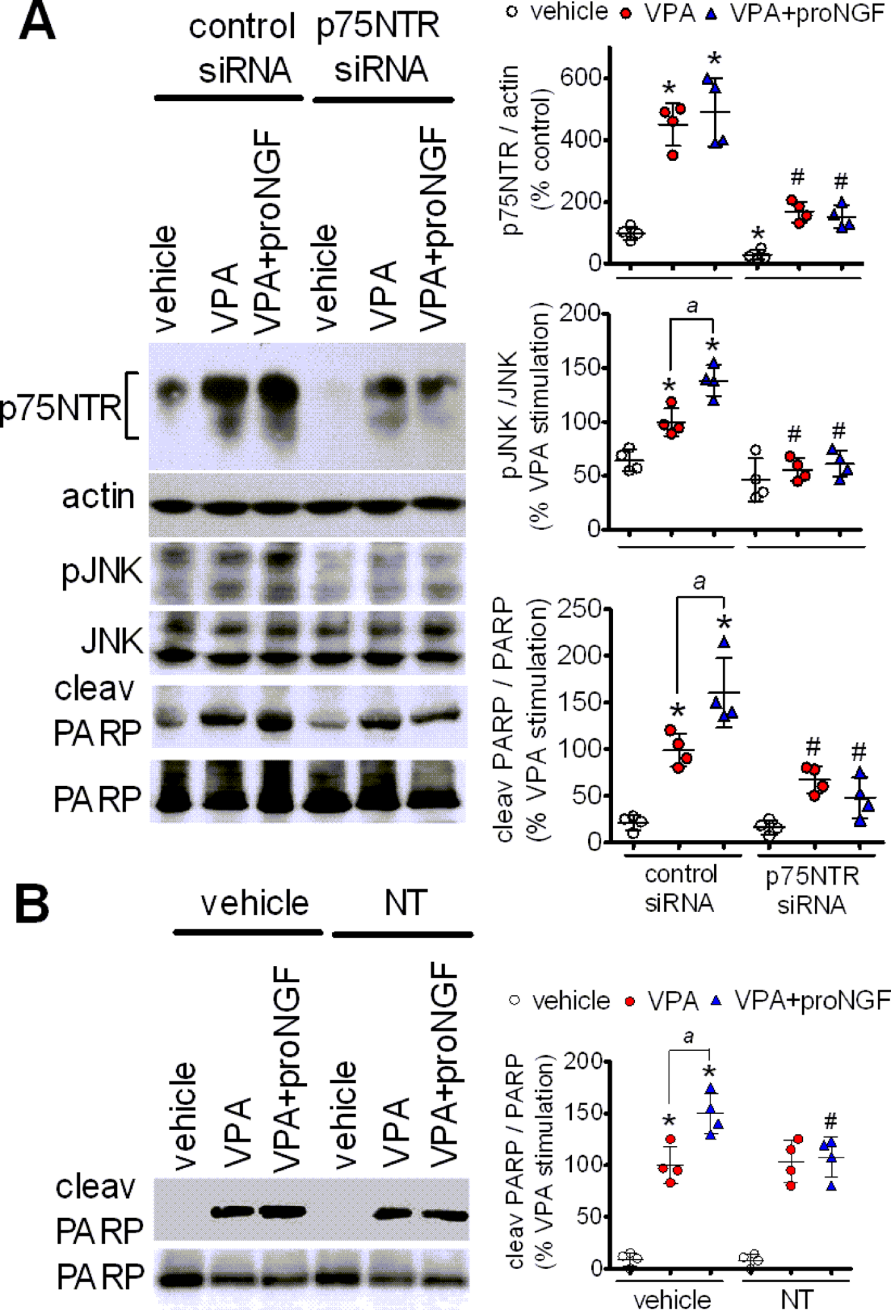
Involvement of p75NTR/sortilin receptor complex in the apoptosis induced by VPA and pro-NGF. **a** SH-SY5Y cells were transfected with either control siRNA or p75NTR siRNA duplexes. Forty-eight h post-transfection cells were incubated for 24 h with either vehicle or 1 mM VPA and then exposed to either vehicle or 5 ng/ml proNGF for additional 24 h. Cell lysates were analysed for p75NTR, phospho-JNK and cleaved PARP expression. Values are the mean ± SD of four independent experiments. *p < 0.05 vs. vehicle in control siRNA-treated cells. ^a^ *p* < 0.05; ^#^*p* < 0.05 vs. the corresponding sample in control siRNA-treated cells. **b** SH-SY5Y cells were pretreated for 24 with either vehicle or 1 mM VPA. Thereafter, the cells were first exposed to either vehicle or 5 µM neurotensin (NT) for 2 h and then to vehicle or 5 ng/ml proNGF for additional 22 h. Cell lysates were analysed for cleaved and uncleaved PARP. Densitometric ratios are expressed as percent of PARP cleavage induced by VPA in the absence of NT and are the mean ± SD of four independent experiments. *p < 0.05 vs. control (vehicle + vehicle). ^a^ *p* < 0.05; ^#^*p* < 0.05 vs. the corresponding sample in vehicle-treated cells

We then investigated whether VPA-induced apoptosis and its potentiation by proNGF were dependent on the upregulation of the p75NTR/sortilin receptor complex. To assess the p75NTR-dependence, SH-SY5Y cells were treated with p75NTR siRNA and then sequentially exposed to VPA and proNGF. As shown in Fig. 8a, p75NTR siRNA treatment decreased the basal expression of p75NTR and greatly attenuated the induction by VPA irrespective of subsequent treatment with proNGF. Depletion of p75NTR attenuated VPA-induced JNK phosphorylation and apoptosis, as indicated by the reduced formation of cleaved PARP, and completely prevented the potentiation of these responses elicited by proNGF (Fig. 8a).

To investigate the involvement of sortilin, cells were pretreated with neurotensin (NT) (5 µM), which has been shown to antagonize the binding of proNGF to sortilin [30]. As shown in Fig. 8b, the addition of NT did not affect VPA-induced PARP cleavage but completely prevented the potentiation induced by proNGF.

### VPA enhances p75NTR and sortilin expression and promotes proNGF-induced apoptosis in mouse cerebellar granule cells

**Fig. 9:**
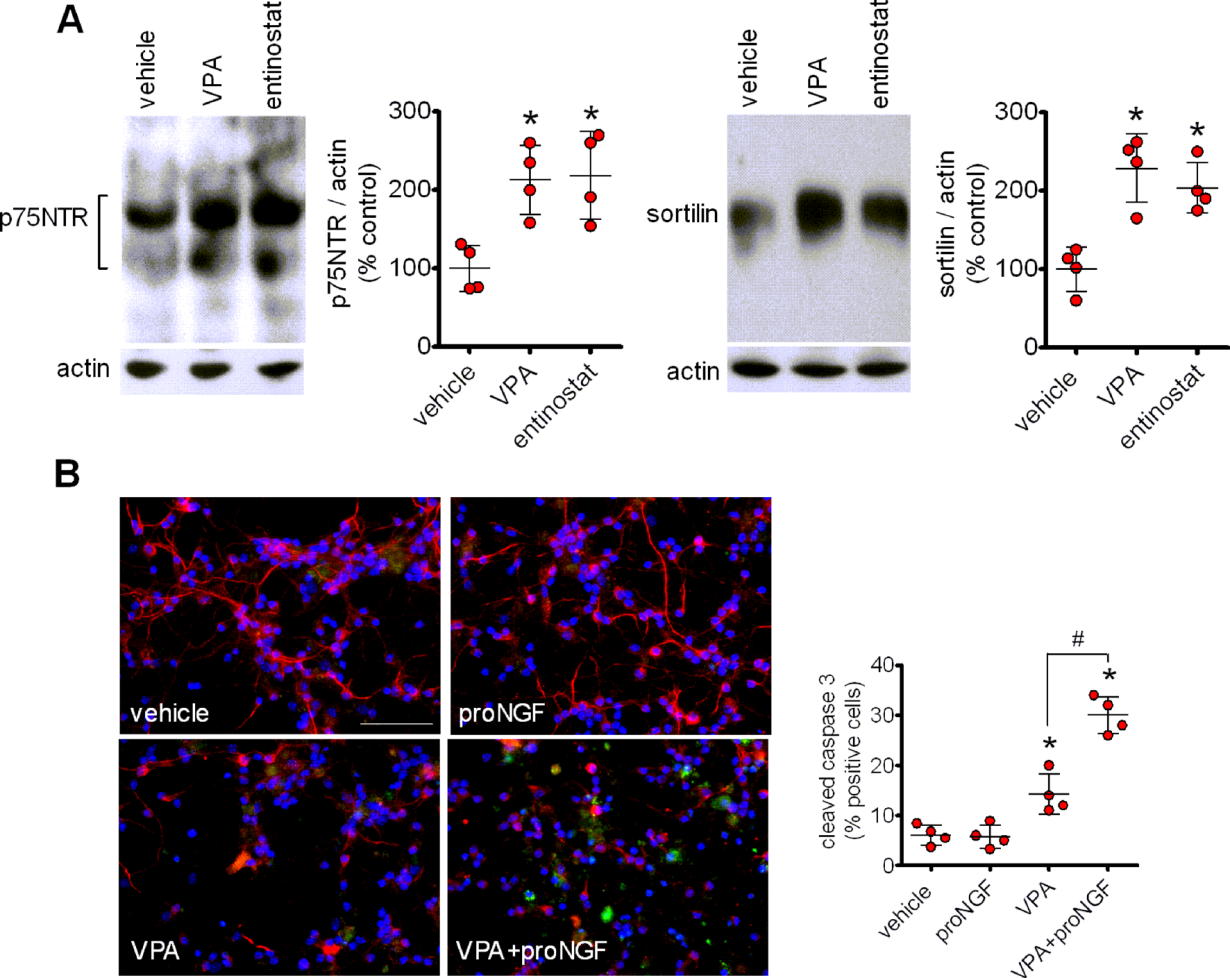
VPA upregulates p75NTR and sortilin expression and promotes proNGF-induced apoptosis in mouse cerebellar granule cells.** a** cells were incubated for 24 h with either vehicle, 1 mM VPA or 1 µM entinostat and cell lysates were analysed for p75NTR and sortilin expression. Values are the mean ± SD of four independent experiments.** b** cells were incubated for 24 h with either vehicle or 1 mM VPA and then exposed for additional 24 h to either vehicle or 5 ng/ml proNGF. Cells were analysed for cleaved caspase 3 (green color) and neurofilament 160/200 (red color) by immunofluorescence microscopy. Nuclei were stained with DAPI (blue color). Bar = 50 µm. Values are the mean ± SD of four experiments. *p < 0.05 vs. vehicle. ^#^*p* < 0.05 (Color figure online)

Prolonged exposure (24 h) of primary cultures of mouse cerebellar granule cells to VPA (1 mM) induced a significant increase in the expression of p75NTR and sortilin (Fig. 9a). Similar effects were obtained by treating the cells with entinostat (1 µM). Treatment of primary cultures with VPA increased the percent of apoptotic cells, as indicated by the immunofluorescence analysis of cleaved caspase 3 expression (Fig. 9b). Exposure to proNGF (5 ng/ml) had no effect on cells pretreated with vehicle, but significantly increased the percent of apoptotic cells in VPA-pretreated samples.

## Discussion

Proneurotrophin-induced activation of the p75NTR receptor is considered to act as a key regulator of neuronal death in the developing and aging brain, as well as in different neurodegenerative diseases [25, 30, 47, 48]. The present study shows that prolonged exposure of human neuroblastoma cells and mouse cerebellar granule cells to VPA upregulates p75NTR and sortilin expression, triggers apoptosis and predisposes to proNGF-enhanced cell death, thus disclosing a novel mechanism whereby VPA adversely affects neuronal survival.

In both SH-SY5Y and LAN-1 cells VPA-induced upregulation of p75NTR and sortilin was associated with an enhanced expression of the receptor proteins at the plasma membrane. This localization is critical for the receptor function. For instance, in PC12 rat pheochromocytoma cells the magnitude of the responses to NGF has been reported to depend on the level of p75NTR cell surface expression [49, 50]. Sortilin is predominantly localized in intracellular compartments [51] and increases in its plasma membrane expression, in conjunction with p75NTR, have been found to be associated with enhanced proneurotrophin-induced neuronal cell death [52, 53]. Thus, the ability of VPA to enhance the cell surface levels of both p75NTR and sortilin provides the condition for the formation of a receptor complex capable of detecting extracellular stimuli and propagating biologically relevant intracellular signals.

We found that in human neuroblastoma cells the upregulation of p75NTR and sortilin occurred at VPA concentrations ranging from 0.3 to 1.0 mM. These values are higher than the free plasma concentrations of VPA detected in patients treated with antiepileptic doses (0.034–0.104 mM) [54], but are consistent with the free plasma concentrations reached following administration of anti-tumour doses of the drug, which are much higher than those used in neuropsychiatric diseases [3, 55, 56]. For instance, in a phase I/II clinical trial evaluating VPA potentiation of epirubicin effects in advanced solid tumours, patients receiving 120 mg/kg/day VPA showed mean total and free plasma concentrations of 1.52 and 0.75 mM, respectively [56]. Thus, the VPA-induced changes observed in the present study may have clinical relevance when the drug is used to treat extra-cranial tumours, such as neuroblastoma. Moreover, within the same concentration range VPA was previously found to cause hyperacetylation of H3 histone and downregulation of TrkB in SH-SY5Y cells [57], indicating that the drug can induce chromatin remodelling and altered expression of neurotrophin receptors with similar potencies.

**Fig. 10:**
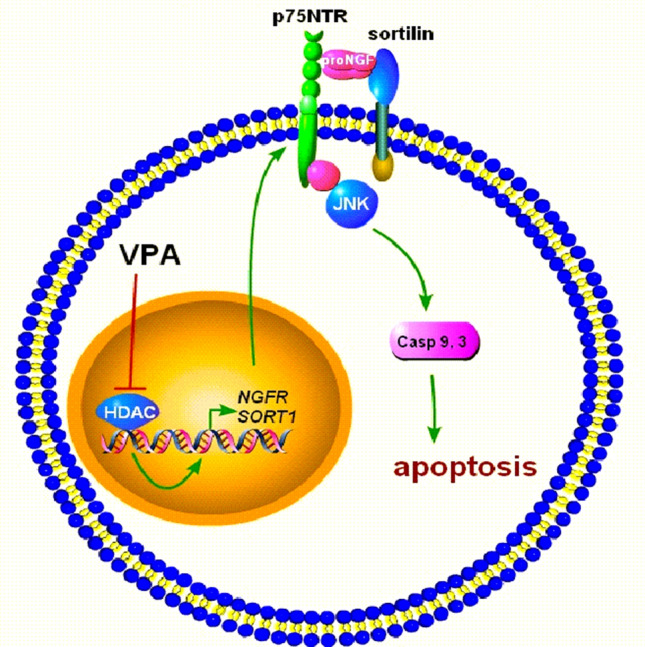
Schematic diagram illustrating the induction of p75NTR and sortilin by VPA and the consequent promotion of proNGF-induced neuronal apoptosis. The inhibition of HDACs is shown to induce the transcription of NGFR and SORT1 genes, leading to an enhanced p75NTR and sortilin expression at the plasma membrane. These receptor changes sensitise neuronal cells to the proapototic action of proNGF through the activation of JNK signalling

The upregulation of p75NTR and sortilin induced by VPA was associated with increased levels of the respective mRNAs, suggesting that these changes result from an action of the drug on gene transcription. Moreover, the finding that other HDAC inhibitors were able to induce p75NTR and sortilin expression points to the participation of an epigenetic mechanism whereby HDAC blockade positively affects the activity of NGFR and SORT1 genes encoding p75NTR and sortilin, respectively (Fig. 10). With regard to NGFR, the present study provides evidence that one mechanism by which VPA upregulates p75NTR expression involves the depletion of the PRC2 core component EZH2 and the consequent derepression of CASZ1, a neuroblastoma tumour suppressor and a positive regulator of NGFR [40, 41]. As previously observed with other HDAC inhibitors, which suppress PRC2 likely through proteasome-mediated degradation [42, 58], treatment of SH-SY5Y cells with VPA induced a significant decrease in EZH2 expression levels. This effect was accompanied by an increase of CASZ1 levels, and blockade of this response by CASZ1 siRNA transfection prevented VPA-induced p75NTR upregulation. Like VPA, HDAC1 knockdown enhanced p75NTR, inhibited EZH2 and increased CASZ1 expression. Moreover, cell treatment with the EZH2 depleting agent DZNep or the EZH2 inhibitor TZM upregulated p75NTR. Interestingly, VPA-induced downregulation of TrkB was recently found to involve depletion of EZH2 and derepression of RUNX3 [57], a negative regulator of the NTRK2 gene. Thus, a common epigenetic mechanism based on inhibition of PRC2 transcriptional repression appears to mediate the VPA opposite effects on p75NTR and TrkB expression.

Relatively little is known on the control of SORT1 gene expression and the precise molecular mechanisms linking HDAC inhibition and sortilin upregulation remain to be elucidated. Nonetheless, the observation that the HDAC6 inhibitor tubacin and the HDAC8 inhibitor PCI-34,051, which do not induce histone hyperacetylation [38, 39, 57], had no effects on either sortilin or p75NTR expression indicates that both responses are not induced by generic HDAC inhibition but involve specific HDAC isoforms capable of producing chromatin remodelling.

Immunoprecipitation experiments indicated that in VPA-treated SH-SY5Y cells the upregulation of p75NTR and sortilin expression was accompanied by an enhancement of proNGF-induced association of the two receptor proteins and potentiation of JNK activation, as indicated by the increased phosphorylation of JNK and c-Jun. In control cells proNGF was unable to induce JNK activation, possibly because of the lower amount of p75NTR and sortilin available for the formation of a receptor complex at sufficient levels.

Previous studies have provided evidence that activation of JNK, which follows the binding of NRAGE to the cytosolic domain of p75NTR, causes cell death through the mitochondrial apoptotic pathway initiated by the release of cytocrome c and activation of caspase 9 [45]. We found that in both SH-SY5Y and LAN-1 cells prolonged exposure to VPA induced a significant increase in the number of dead cells which was associated with enhanced activation of caspases 9 and 3 and caspase-dependent PARP cleavage. The cell death and the activation of the intrinsic apoptotic cascade induced by VPA was potentiated by the subsequent exposure to proNGF. In vehicle-pretreated cells, expressing basal levels of p75NTR and sortilin, proNGF failed to affect the cell viability and the expression of apoptotic effectors, consistent with the lack of effect on JNK activity. In VPA-treated cells the attenuation of p75NTR upregulation by siRNA treatment prevented proNGF-induced JNK activation and PARP cleavage, indicating that these responses were dependent on the enhanced expression of p75NTR. The finding that the depletion of p75NTR reduced the activation of JNK and PARP cleavage also in VPA-treated cells suggests that the upregulation of p75NTR triggered death signalling in the absence of proNGF. This result is consistent with previous studies showing that ectopic overexpression of p75NTR causes ligand independent apoptotic cell death of human neuroblastoma cells [59].

The upregulation of p75NTR and sortilin expression may have important implications for the anti-tumour activity of VPA. Clinical studies in primary neuroblastic tumours have shown that p75NTR mRNA expression correlated with increased event-free and overall survival and suggested that induction of p75NTR could be a strategy to reduce tumourigenicity of neuroblastoma [60]. In this context, the enhanced sensitivity to the proapoptotic action of proNGF may play a role, as proNGF is produced not only by neurons [61] but also different cancer cells, including neuroblastoma cells [62, 63], and may accumulate in the tumour microenvironment. It is noteworthy that in differentiated human neuroblastoma cells VPA and other HDAC inhibitors were recently found to induce the downregulation of TrkB [57], whose expression in neuroblastoma is known to promote aggressiveness, chemotherapy resistance and metastasis [64]. Thus, the ability to induce an imbalance of proapoptotic and prosurvival neurotrophin receptor signalling may constitutes a unique property that can be exploited for the use of VPA as anti-neuroblastoma agent.

As observed in human neuroblastoma cells, in cultured cerebellar granule cells prolonged exposure to VPA upregulated p75NTR and sortilin expression, indicating that these alterations were not restricted to transformed neurons. The VPA induction was mimicked by entinostat, implying a mechanism involving HDAC inhibition. Similarly to the results obtained in neuroblastoma cells, in cerebellar granule cells VPA-induced upregulation of p75NTR and sortilin was accompanied by apoptosis, which was enhanced by the exposure to the p75NTR/sortilin receptor ligand proNGF. HDAC inhibition is considered as one of the major mechanism by which maternal exposure to VPA can cause neurodevelopmental defects and increase the risk of autism in children [18]. VPA administration to neonatal mice has been found to cause autism-like behavioural deficits associated with a marked induction of apoptosis in the external granule cell layer of the cerebellum [65], an area of neural stem cells that express p75NTR in both mice and humans [66, 67]. Interestingly, recent studies have found enhanced levels of p75NTR mRNA and soluble sortilin in the blood of patients with autism [68, 69]. In light of these observations, the present study also suggests the possibility, which remains to be investigated, that overexpression of p75NTR and sortilin may contribute to the neuronal damage occurring in the developing brain following exposure to VPA. In this sense, a main issue to be considered concerns the concentrations reached by VPA in the brain. Previous studies using brain biopsies of patients suffering from a variety of brain tumours reported VPA concentrations, expressed as percent of plasma concentrations, ranging from 6.8 to 27.9% (0.04 to 0.20 mM) [70], which are lower than the VPA concentrations found to affect p75NTR and sortilin levels in neuronal cells. However, with regard to this point one should consider the concentrations that VPA may reach in the embryonic or foetal brain rather than the values detected in postnatal brain specimens. To our knowledge, there is no information on the concentrations reached by VPA in the embryonic brain and associated with teratogenic effects following administration of therapeutic doses to the mother. On the other hand, VPA is known to cross the placenta and its levels in umbilical cord serum may be higher than those in maternal serum [71, 72]. In the neonate, increases in the serum unbound fraction of VPA have been found to be associated with congenital malformations [73]. In vitro studies using blood-brain barrier models have shown an enhanced permeability to VPA in neonatal versus adult rats [74]. An elevated concentration of radiolabeled VPA in embryonic neuroepithelium was observed when the drug was administered to pregnant mice at 8–9 day gestation [75]. Collectively, these studies suggest that under certain pharmacokinetic conditions the immature brain may accumulate VPA at high levels in specific structures, thus leaving open the possibility that sufficient concentrations can be reached to induce changes in neuronal p75NTR and sortilin expression.

## Electronic supplementary material

Below is the link to the electronic supplementary material.

Electronic supplementary material 1 (PDF 9868 kb)

## References

[1] Loscher W (2002). Basic pharmacology of valproate: a review after 35 years of clinical use for the treatment of epilepsy. CNS Drugs.

[2] Henry TR (2003). The history of valproate in clinical neuroscience. Psychopharmacology Bull.

[3] Duenas-Gonzales A, Candelaria M, Perez-Plascencia C, Perez-Cardenas E, de la Cruz-Hernandez E, Herrera LA (2008). Valproic acid as epigenetic cancer drug; preclinical, clinical and transcriptional effects on solid tumors. Cancer Ther Rev.

[4] Chateauvieux S, Morceau F, Dicato M, Diederich M (2010). Molecular and therapeutic potential and toxicity of valproic acid. J Biomed Biotech.

[5] Kwan P, Sills GJ, Brodie MJ (2001). The mechanisms of action of commonly used antiepileptic drugs. Pharmacol Therap.

[6] Machado Ximenes JC, Lima Verde EM, Naffah-Mazzacoratti MG, de Barros Viana GS (2012). Valproic acid, a drug with multiple molecular targets related to its potential neuroprotective action. Neuroscience Medicine.

[7] Phiel CJ, Zhang F, Huang EY, Guenther MG, Lazar MA, Klein PS (2001). Histone deacetylase is a direct target of valproic acid, a potent anticonvulsant, mood stabilizer, and teratogen. J Biol Chem.

[8] Gottlicher M, Minucci S, Zhu P, Kramer OH, Schimpf A, Giavara S, Sleeman JP, Lo Coco F, Nervi C, Pelicci PG, Heinzel T (2001). Valproic acid defines a novel class of HDAC inhibitors inducing differentiation of transformed cells. EMBO J.

[9] Bolden JE, Peart MJ, Johnstone RW (2006). Anticancer activities of histone deacetylase inhibitors. Nat Rev Drug Disc.

[10] Nalivaeva NN, Belyaev ND, Turner AJ (2009). Sodium valproate: an old drug with new roles. Trends Pharmacol Sci.

[11] Chiu C-T, Wang Z, Hunsberger JG, Chuang D-M (2013). Therapeutic potential of mood stabilizers lithium and valproic acid: beyond bipolar disorder. Pharmacol Rev.

[12] Umka J, Mustafa S, Elbeltagy M, Thorpe A, Latif L, Bennett G, Wigmore PM (2010). Valproic acid reduces spatial working memory and cell proliferation in the hippocampus. Neuroscience.

[13] Bollino D, Balan I, Aurelian L (2015). Valproic acid induces neuronal cell death through a novel calpain-dependent necroptosis pathway. J Neurochem.

[14] Jin N, Kovacs AD, Sui Z, Dewhurst S, Maggirwar SB (2005). Opposite effects of lithium and valproic acid on trophic factor deprivation-induced glycogen synthase kinase-3 activation, c-Jun expression and neuronal cell death. Neuropharmacology.

[15] Fujiki R, Sato A, Fujitani M, Yamashita T (2013). A proapoptotic effect of valproic acid on progenitors of embryonic stem cell-derived glutamatergic neurons. Cell Death Dis.

[16] Dragunow M, Greenwood JM, Cameron RE, Narayan PJ, O’Carroll SJ, Pearson AG, Gibbons HM (2008). Valproic acid induces caspase 3-mediated apoptosis in microglial cells. Neuroscience.

[17] Christensen J, Gronborg TK, Sorensen MJ, Schendel D, Parner ET, Pedersen LH, Vestergaard M (2013). Prenatal valproate exposure and risk of autism spectrum disorders and childhood autism. JAMA.

[18] Nicolini C, Fahnestock M (2018). The valproic acid-induced rodent model of autism. Exp Neurol.

[19] Cinatl J, Cinatl J, Driever PH, Kotchetkov R, Pouckova P, Kornhuber B, Schwabe D (1997). Sodium valproate inhibits in vivo growth of human neuroblastoma cells. Anticancer Drugs.

[20] Stockhausen M-Y, Sjolund J, Manetopoulos C, Axelson H (2005). Effects of the histone deacetylase inhibitor valproic acid on Notch signaling in human neuroblastoma cells. Br J Cancer.

[21] Gu S, Tian Y, Chlenski A, Salwen H, Lu Z, Yang Q (2012). Valproic acid shows a potent antitumor effect with alteration of DNA methylation in neuroblastoma. Anticancer Drugs.

[22] Shah RD, Jagtap JC, Mruthyunjaya S, Shelke GV, Pujari R, Das G, Shastry P (2013). Sodium valproate potentiates staurosporine-induced apoptosis in neuroblastoma cells via Akt/surviving independently of HDAC inhibition. J Cell Biochem.

[23] Pan T, Li X, Xie W, Jankovic J, Le W (2005). Valproic acid-mediated Hsp70 induction and anti-apoptotic neuroprotection in SH-SY5Y cells. FEBS Lett.

[24] Aubry J-M, Schwald M, Ballmann E, Karege F (2009). Early effects of mood stabilizers on the Akt/GSK-3β signaling pathway and on cell survival and proliferation. Psychopharmacology.

[25] Schor NF (2005). The p75 neurotrophin receptor in human development and disease. Progr Neurobiol.

[26] Meeker RB, Williams KS (2015). The p75 neurotrophin receptor: at the crossroad of neural repair and death. Neur Regen Res.

[27] Chao MV, Hempstead BL (1995). p75 and Trk: a two-receptor system. Trends Neurosci.

[28] Huang EJ, Reichardt LF (2003). Trk receptors: Roles in neuronal signal transduction. Annu Rev Biochem.

[29] Lee FS, Kim AH, Khursigara G, Chao MV (2001). The uniqueness of being a neurotrophin receptor. Curr Opin Neurobiol.

[30] Nykjaer A, Willnow TE, Petersen CM (2005). p75^NTR^- live or let die. Curr Opin Neurobiol.

[31] Petersen CM, Nielsen MS, Nykjaer A, Jacobsen L, Tommerup N, Rasmussen HH, Roigaard H, Gliemann J, Madsen P, Moestrup SK (1997). Molecular identification of a novel candidate sorting receptor purified from human brain by receptor-associated protein affinity chromatography. J Biol Chem.

[32] Nykjaer A, Lee R, Teng KK, Jansen P, Madsen P, Nielsen MS, Jacobsen C, Kllemannel M, Schwarz E, Willnow TE, Hempstead BL, Petersen CM (2004). Sortilin is essential for proNGF-induced neuronal cell death. Nature.

[33] Nykjaer A, Willnow TE (2012). Sortilin: a receptor to regulate neuronal viability and function. Trends Neurosci.

[34] Olianas MC, Dedoni S, Boi M, Onali P (2008). Activation of nociceptin/orphanin FQ-NOP receptor system inhibits tyrosine hydroxylase phosphorylation, dopamine synthesis, and dopamine D1 receptor signaling in rat nucleus accumbens and dorsal striatum. J Neurochem.

[35] Dedoni S, Olianas MC, Ingianni A, Onali P (2012). Type I interferons impair BDNF-induced cell signaling and neurotrophiic activity in differentiated human SH-SY5Y neuroblastoma cells and mouse primary cortical neurons. J Neurochem.

[36] Grob P, Ross AH, Koprowski H, Bothwell M (1985). Characterization of the human melanoma nerve growth factor receptor. J Biol Chem.

[37] Mai A, Massa S, Pezzi R, Simeoni S, Rotili D, Nebbioso A, Scognamiglio A, Altucci L, Loidl P, Brosch G (2005). Class II (IIa)-selective histone deacetylase inhibitors. 1. Synthesis and biological evaluation of novel (aryloxopropenyl)pyrrolyl hydroxamides. J Med Chem.

[38] Haggarty SJ, Koeller KM, Wong JC, Grozinger CM, Schreiber SL (2003). Domain-selective small-molecule inhibitor of histone deacetylase 6 (HDAC6)-mediated tubulin deacetylation. Proc Natl Acad Sci USA.

[39] Balasubramanian S, Ramos J, Luo W, Sirisawad M, Verner E, Buggy JJ (2008). A novel histone deacetylase 8 (HDAC8)-specific inhibitor PCI-34051 induces apoptosis in T-cell lymphomas. Leukemia.

[40] Wang C, Liu Z, Woo C-W, Li Z, Wang L, Wei JS, Marquez VE, Bates SE, Jin Q, Khan J, Ge K, Thiele CJ (2012). EZH2 mediates epigenetic silencing of neuroblastoma suppressor genes CASZ1, CLU, RUNX3, and NGFR. Cancer Res.

[41] Liu Z, Yang X, Li Z, McMahon C, Sizer C, Barenboim-Stapleton L, Bliskovsky V, Mock B, Ried T, London WB, Maris J, Khan J, Thiele CJ (2011). CASZ1, a candidate tumor-suppressor gene, suppresses neuroblastoma tumor growth through reprogramming gene expression. Cell Death Differ.

[42] Tan J, Yang X, Zhuang L, Jiang X, Chen W, Lee PL, Karuturi RKM, Tan PBO, Liu ET, Yu Q (2007). Pharmacologic disruption of polycomb-repressive complex 2-mediated gene repression selectively induces apoptosis in cancer cells. Genes Dev.

[43] Knutson SK, Warholic NM, Wigle TJ, Klaus CR, Allain CJ, Raimondi A, Porter Scott M, Chesworth R, Moyer MP, Copeland RA, Richon VM, Pollock RM, Kuntz KW, Keilhack H (2013). Durable tumor regression in genetically altered malignant rhabdoid tumors by inhibition of methyltransferase EZH2. Proc Natl Acad Sci USA.

[44] Feng D, Kim T, Ozkan M, Torkin R, Teng KK, Hempstead BL, Garcia KC (2010). Molecular and structural insight into proNGF engagement of p75NTR and sortilin. J Mol Biol.

[45] Salehi AH, Xanthoudakis S, Berker PA (2002). NRAGE, a p75 neurotrophin receptor-interacting protein, induces caspase activation and cell death through a JNK-dependent mitochondrial pathway. J Biol Chem.

[46] Davis RJ (2000). Signal transduction by the JNK group of MAP kinases. Cell.

[47] Dechant G, Barde Y-A (2002). The neurotrophin receptor p75^NTR^: novel functions and implications for diseases of the nervous system. Nat Neurosci.

[48] Hempstead BL (2009). Commentary: regulating proNGF action: multiple targets for therapeutic intervention. Neurotox Res.

[49] Urdiales JL, Becker E, Andrieu M, Thomas A, Jullien J, van Grunsven LA, Menut S, Evan GI, Martin-Zanca D, Rudkin BB (1998). Cell cycle phase-specific surface expression of nerve growth factors receptors TrkA and p75^NTR^. J Neurosci.

[50] Zhang C, Helmsing S, Zagrebelsky M, Schirrmann T, Marschall ALJ, Schungel M, Korte M, Hust M, Dubel S (2012) Suppression of p75 neurotrophin receptor surface expression with intrabodies influences Bcl-xL mRNA expression and neurite outgrowth in PC12 cells. PLoS ONE 7:e30684 10.1371/journal.pone.0030684 PMC3265506 22292018

[51] Sarret P, Krzywkowski P, Segal L, Nielsen MS, Petersen CM, Mazella J, Stroh T, Beaudet A (2003). Distribution of NTS3 receptor/sortilin mRNA and protein in the rat central nervous system. J Comp Neurol.

[52] Nakamura K, Namekata K, Harada C, Harada T (2007). Intracellular sortilin expression pattern regulates proNGF-induced naturally occurring cell death during development. Cell Death Differ.

[53] Kim T, Hempstead BL (2009). NRH2 is a trafficking switch to regulate sortilin localization and permit proneurotrophin-induced cell death. EMBO J.

[54] Suzuki K, Hiramoto A, Okumura T (2015). A case report on reversible Pelger-Huet anomaly depending on serum free fraction of valproic acid. Brain Dev.

[55] Munster P, Marchion D, Bicaku E, Schmitt M, Lee H, DeConti R, Simon G, Fishman M, Minton S, Garrett C, Chiappori A, Lush R, Sullivan D, Daud A (2007). Phase I trial of histone deacetylase inhibition by valproic acid followed by the topoisomerase II inhibitor epirubicin in advanced solid tumors: a clinical and translational study. J Clin Oncol.

[56] Munster P, Marchion D, Bicaku E, Lacevic M, Kim J, Centeno B, Daud A, Neuger A, Minton S, Sullivan D (2009). Clinical and biological effects of valproic acid as a histone deacetylase inhibitor on tumor and surrogate tissues: phase I/II trial of valproic acid and epirubicin/FEC. Clin Cancer Res.

[57] Dedoni S, Marras L, Olianas MC, Ingianni A, Onali P (2019). Downregulation of TrkB expression and signaling by valproic acid and other histone deacetylase inhibitors. J Pharmacol Exp Ther.

[58] Fiskus W, Pranpat M, Balasis M, Herger B, Rao R, Chinnaiyan A, Atadja P, Bhalla K (2006). Histone deacetylase inhibitors deplete enhancer of zeste 2 and associated polycomb repressive complex 2 proteins in human acute leukemia cells. Mol Cancer Ther.

[59] Bunone G, Mariotti A, Compagni A, Morandi E, Della Valle G (1997). Induction of apoptosis by p75 neurotrophin receptor in human neuroblastoma cells. Oncogene.

[60] Schulte JH, Pentek F, Hartmann W, Schramm A, Friedrichs N, Ora I, Koster J, Versteeg R, Kirfel J, Buettner R, Eggert A (2009). The low-affinity neurotrophin receptor, p75, is upregulated in ganglioneuroblastoma/ganglioneuroma and reduces tumorigenity of neuroblastoma cells *in vivo*. Int J Cancer.

[61] Hasan W, Pedchenko T, Krizsan-Agbas D, Baum L, Smith PG (2003). Sympathetic neurons synthesize and secrete pro-nerve growth factor protein. J Neurobiol.

[62] Bradshaw RA, Pundavela J, Biarc J, Chalkley RJ, Burlingame AL, Hondermarck H (2015). NGF and proNGF: regulation of neuronal and neoplastic responses through receptor signaling. Adv Biol Regul.

[63] Battaglia-Hsu S-f, Akchiche N, Noel N, Alberto J-M, Jeannesson E, Orozco-Barrios CE, Martinez-Fong D, Daval J-L, Gueant J-L (2009). Vitamin B12 deficiency reduces proliferation and promotes differentiation of neuroblastoma cells and up-regulates PP2A, proNGF, and TACE. Proc Natl Acad Sci USA.

[64] Thiele CJ, Li Z, McKee AE (2009). On Trk - The TrkB signal transduction pathway is an increasingly important target in cancer biology. Clin Cancer Res.

[65] Yochum CL, Dowling P, Reuhl KR, Wagner GC, Ming X (2008). VPA-induced apoptosis and behavioural deficits in neonatal mice. Brain Res.

[66] Barnes M, Eberhart CG, Collins R, Tihan T (2009). Expression of p75NTR in fetal brain and medulloblastomas: evidence of a precursor cell marker and its persistence in neoplasia. J Neurooncol.

[67] Kuchler J, Hartmann W, Waha A, Koch A, Endl E, Wurst P, Kindler D, Mikeska T, Waha A, Goodyer CG, Buttner R, Schilling K, Pietsch T (2011). p75NTR induces apoptosis in medulloblastoma cells. Int J Cancer.

[68] Segura M, Pedreno C, Obiols J, Taurines R, Pamias M, Grunblatt E, Gella A (2015). Neurotrophin blood-based gene expression and social cognition analysis in patients with autism spectrum disorder. Neurogenetics.

[69] Patel AB, Tsilioni I, Leeman SE, Theoharides TC (2016). Neurotensin stimulates sortilin and mTOR in human microglia inhibitable by methoxyluteolin, a potential therapeutic target for autism. Proc Natl Acad Sci USA.

[70] Vajda FJE, Donnan GA, Phillips J, Blandin PF (1981). Human brain, plasma, and cerebrospinal fluid concentration of sodium valproate after 72 hours of therapy. Neurology (Ny).

[71] Nau H, Rating D, Koch S, Hauser I, Helge H (1981). Valproic acid and its metabolites: placental transfer, neonatal pharmacokinetics, transfer via mother’s milk and clinical status in neonates of epileptic mothers. J Pharmacol Exp Ther.

[72] Froescher W, Gugler R, Niesen M, Hoffmann F (1984). Protein binding of valproic acid in maternal and umbilical cord serum. Epilepsia.

[73] Sparla S, Hogeman P, van Gemert M, Swart E, Maligre M (2017). Valproic acid during pregnancy: case report of a child with congenital malformations due to fetal valproate syndrome, and a high unbound serum level of valproic acid at birth. Int J Epilepsy.

[74] Takata F, Dohgu S, Yamauchi A, Matsumoto J, Machida T, Fujishita K, Shibata K, Shinozaki Y, Sato K, Kataoka Y, Koizumi S (2013). *In vitro* blood-brain barrier models using brain capillary endothelial cells isolated from neonatal and adult rats retain age-related barrier properties. PlosOne.

[75] Dencker L, Nau H, D’Argy R (1990). Marked accumulation of valproic acid in embryonic neuroepithelium of the mouse during early organogenesis. Teratology.

