# Supplementary material for: Valproic acid upregulates the expression of the p75NTR/sortilin receptor complex to induce neuronal apoptosis
Source: Apoptosis. 2020 Jul 25;25(9):697–714. doi: 10.1007/s10495-020-01626-0 (PMC7527367; doi:10.1007/s10495-020-01626-0)

Manuscript # APPT-D-20-0074

Western blots used to generate the data reported in scatter plots

Fig. 1A

p75NTR / actin

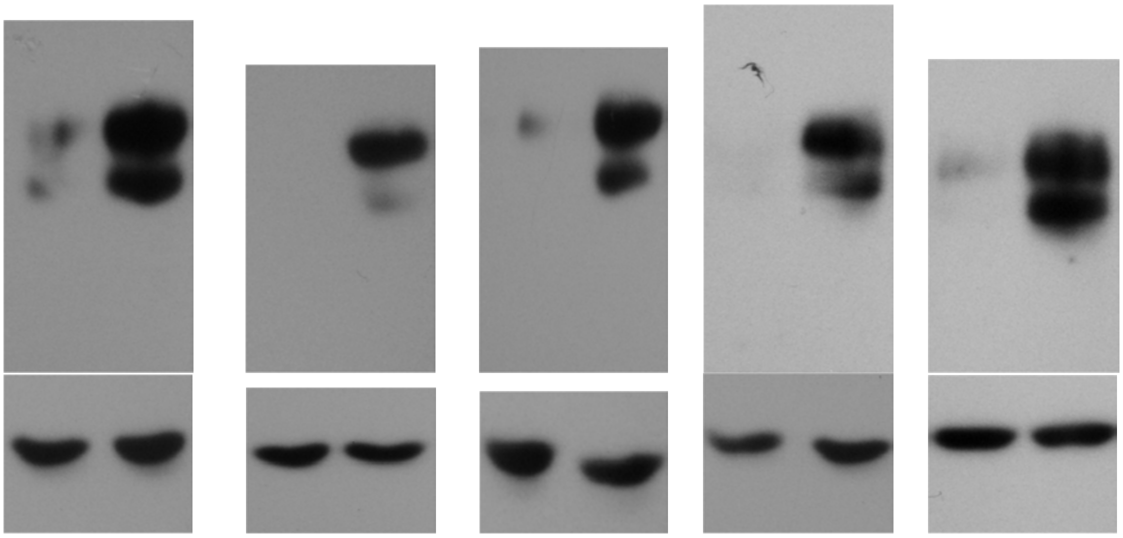

Fig. 1B

sortilin / actin

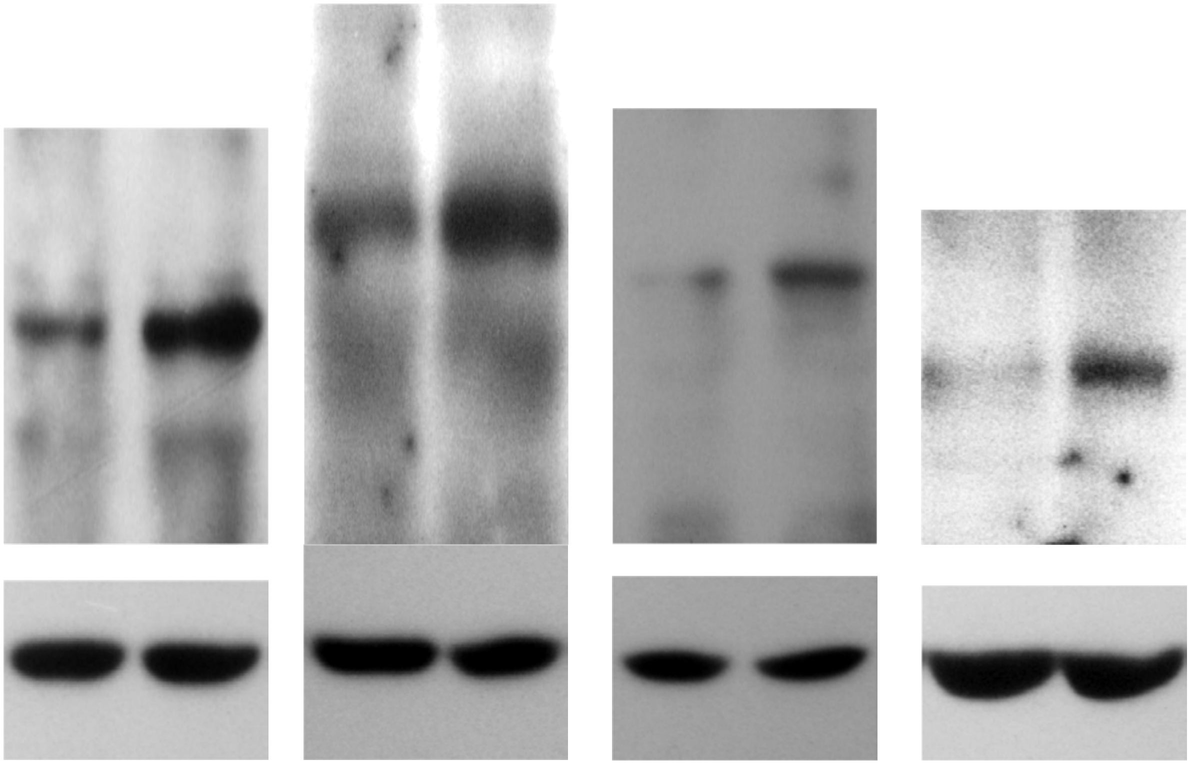

Fig. 1D

LAN-1

p75NTR / actin

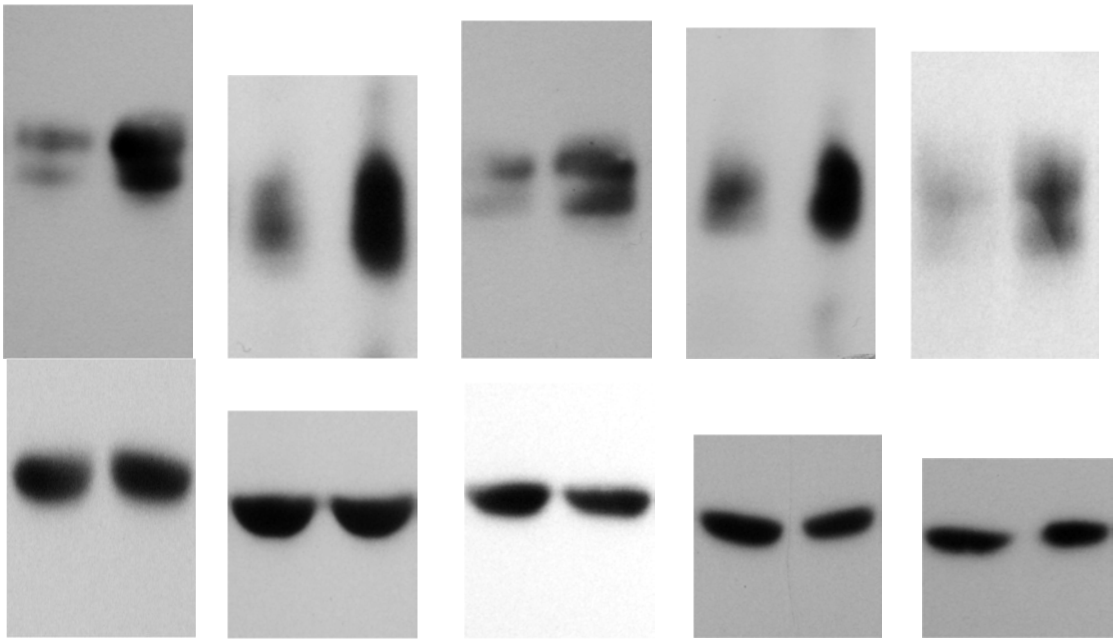

Fig 1E

sortilin / actin

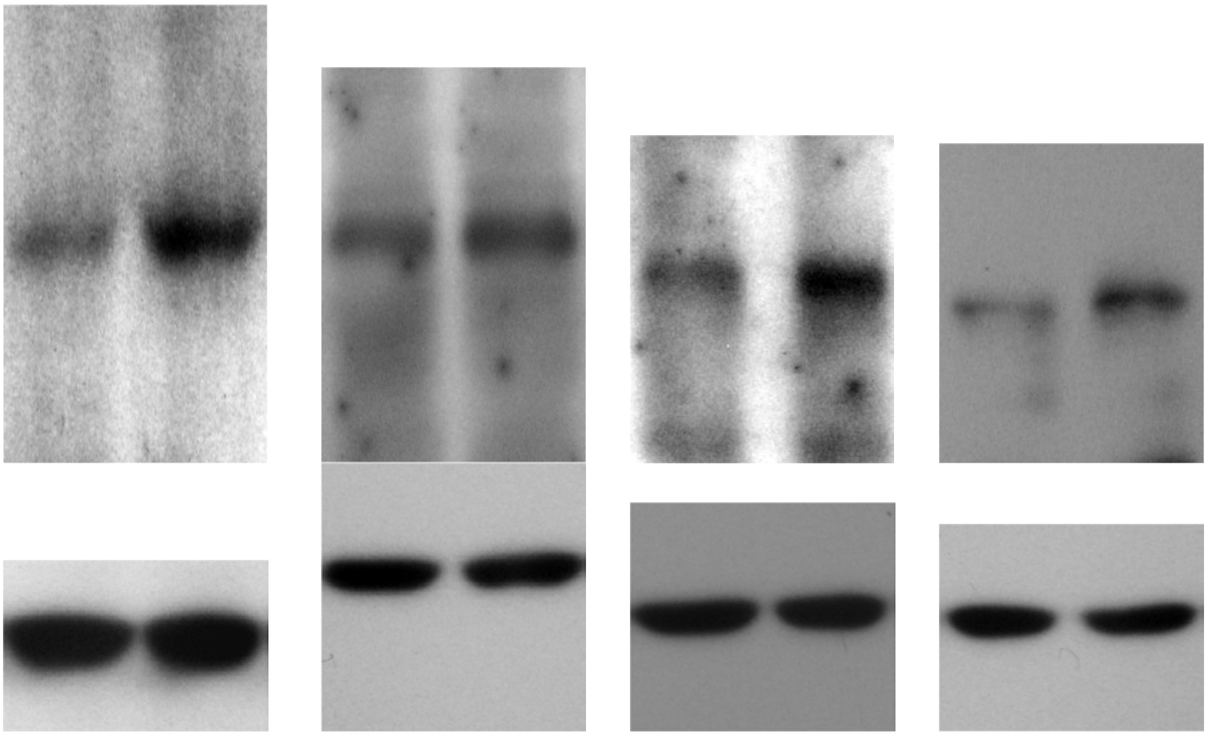

Fig. 2A

SH-SY5Y

p75NTR / pan-cadherin

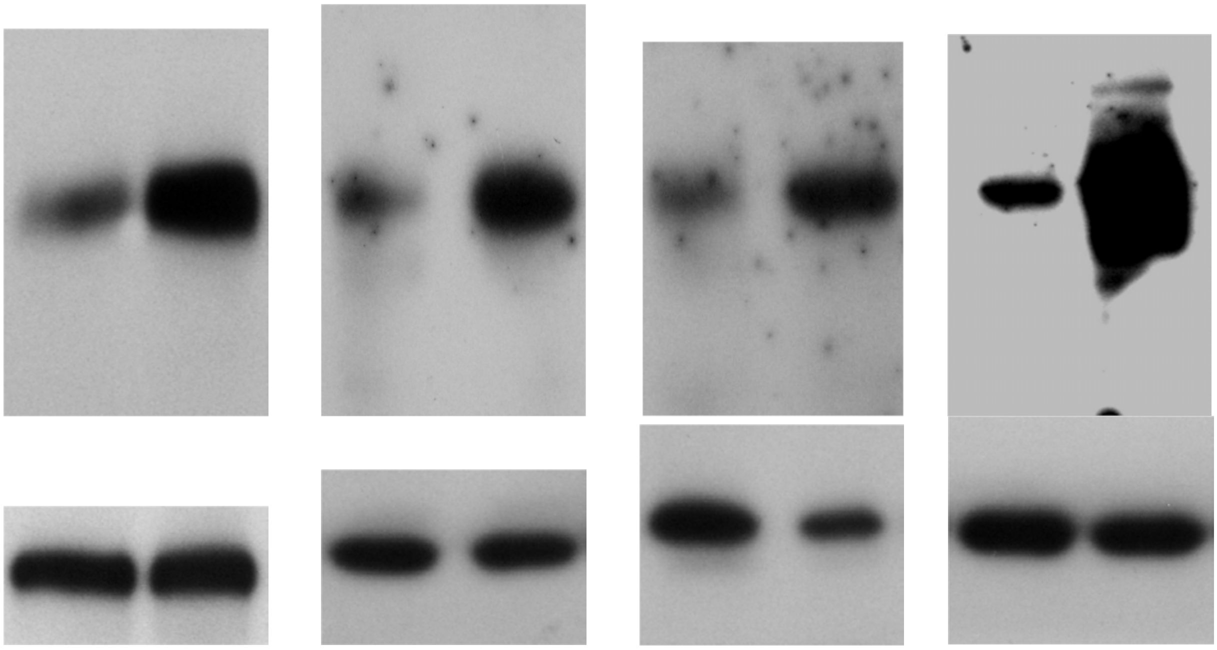

Fig. 2A

sortilin / pan-cadherin

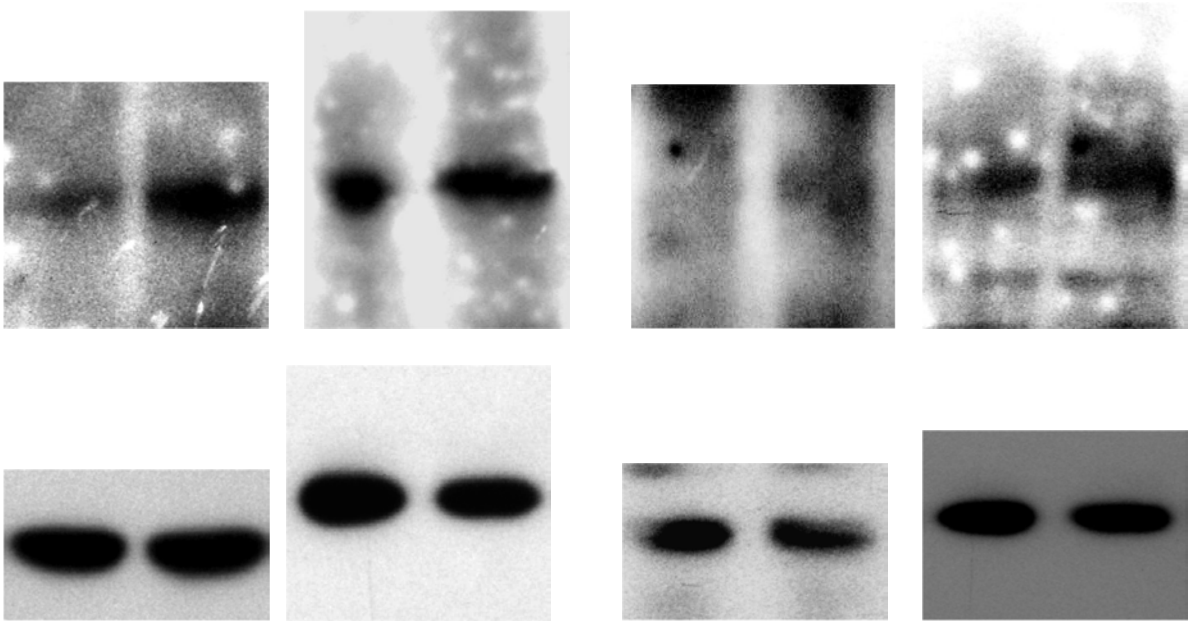

Fig. 2A

LAN-1

p75NTR / pan-cadherin

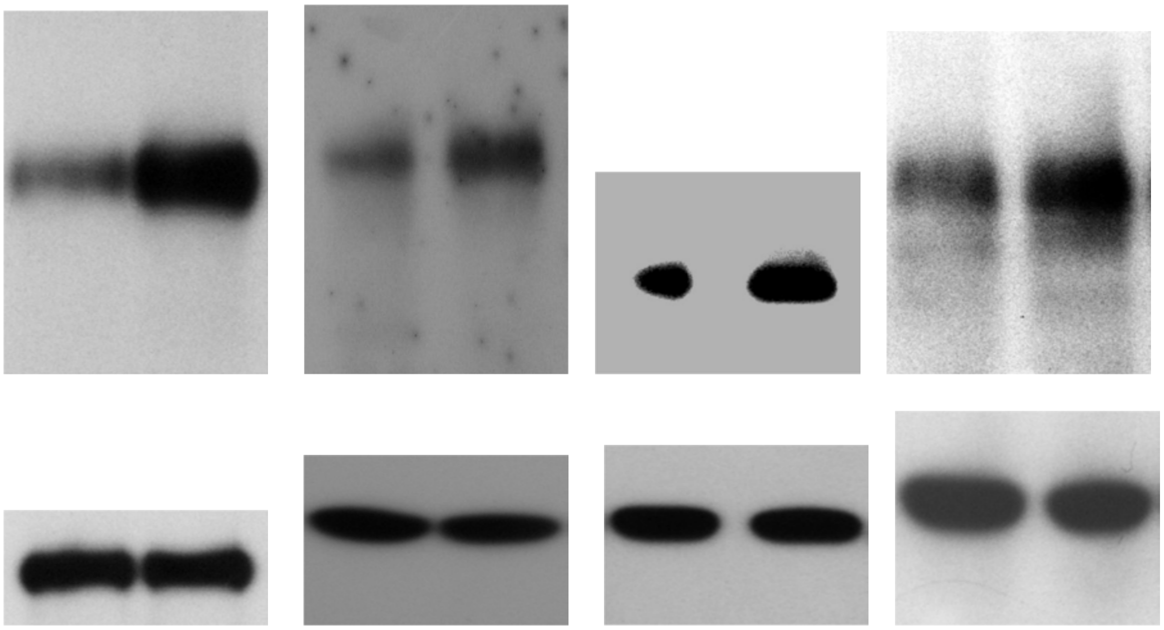

Fig.2A
sortilin / pan-cadherin

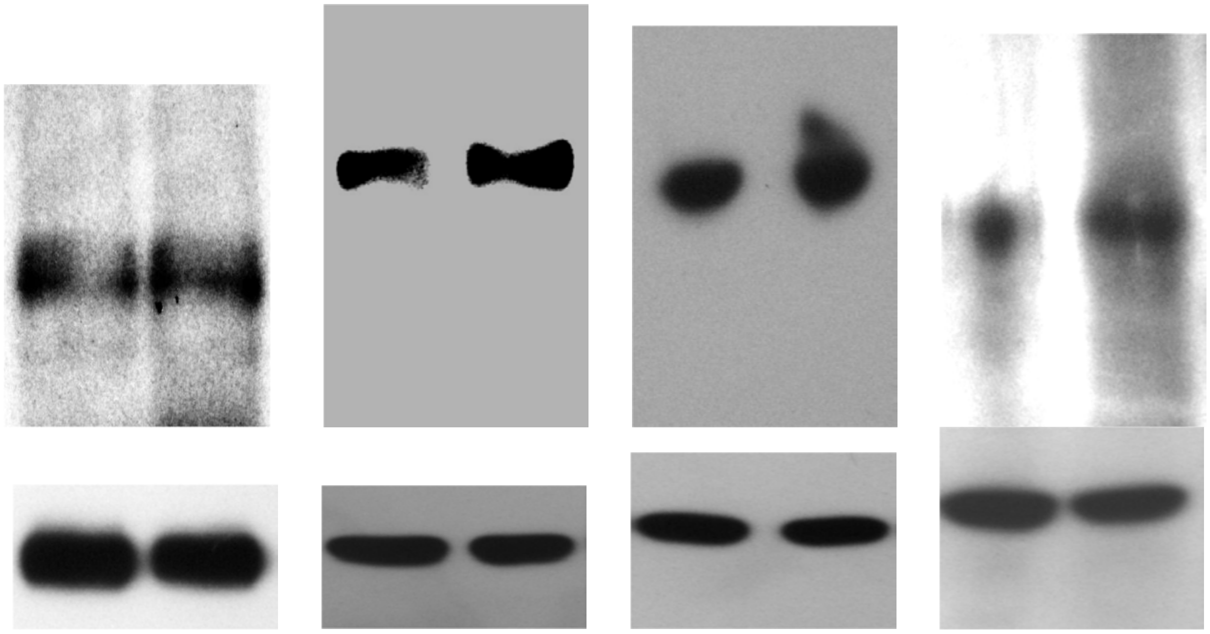

Fig, 3A

p75NTR / actin

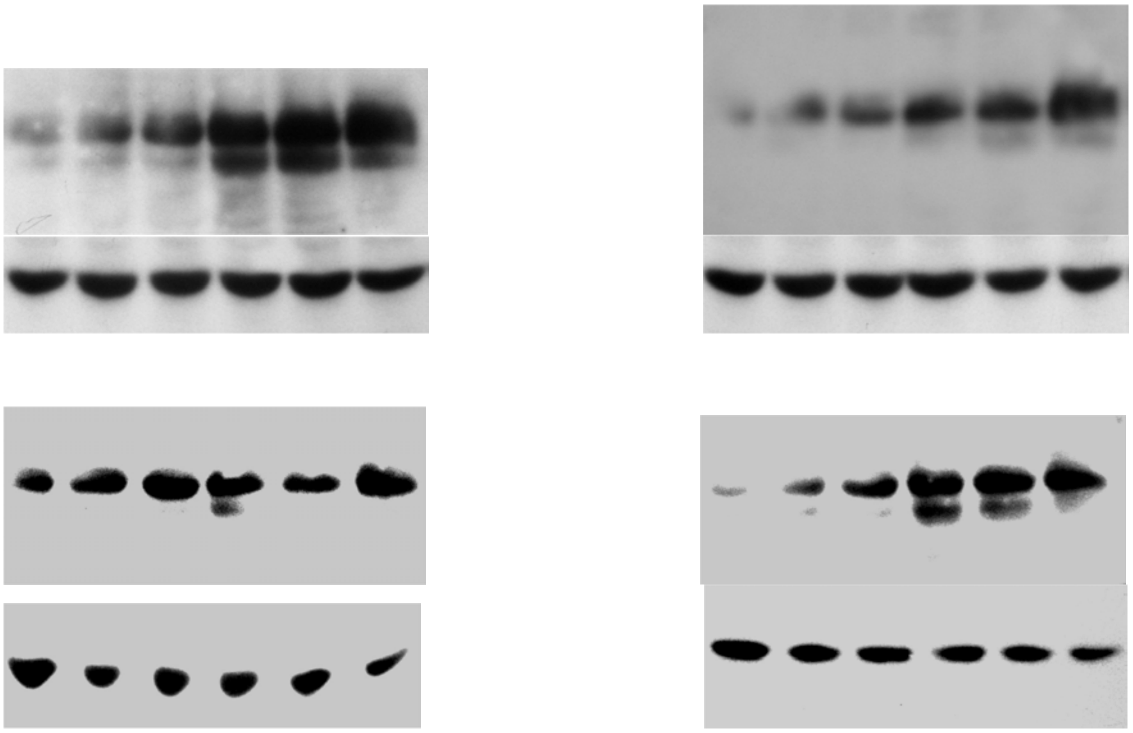

Fig. 3B

p75NTR / actin

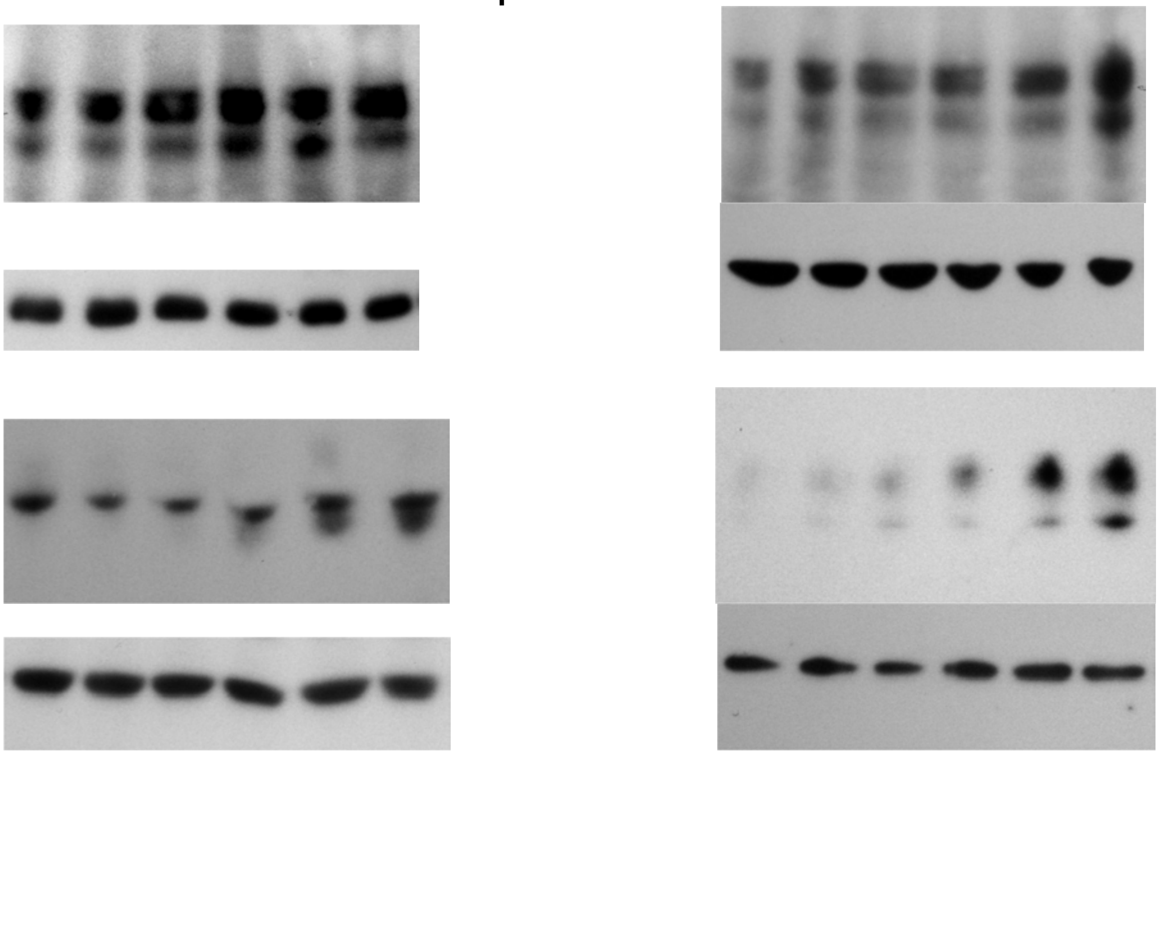

Fig. 3C

p75NTR / actin

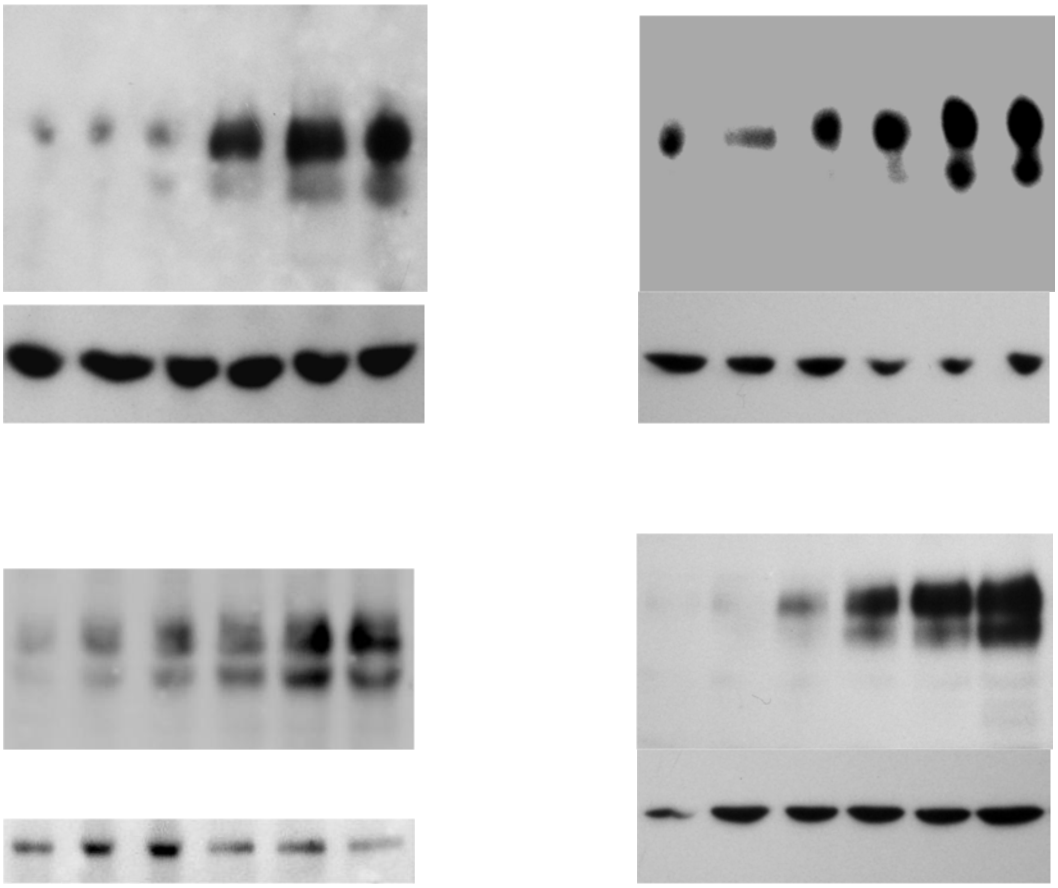

Fig. 3D

p75NTR / actin

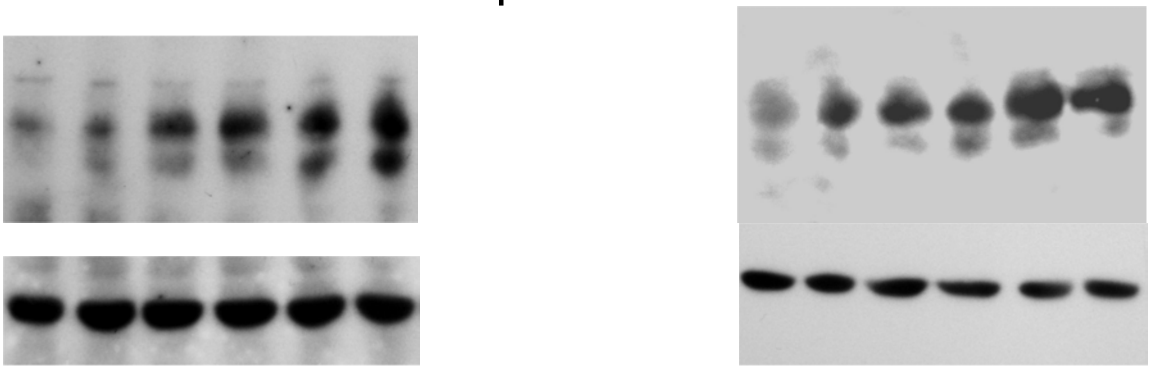

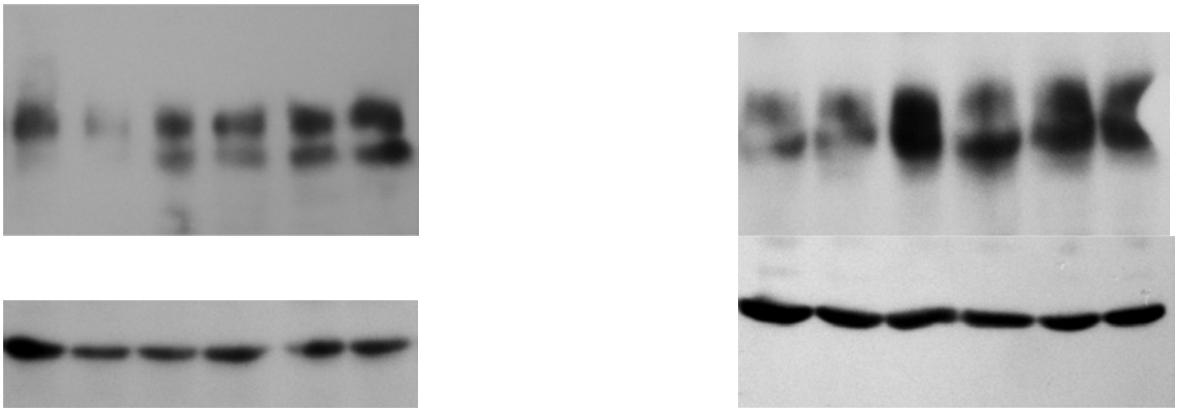

Fig.3E

sortilin / actin

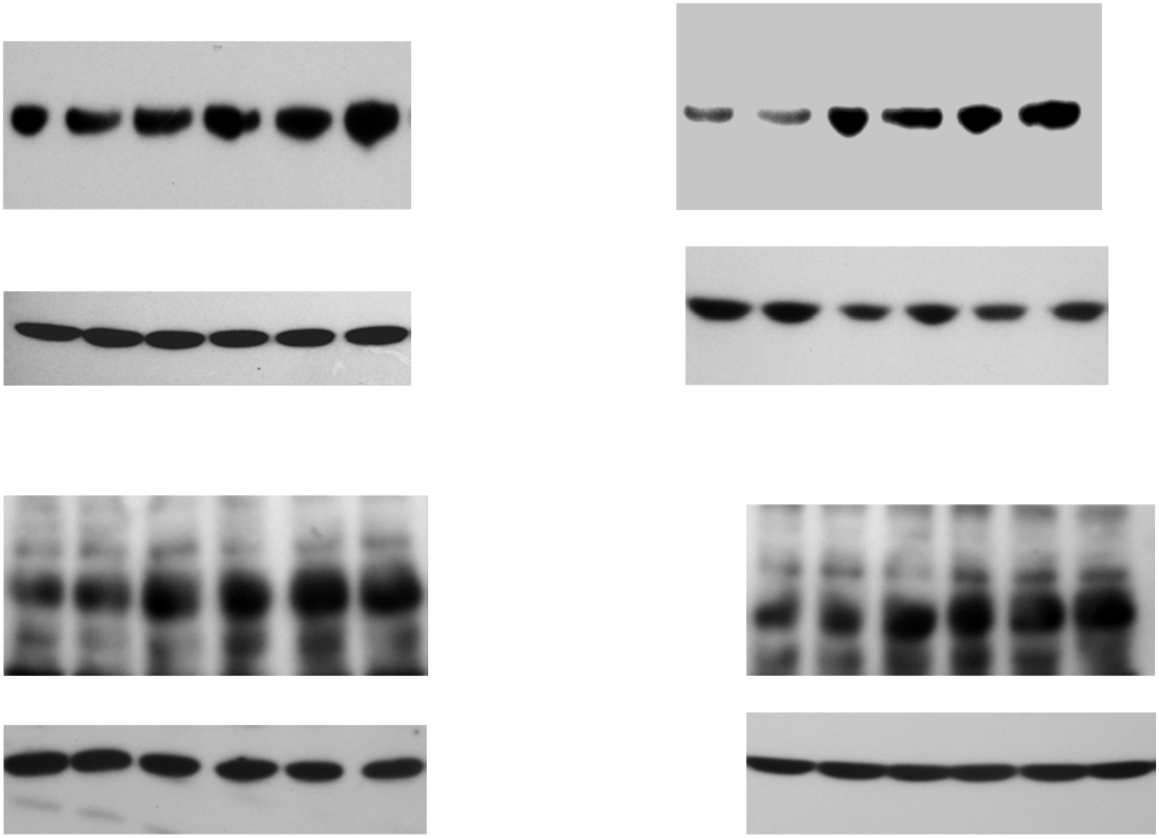

Fig. 3F

sortilin / actin

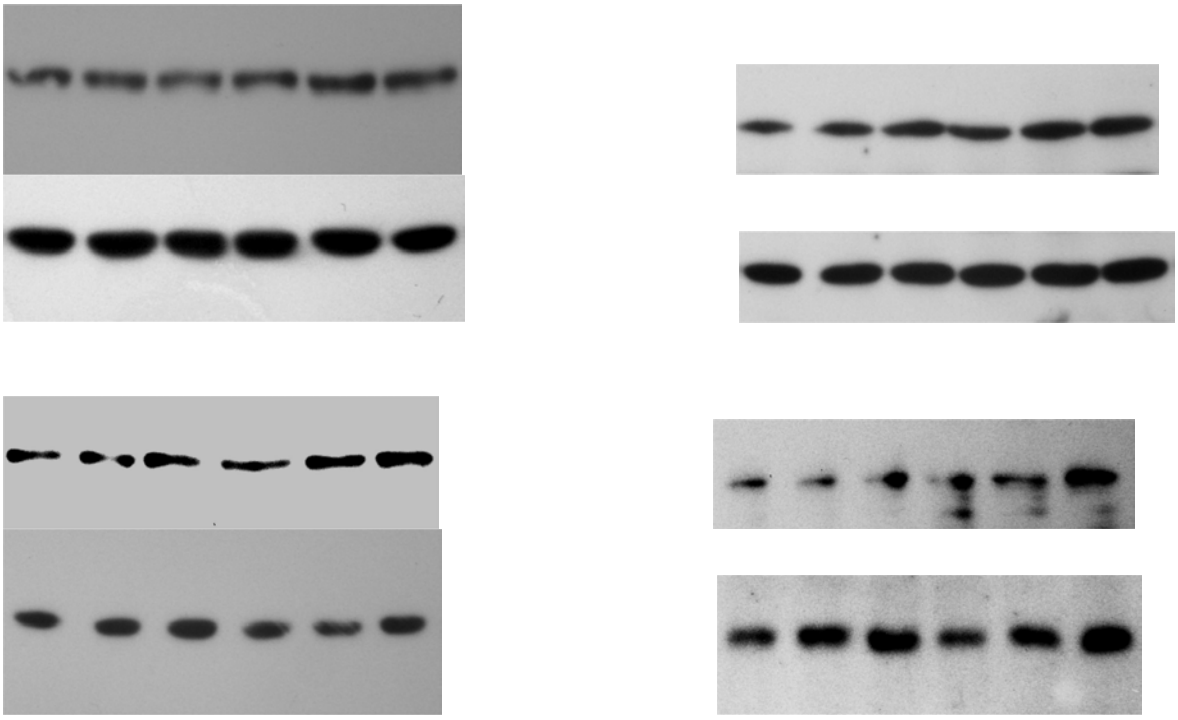

Fig. 4A

p75NTR / actin

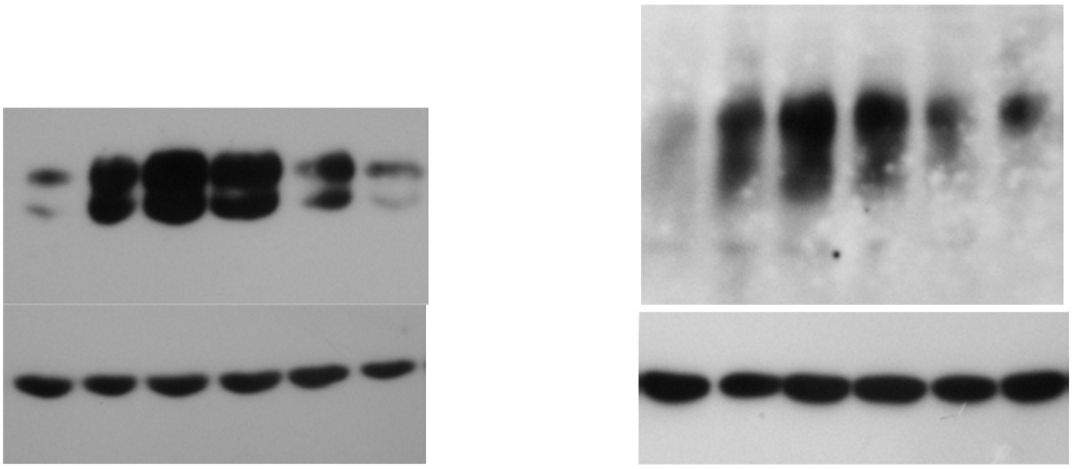

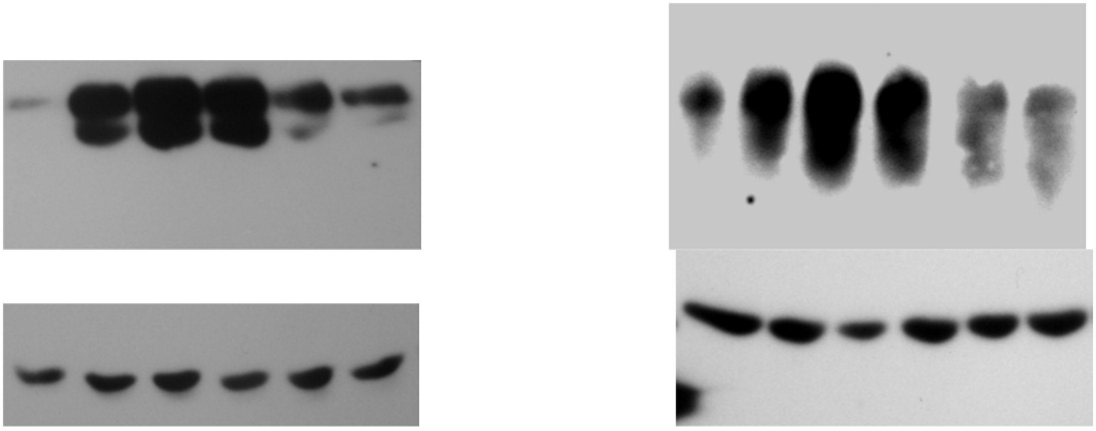

Fig. 4B

p75NTR / actin

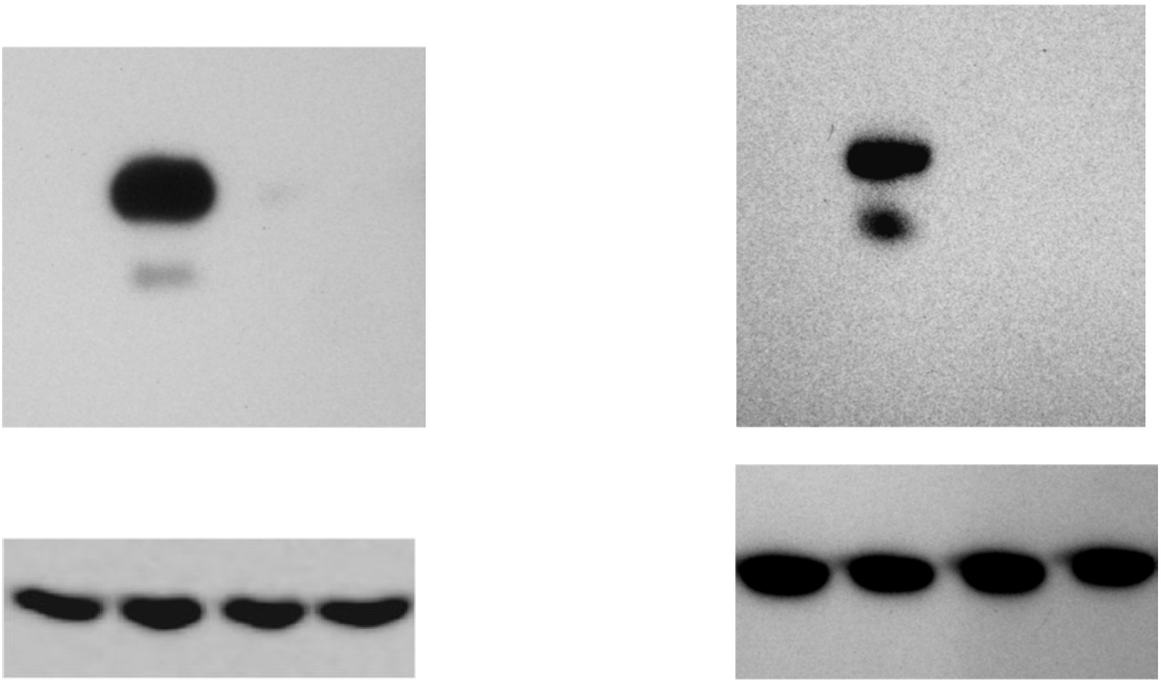

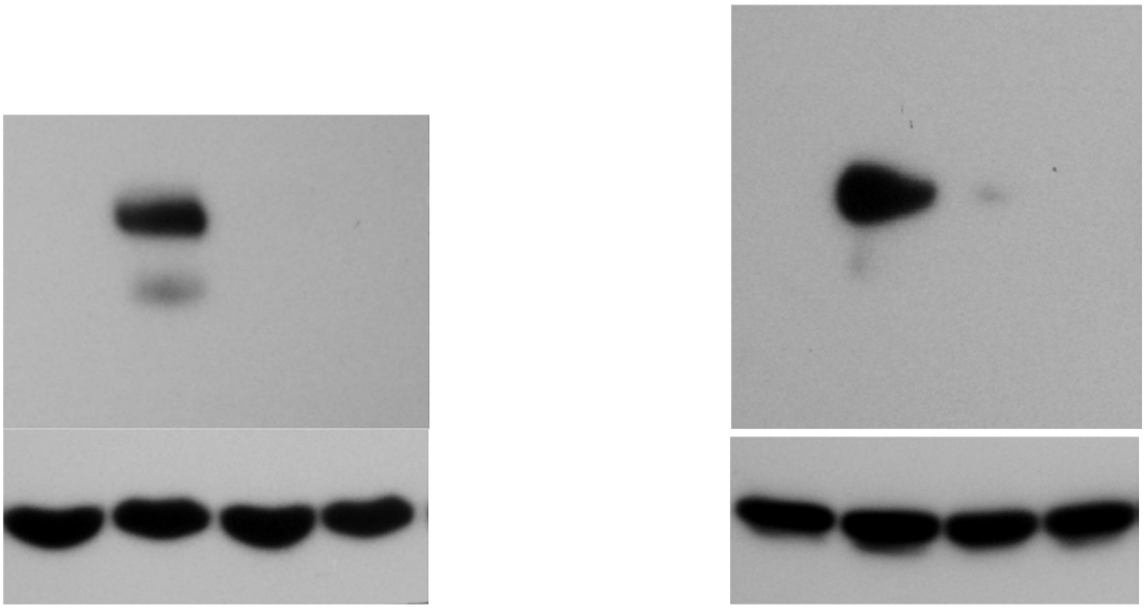

Fig. 4C

pP75NTR / actin

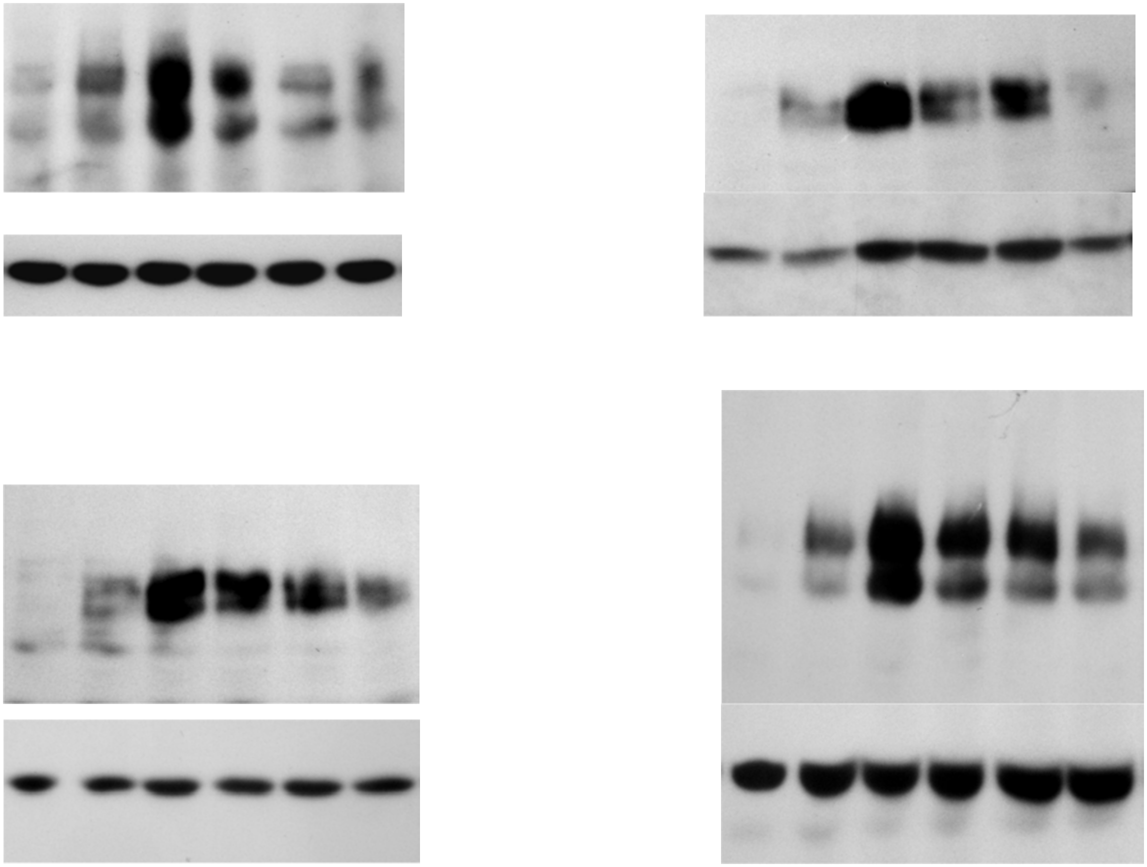

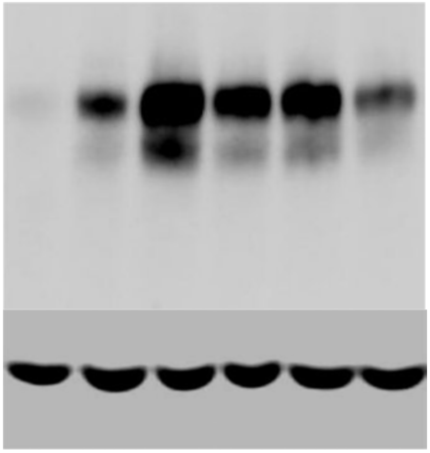

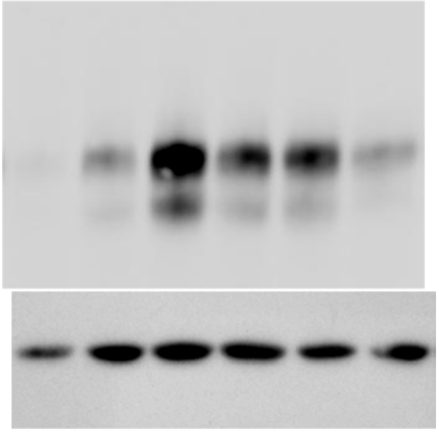

Fig. 4D

p75NTR / actin

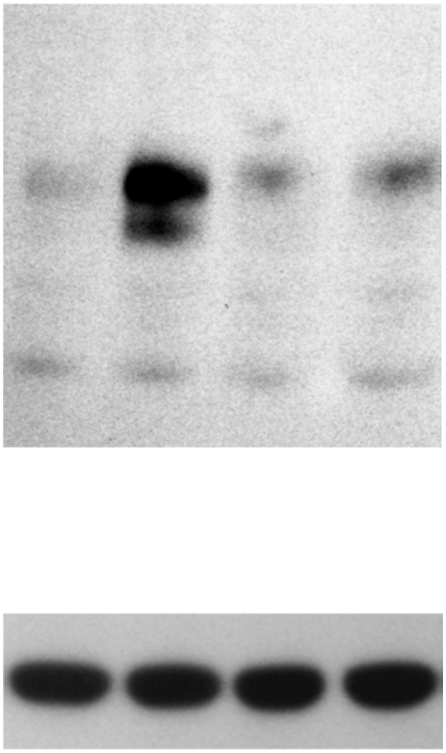

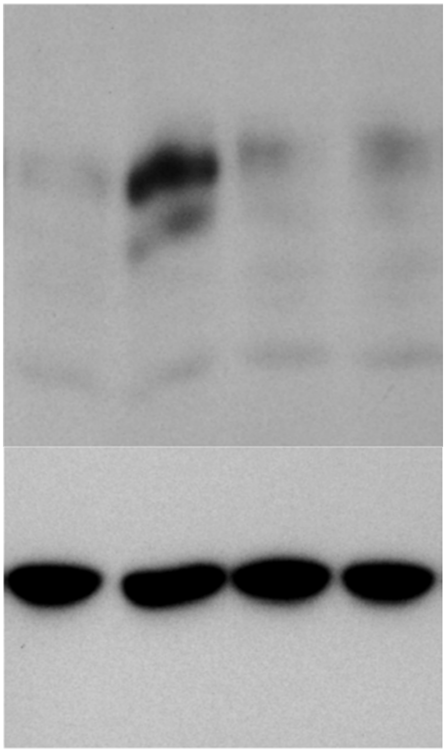

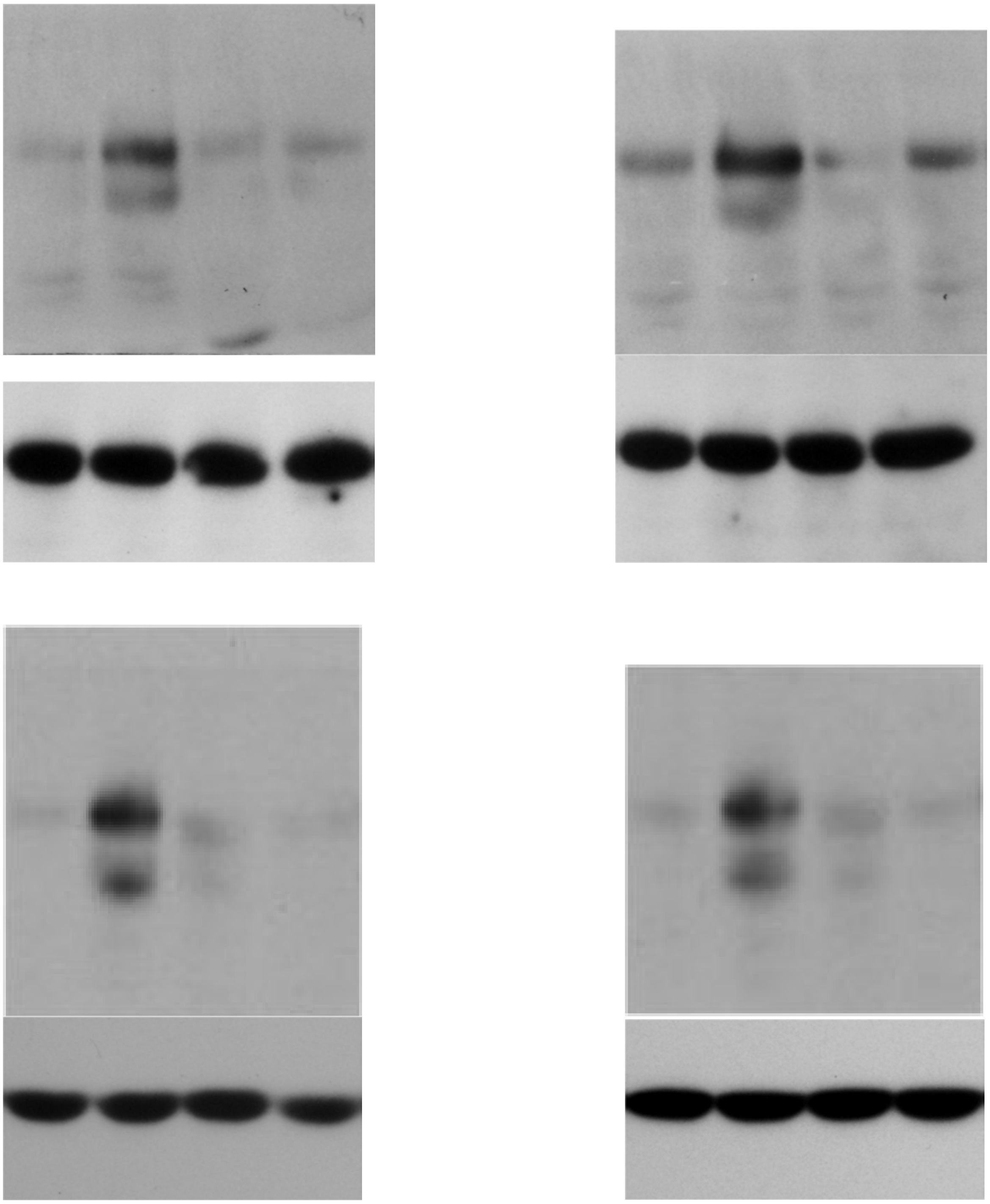

Fig. 4E

sortilin / actin

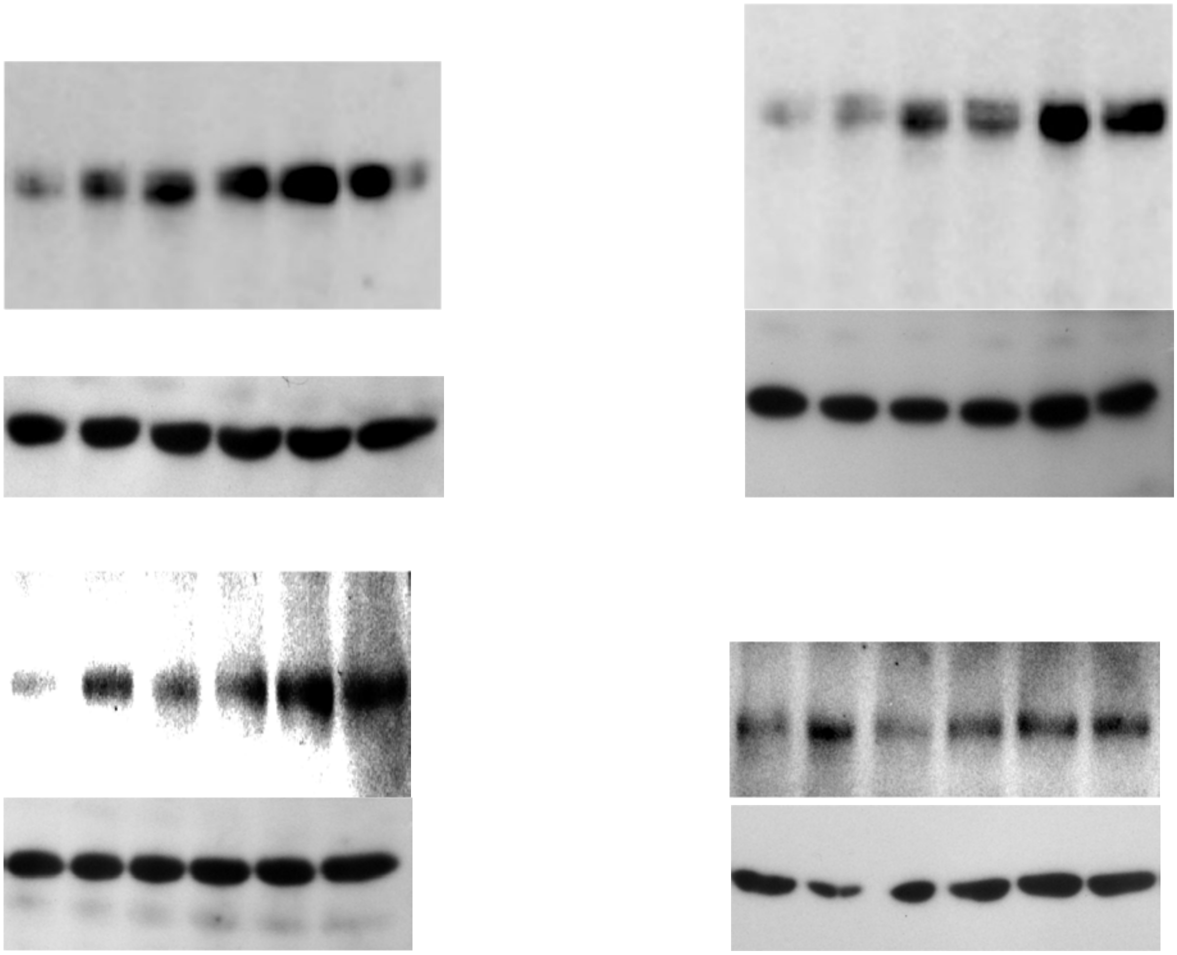

Fig. 4F

sortilin / actin

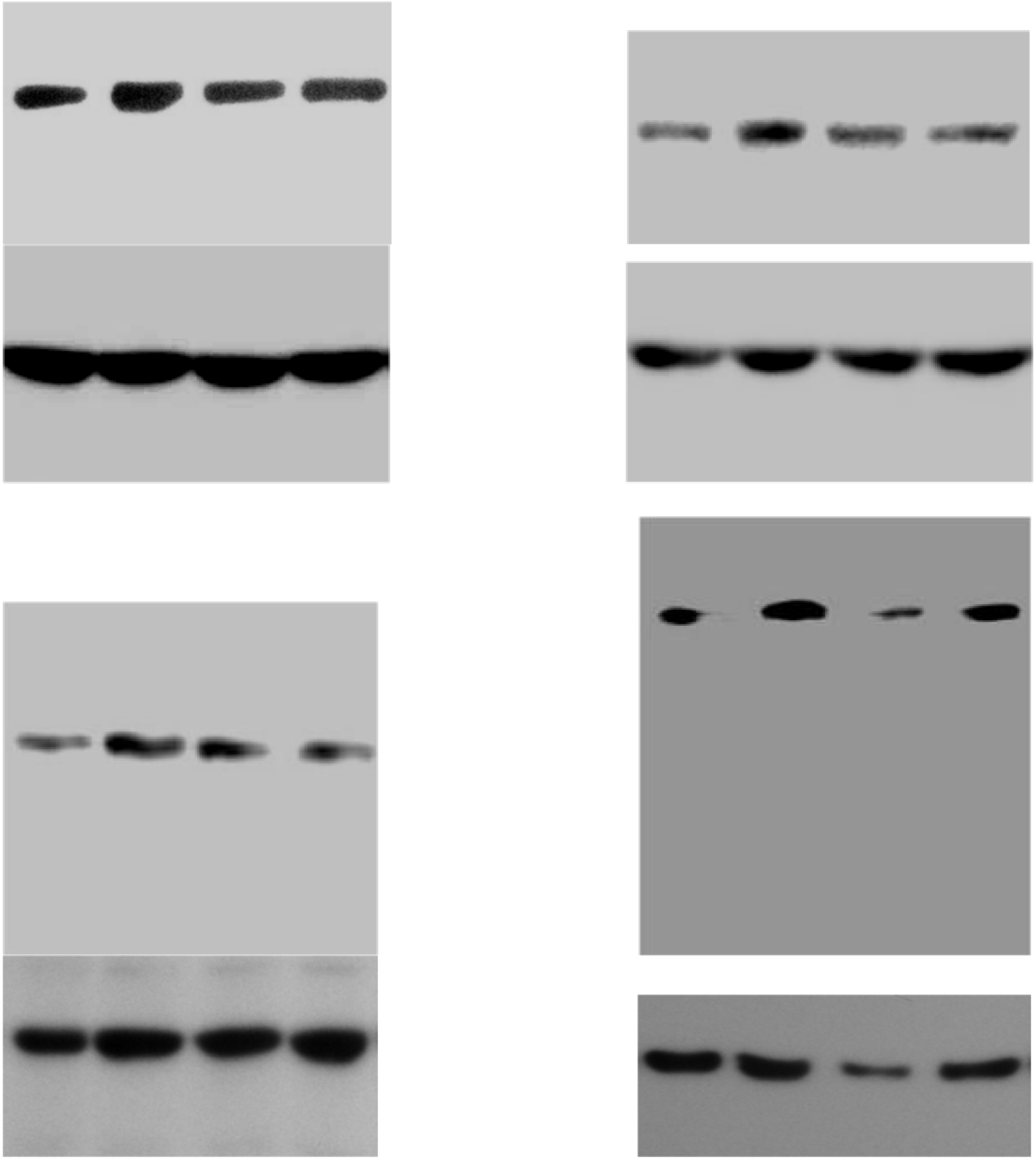

Fig. 4G

sortilin / actin

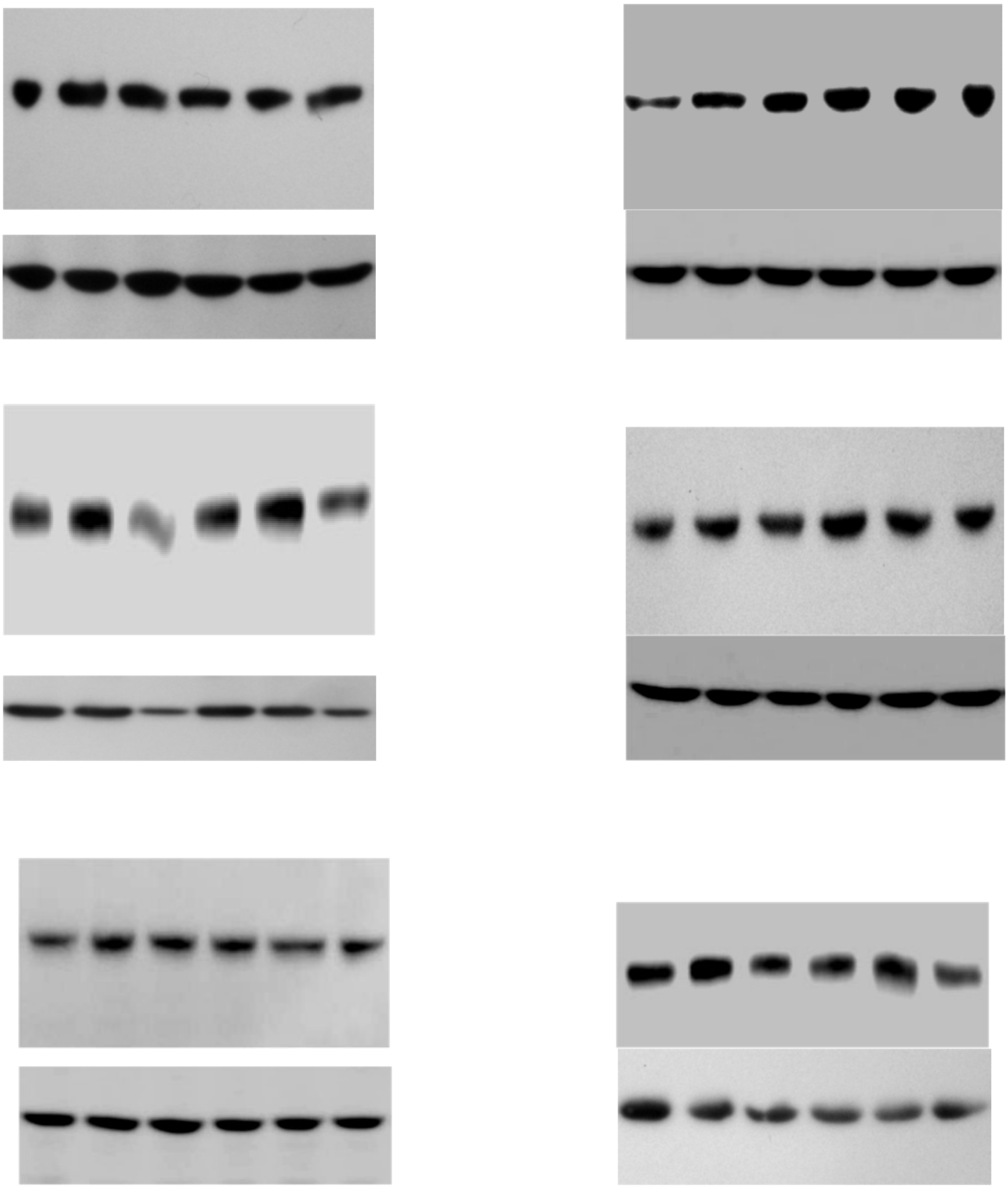

Fig. 4H

sortilin / actin

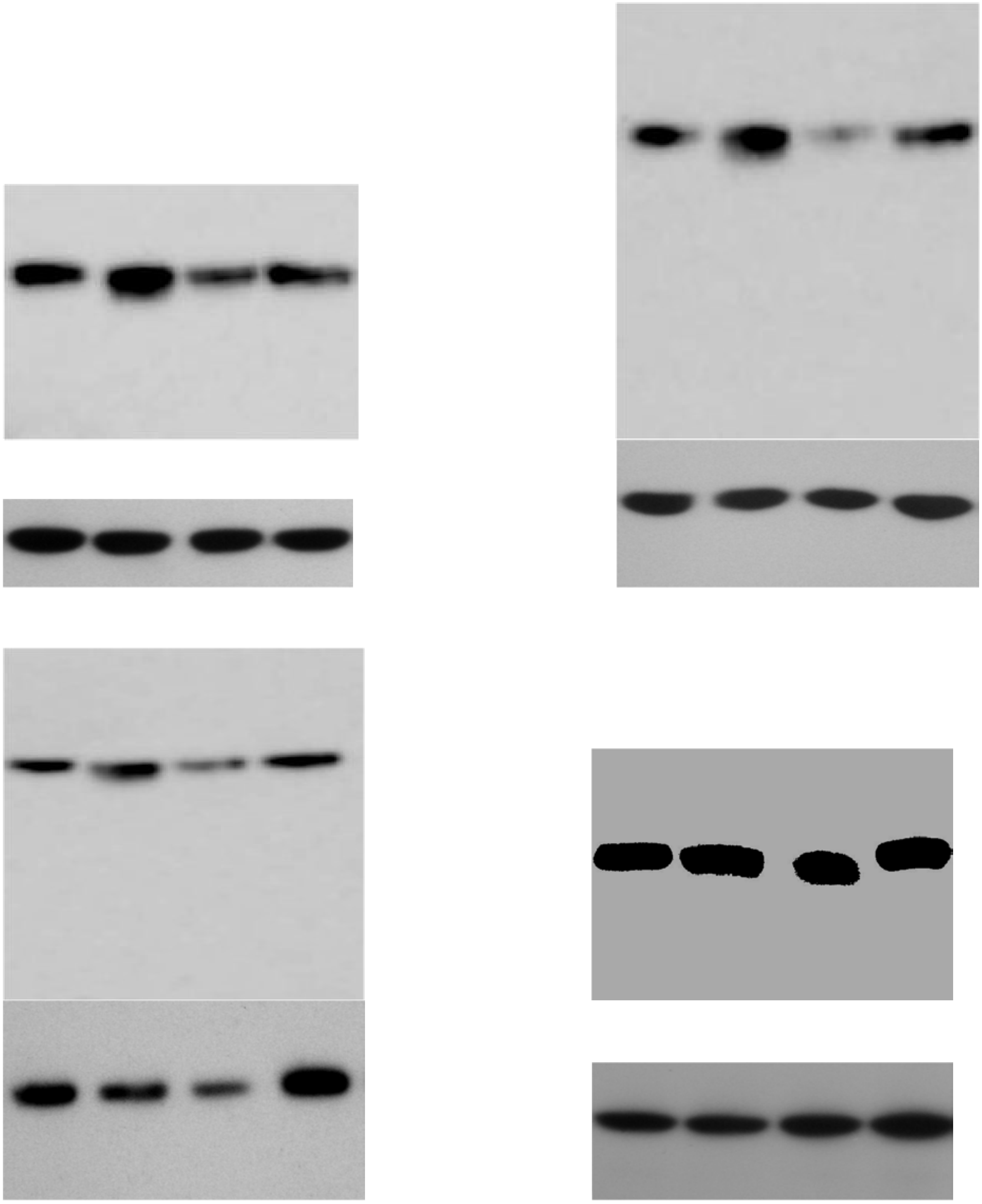

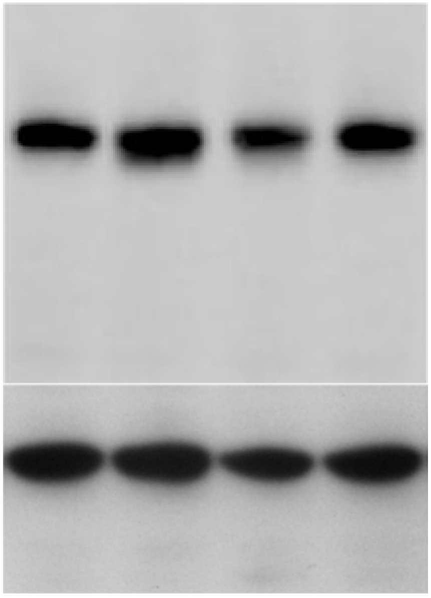

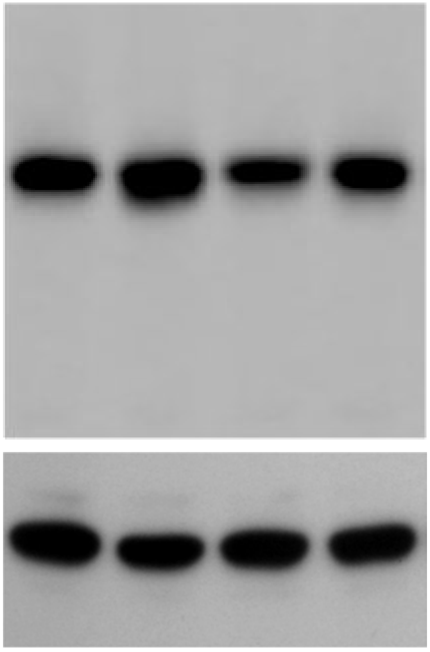

Fig. 5A

HDAC1-p75NTR / actin

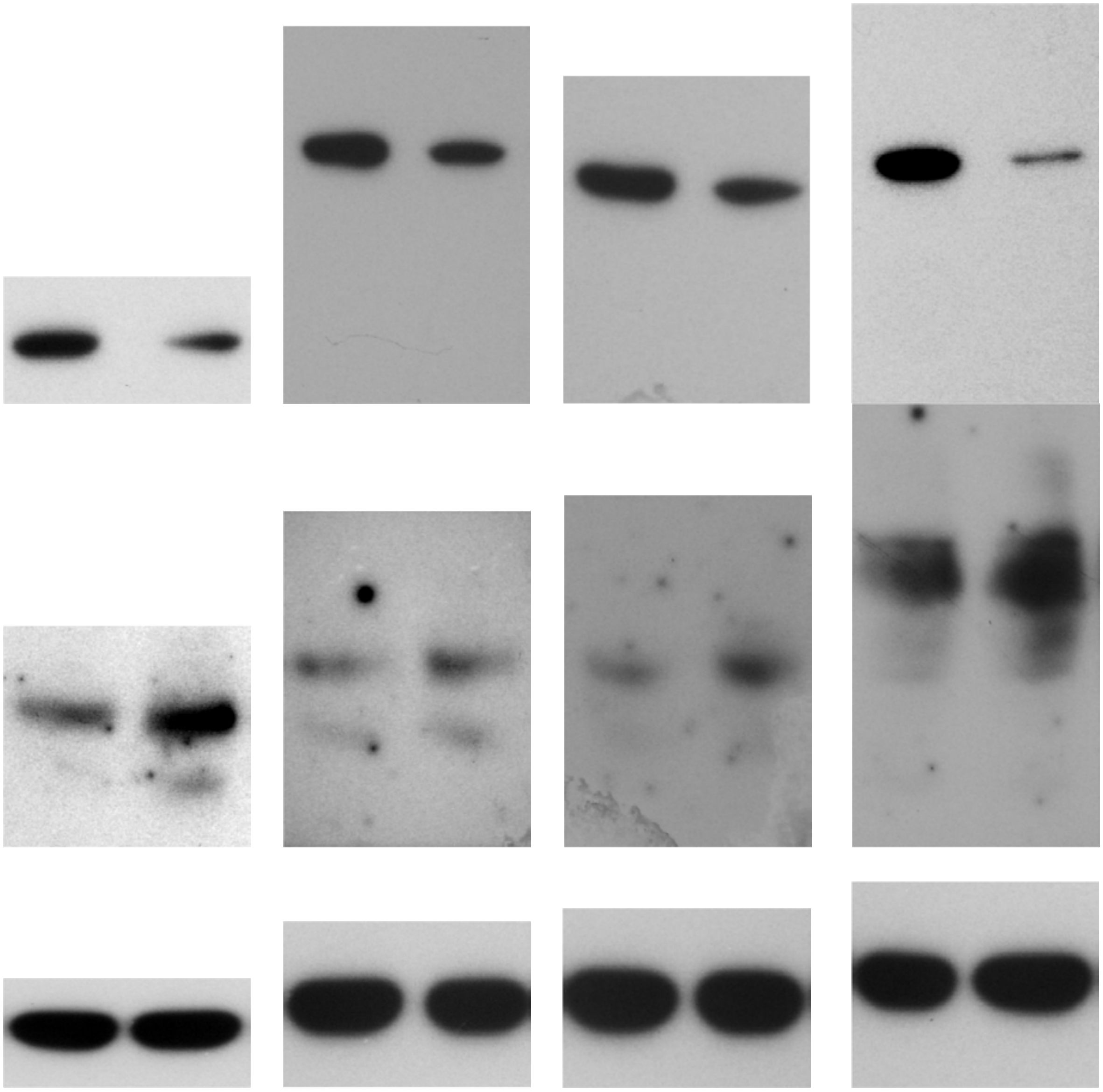

Fig. 5B

EZH2-CASZ1 / actin

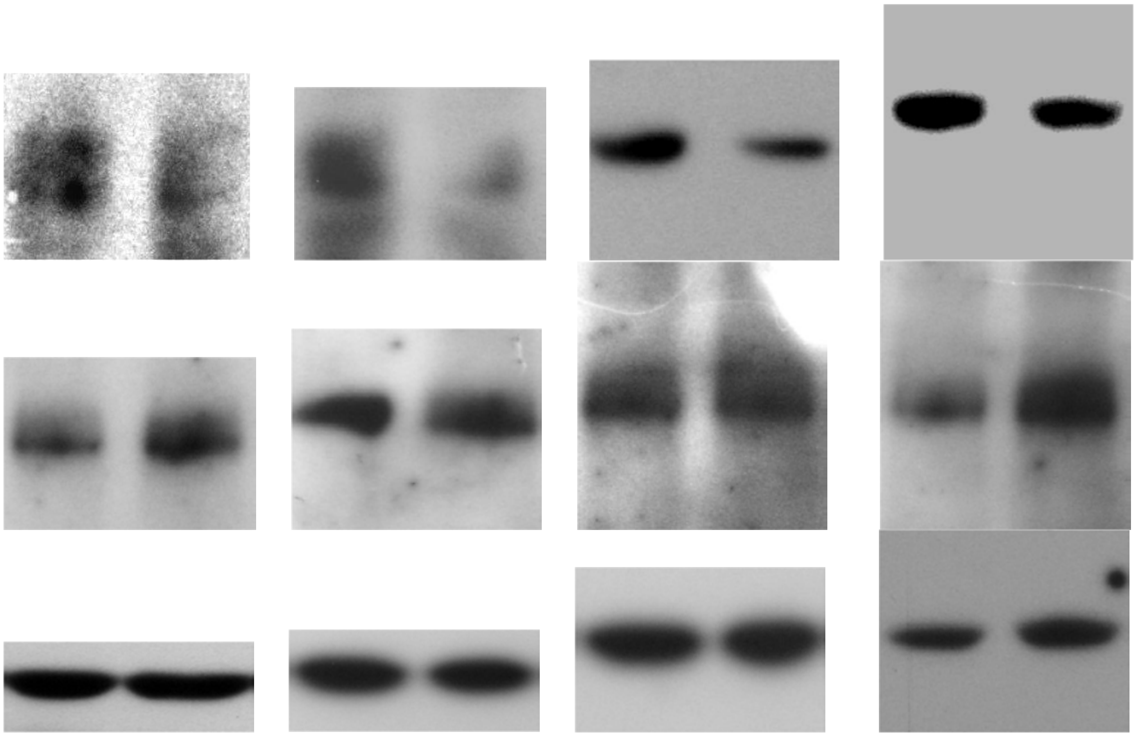

Fig. 5C
EZH2 – CASZ1 / actin

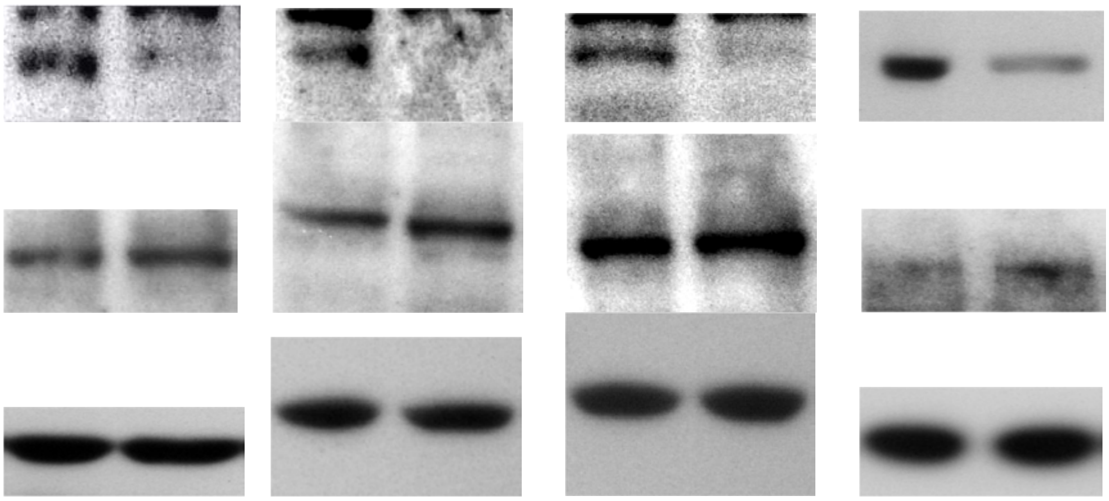

Fig. 5D

p75NTR / actin

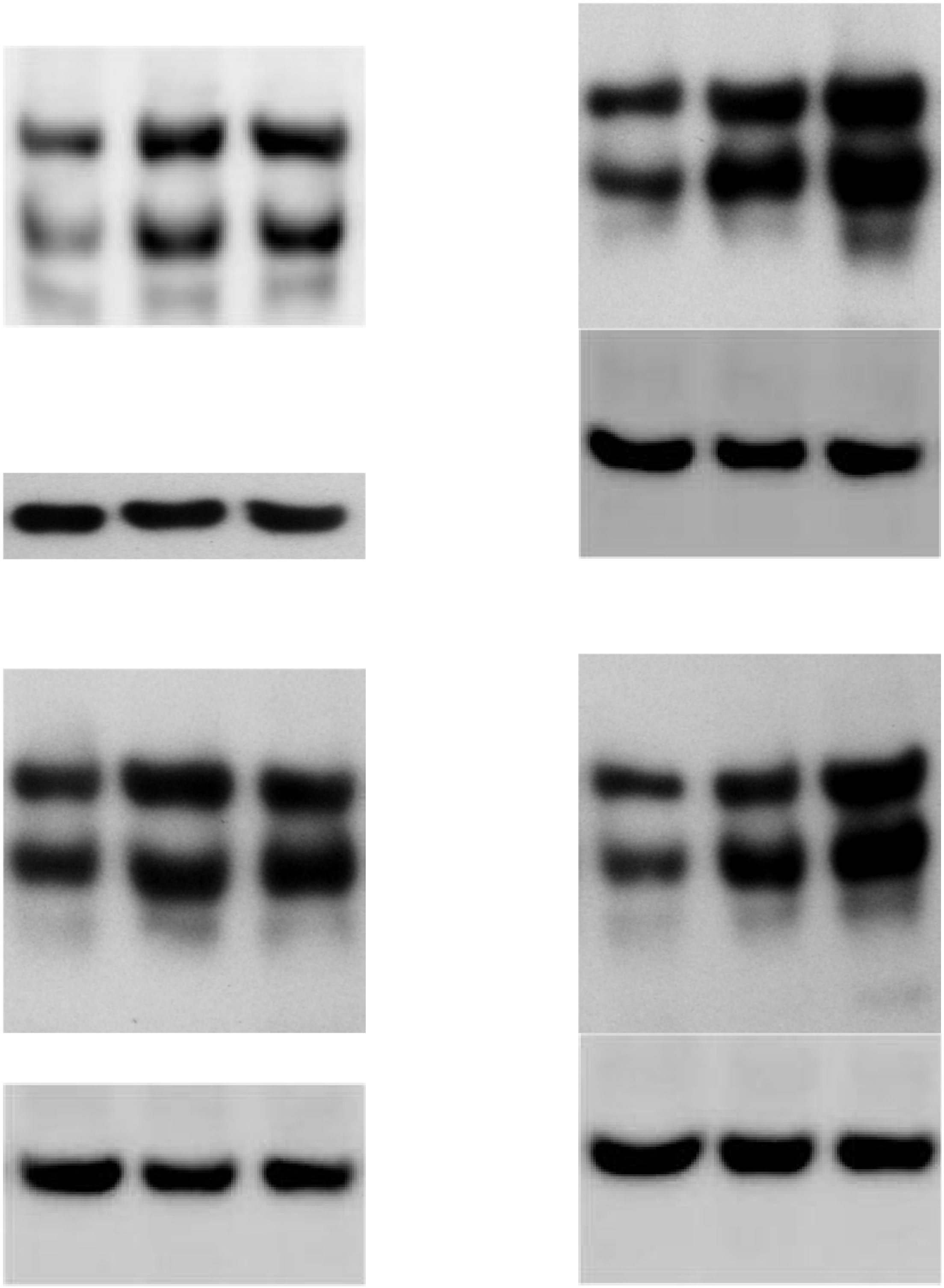

Fig. 5E

CASZ1 – p75NTR / actin

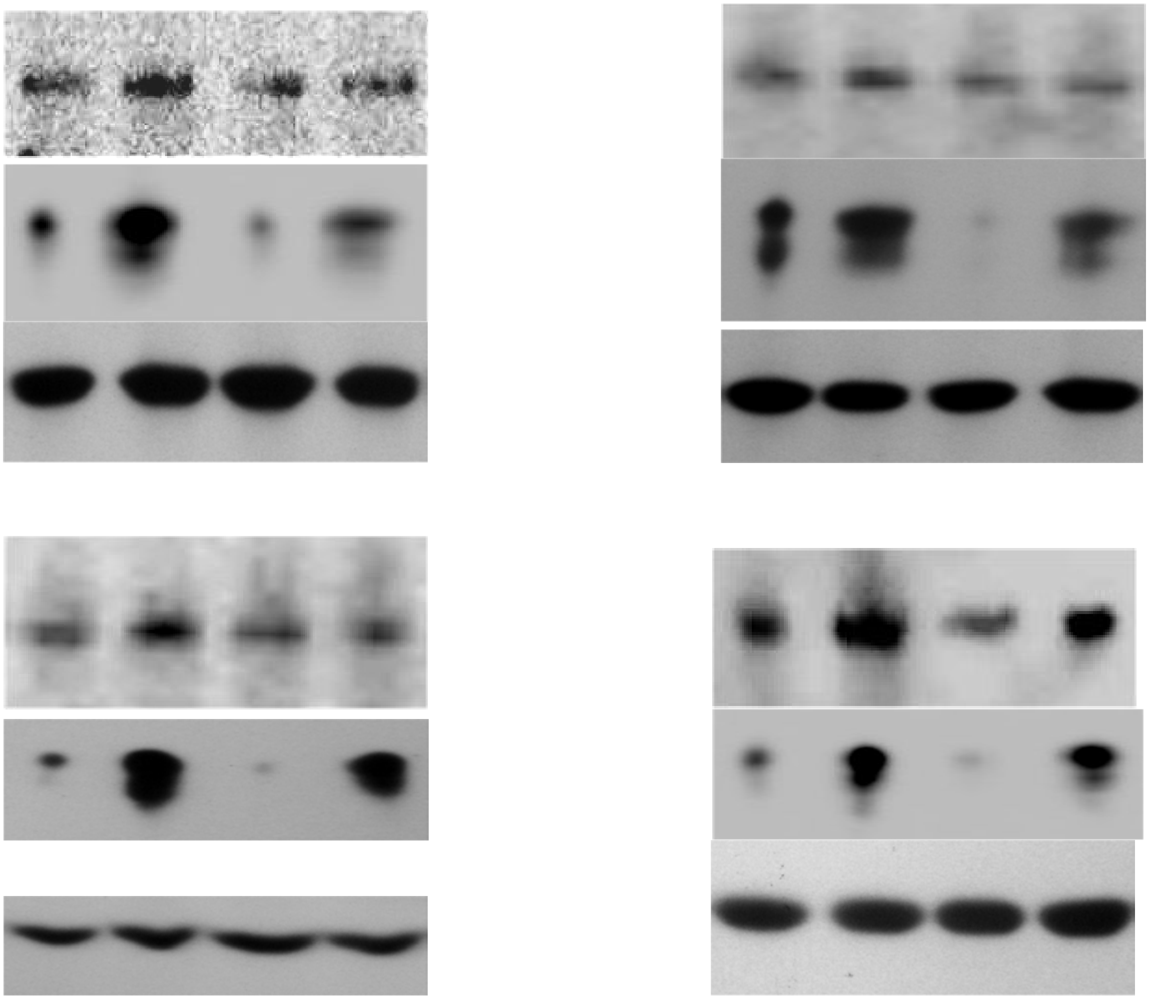

Fig 6B

pJNK / JNK

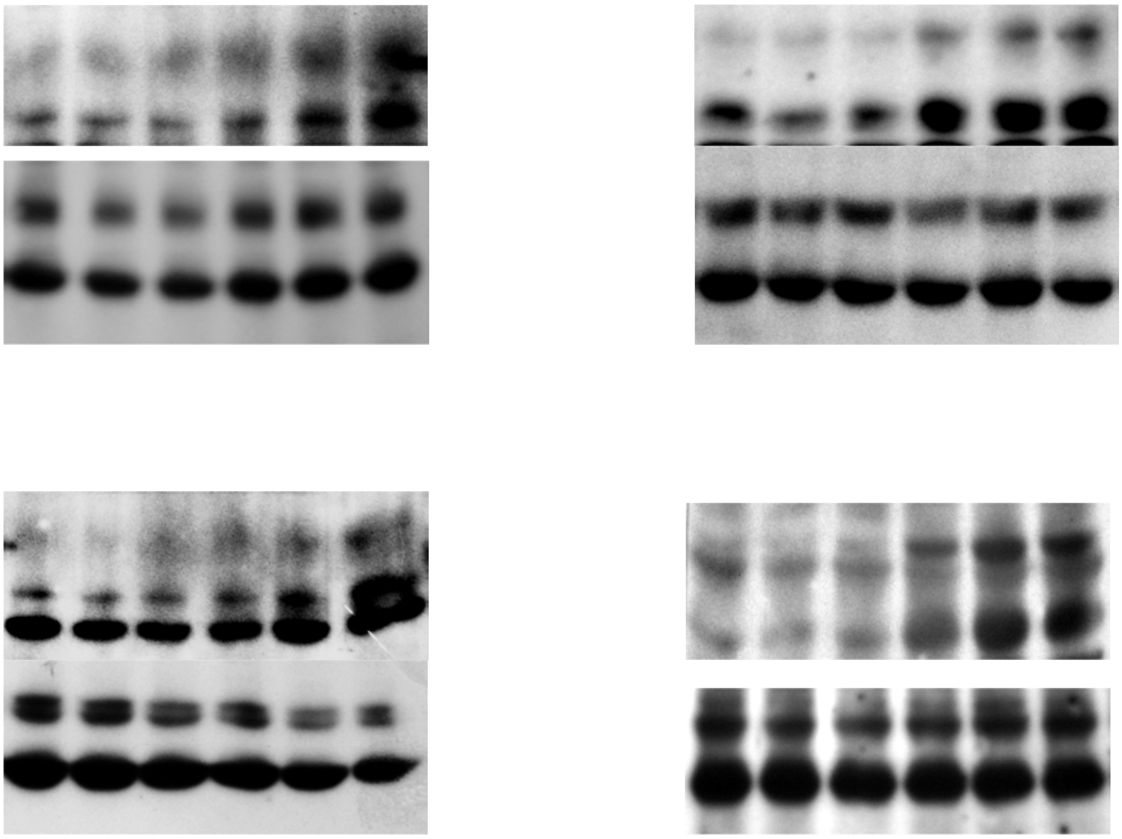

Fig. 6C

p-c-Jun / c-Jun

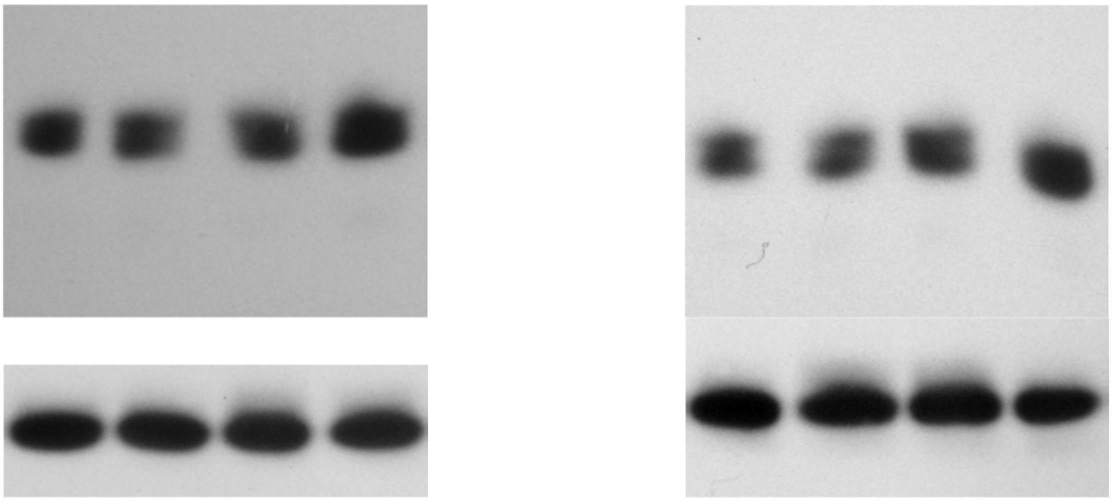

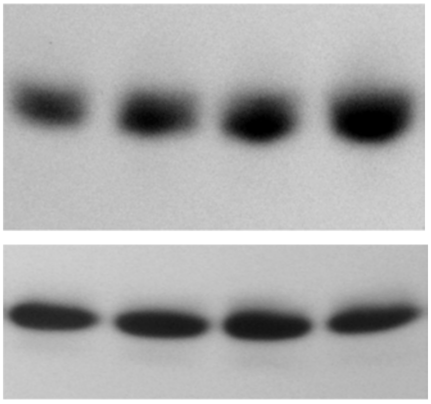

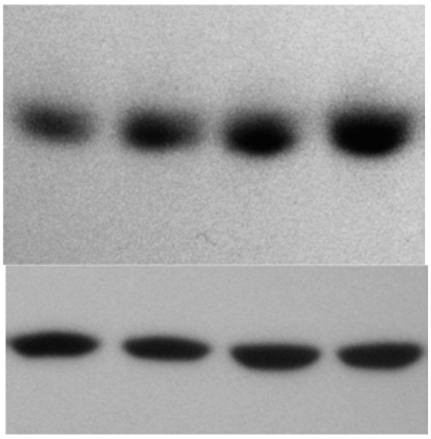

Fig. 7C - SH-SY5Y-

cleav casp 9 /procasp 9

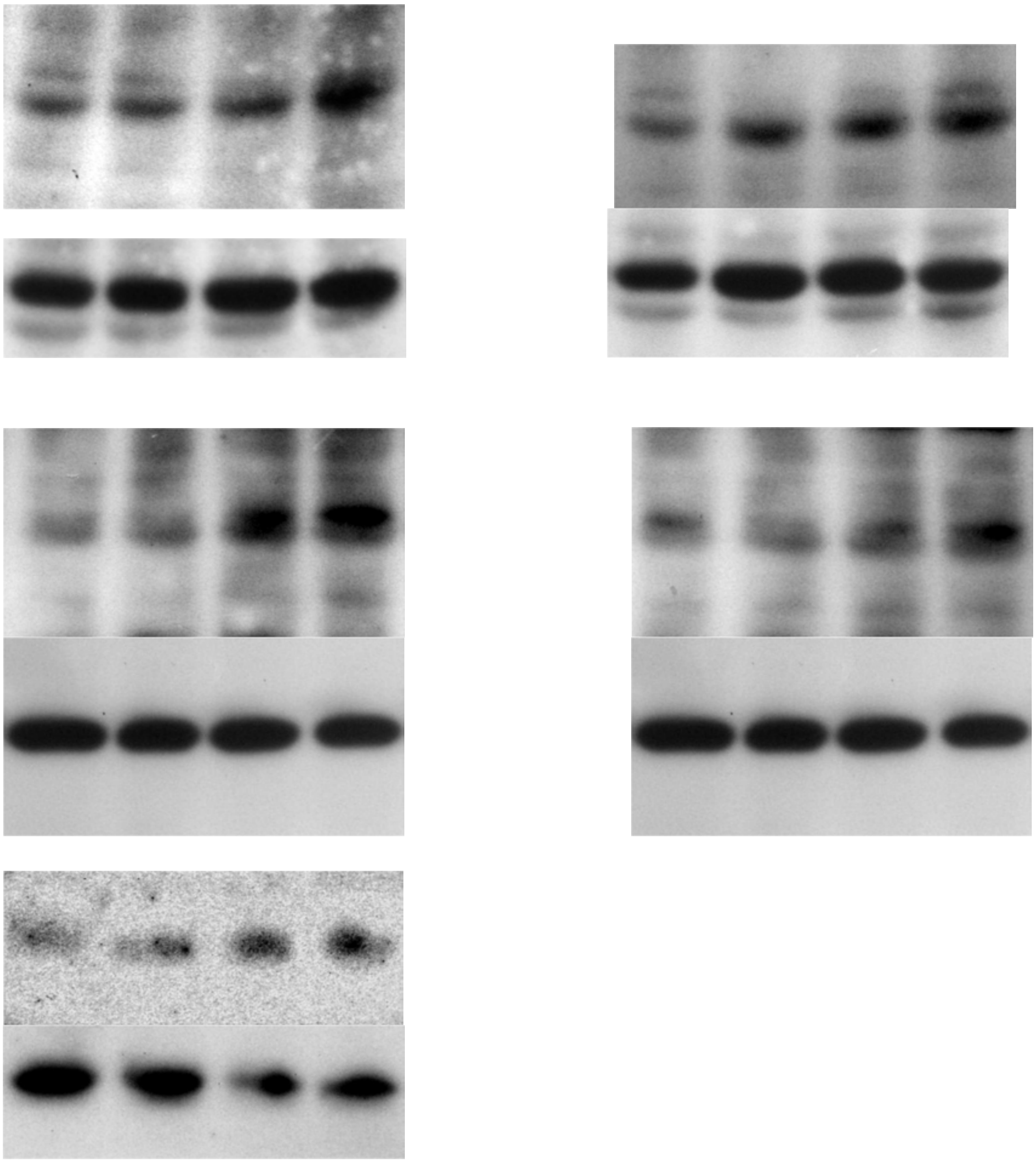

cleav casp 3 / procasp 3

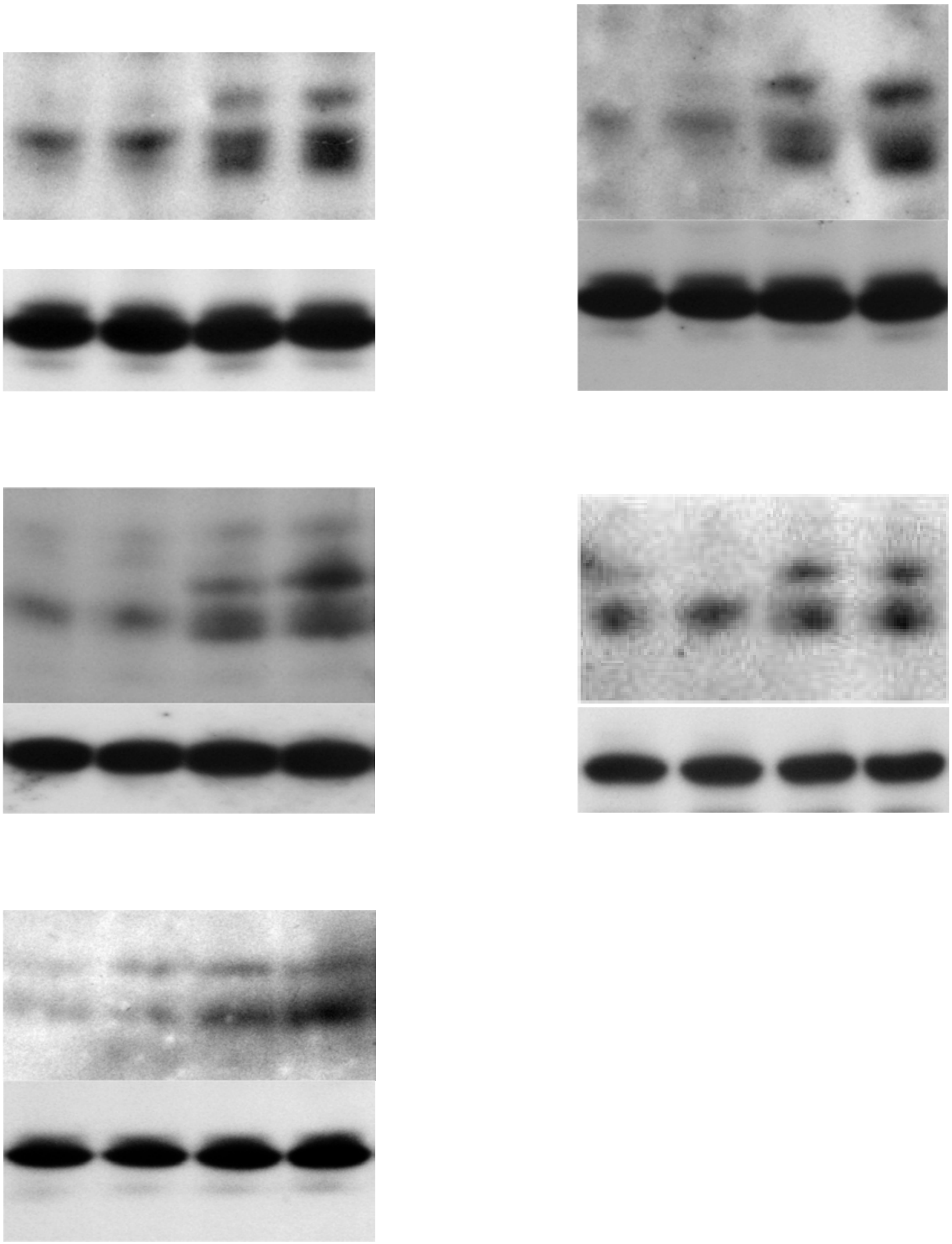

cleav PARP / PARP

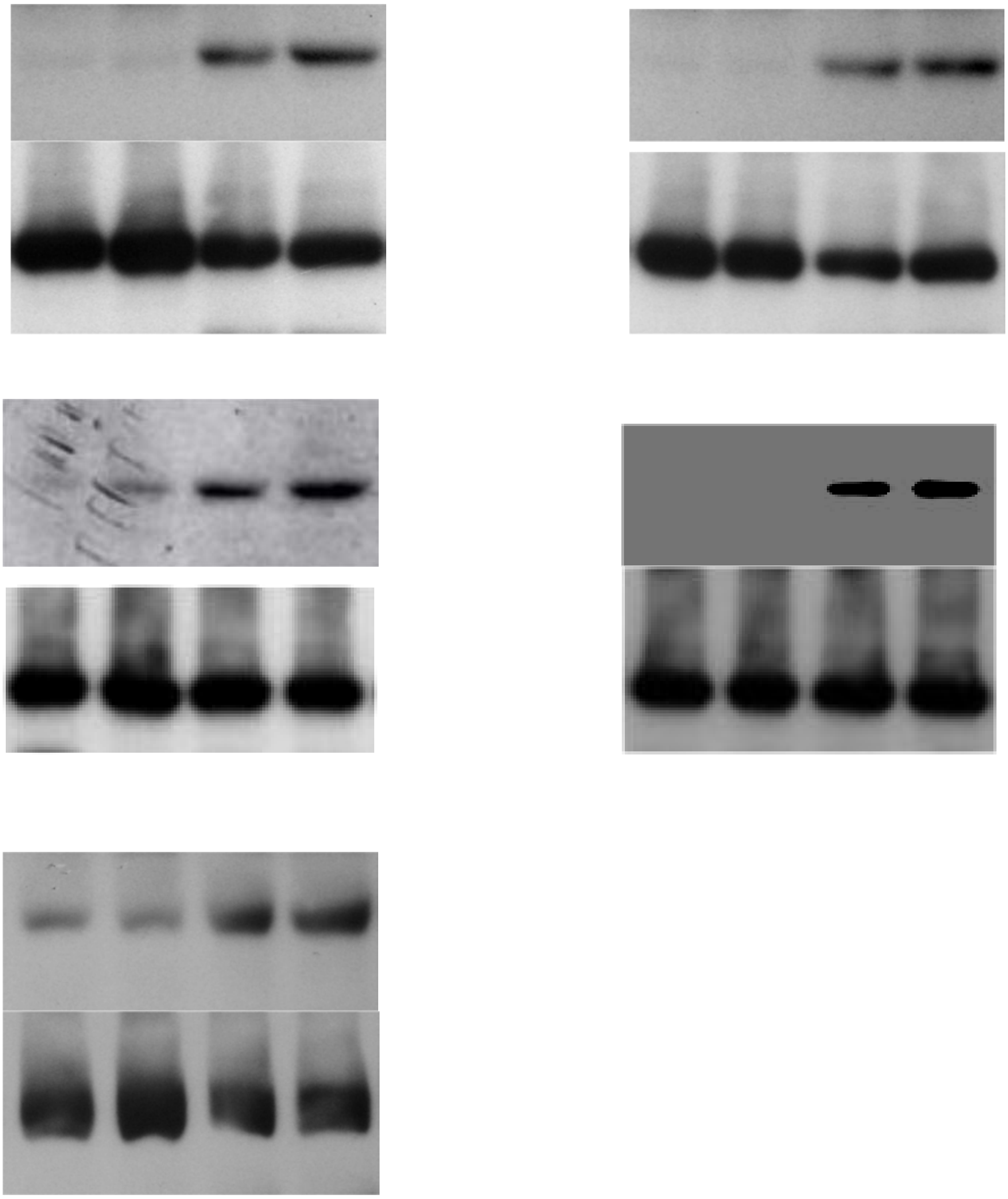

Fig. 7C - LAN 1-

cleav casp 9 / procasp 9

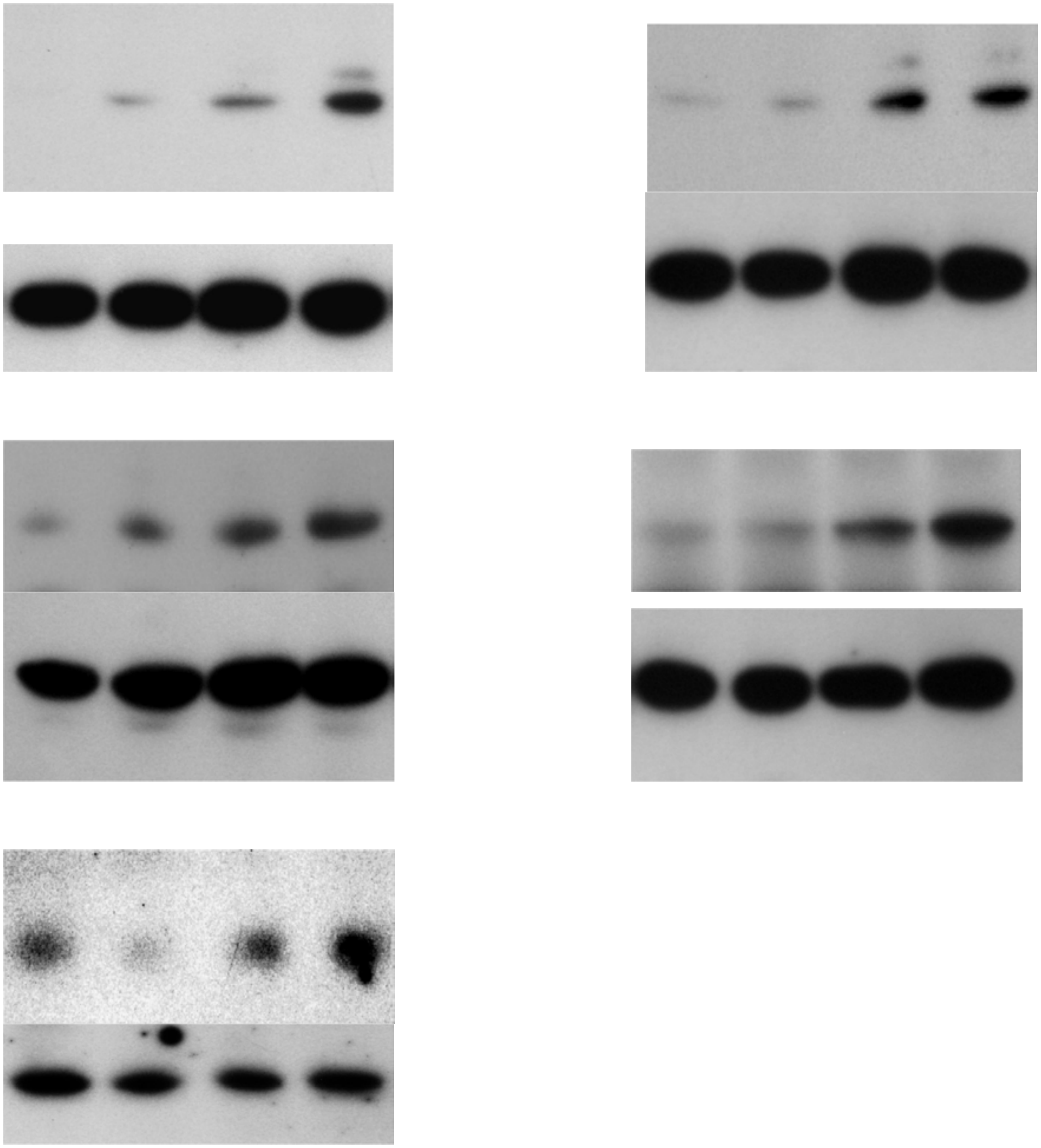

cleav casp 3 / procasp 3

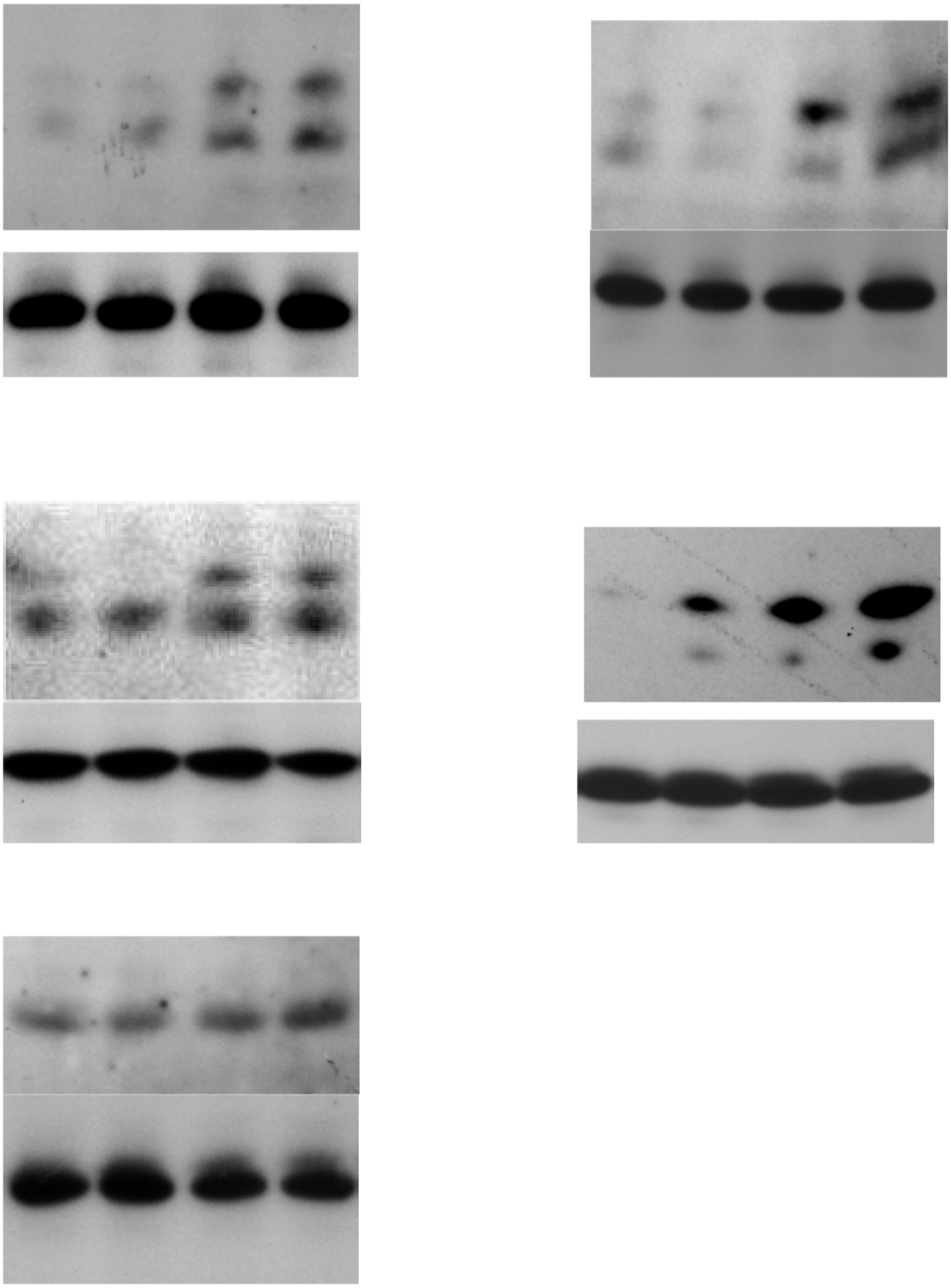

cleav PARP / PARP

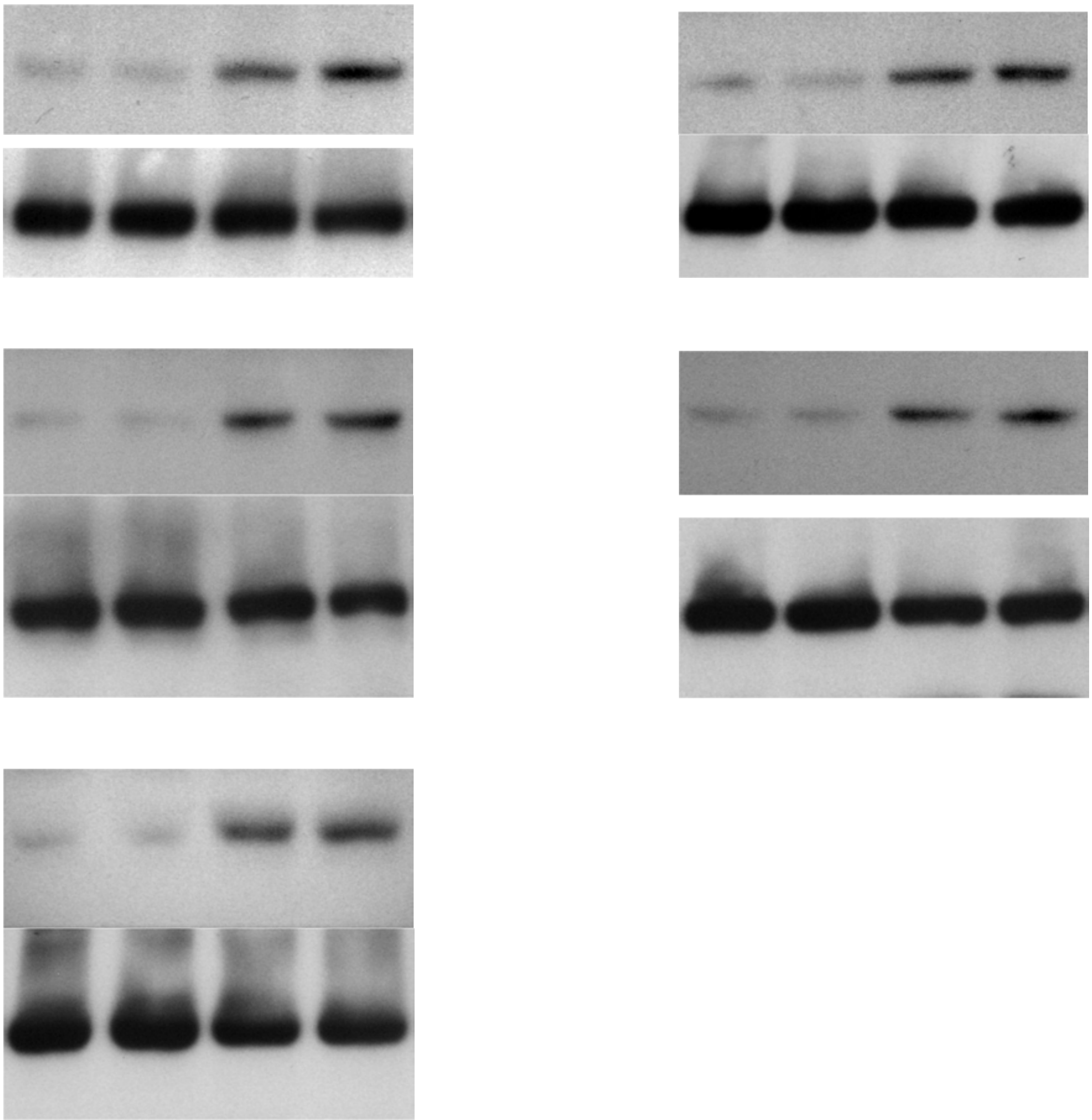

Fig, 8A

p75NTR / actin

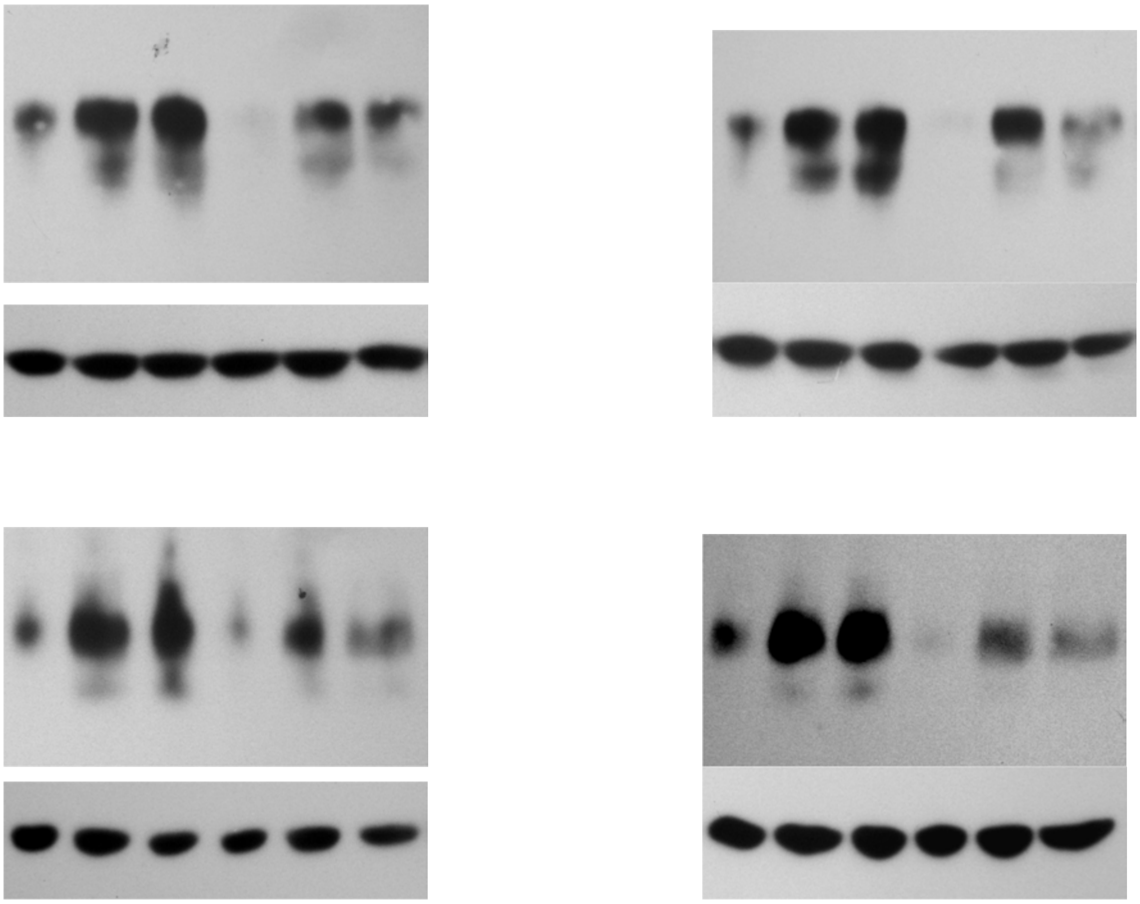

pJNK / JNK

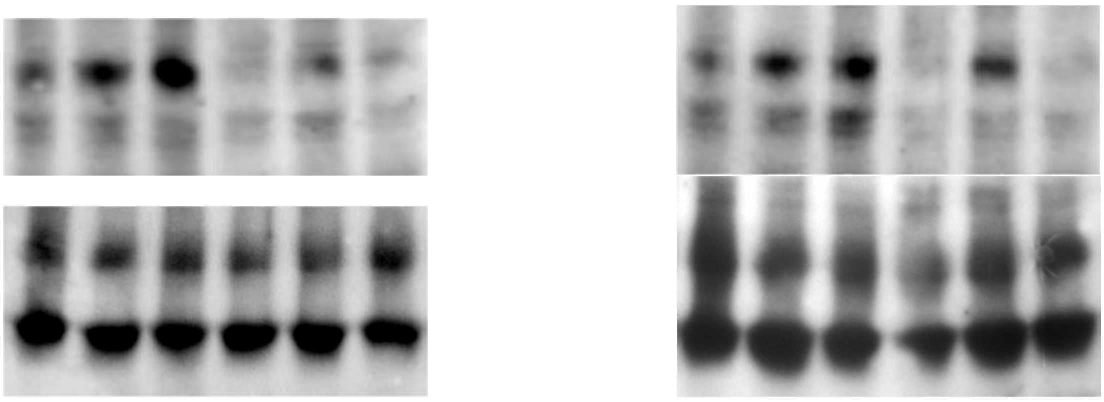

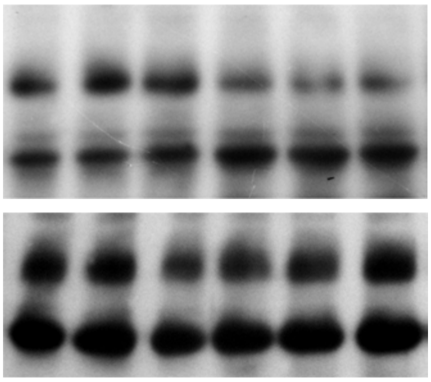
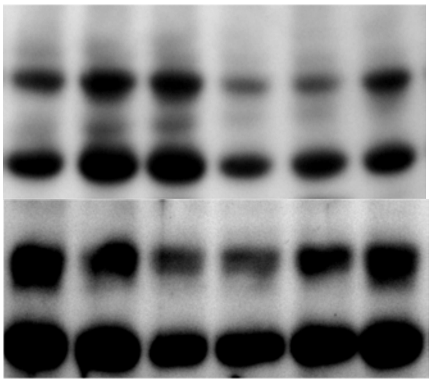

cleav PARP / PARP

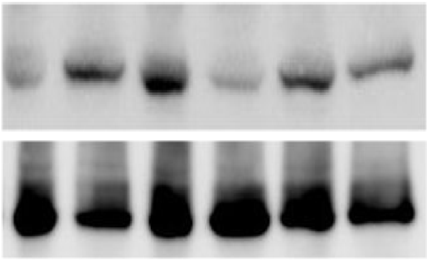
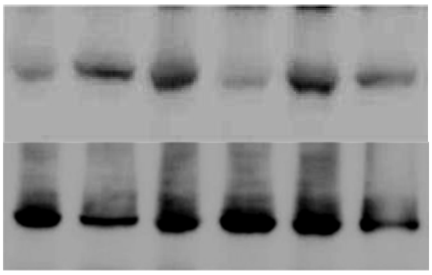
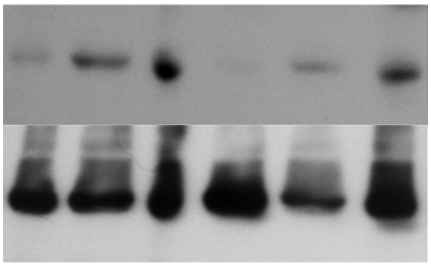
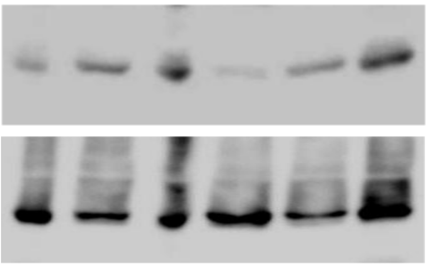

Fig. 8B

cleav PARP / PARP

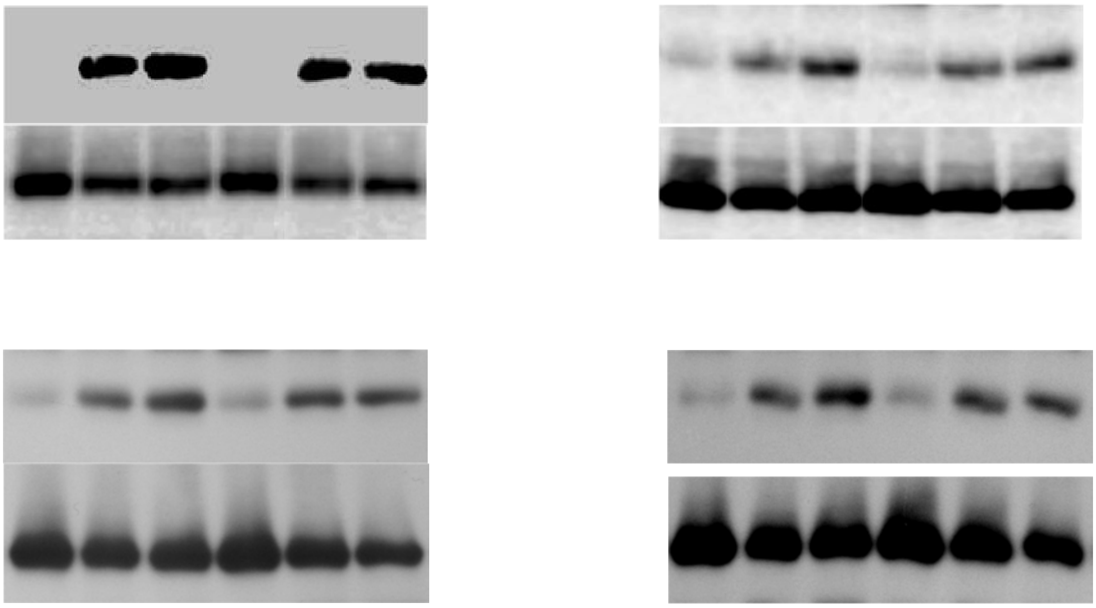

Fig. 9A

p75NTR / actin

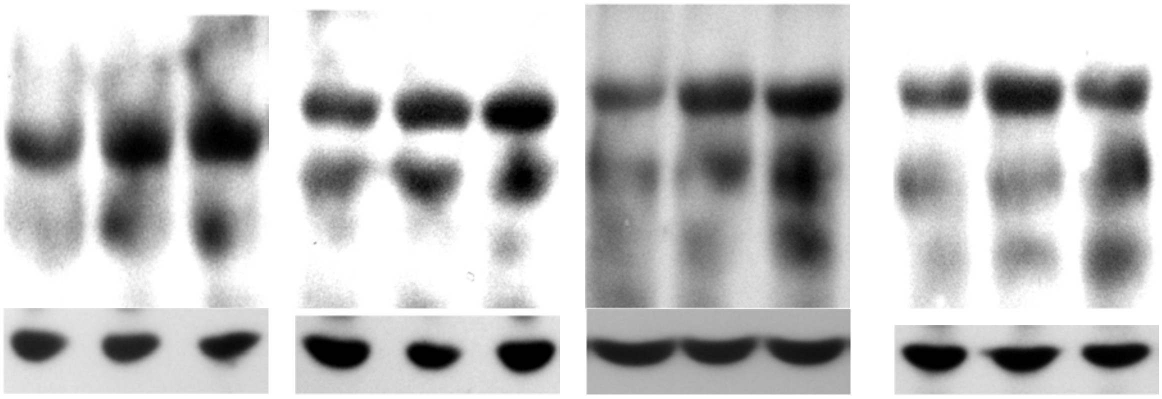

sortilin / actin

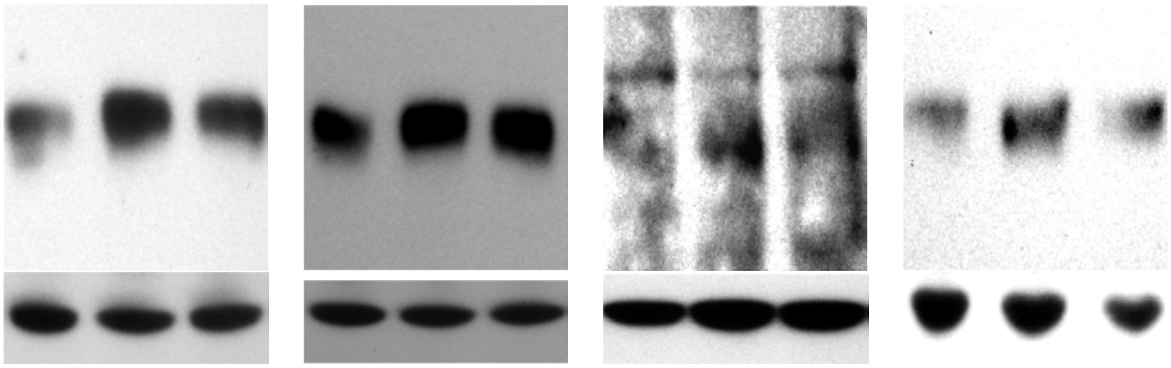

Supplement: Supplementary file 1 — Electronic supplementary material 1 (PDF 9868 kb) [file 10495_2020_1626_MOESM1_ESM.pdf]
